# Non-commutative $L^p$ spaces and Grassmann stochastic analysis

**DOI:** 10.1007/s00440-025-01379-4

**Published:** 2025-05-25

**Authors:** Francesco De Vecchi, Luca Fresta, Maria Gordina, Massimiliano Gubinelli

**Affiliations:** 1 Department of Mathematics, University of Pavia, Via Adolfo Ferrata 5, 27100 Pavia, Italy; 2 Mathematics and Physics department, Università degli Studi Roma Tre, L.go S. Leonardo Murialdo 1, 00146 Roma, Italy; 3 Department of Mathematics, University of Connecticut, Storrs, CT 06269 USA; 4 Mathematical Institute, University of Oxford, Woodstock Road, Oxford, OX2 6GG UK

**Keywords:** Non-commutative probability, Tomita–Takesaki theory, Haagerup spaces, Quantum stochastic calculus, Euclidean quantum field theory, Stochastic quantization, 81S25, 46L53, 60H17, 81S20

## Abstract

We introduce a theory of non-commutative $L^p$ spaces suitable for non-commutative probability in a non-tracial setting and use it to develop stochastic analysis of Grassmann-valued processes, including martingale inequalities, stochastic integrals with respect to *Itô–Grassmann* processes, Girsanov’s formula and a weak formulation of Grassmann SDEs. We apply this new setting to the construction of several unbounded random variables including a Grassmann analog of the $\Phi ^4_2$ Euclidean QFT in a bounded region and weak solution to singular SPDEs in the spirit of the early work of Jona-Lasinio and Mitter on the stochastic quantisation of $\Phi ^4_2$.

## Introduction

We continue to develop Grassmann probability and the associated stochastic analysis initiated in  [1, 16] and motivated by applications to stochastic quantization of Euclidean fermionic quantum field theories. The starting point is the observation in  [1] that the analysis of the renormalized Wick powers of Grassmann Euclidean free fields does not fit naturally in the $C^{*}$-algebraic framework of non-commutative probability. Intuitively, this corresponds to the fact that while linear functionals in Grassmann algebras can be represented as bounded operators on a Hilbert space, this does not seem possible for higher Wick monomials which instead behave as unbounded operators very much like their bosonic counterparts. A similar observation has recently been made also by Chandra–Hairer–Peev [13] where the authors develop a setting for “almost sure” analysis of these unbounded random variables via the notion of *locally* $C^{*}$-*algebras*. While this localization is a useful concept, applications requires one to take averages of these unbounded elements and to estimate or compute their moments. More generally, our goal is to develop Grassmann probability and stochastic analysis analogously to their bosonic counterparts; this involves adjusting an existing setting in algebraic probability, which, until now, has lacked profound applications in stochastic analysis.

In this paper we introduce appropriate notions of Banach spaces of non-commutative random variables whose *p*-th powers are integrable, i.e., the equivalent of the standard $L^p$ spaces associated with a classical probability space $(\Omega , \mathcal {F}, \mathbb {P})$ based on a probability measure $\mathbb {P}$ on a measurable space $(\Omega , \mathcal {F})$. Segal  [48] recognized quite early the possibility to introduce a useful generalization of a measure to the *non-commutative* setting of a $C^{*}$-algebra endowed with a *tracial* state $\tau$ . See also the lucid account by Nelson  [40]. Unfortunately, the tracial setting does not allow for the embedding of non-trivial Grassmann random variables, since the tracial nature of the state forces the covariance to be zero due to the contrasting requirements of symmetry (by the tracial condition) and antisymmetry (by their Grassmann nature). Following the early work of Osterwalder–Schrader  [42], Grassmann Gaussian variables of  [1] are constructed over the Fock state of a pair of canonical anticommutation relation (CAR) algebras. This state is also not suitable to develop an appropriate $L^p$ theory since it is not faithful for the underlying $C^{*}$-algebra. We are bound, therefore, to look for appropriate states elsewhere with the basic desiderata that they need to represent the expectation of Grassmann Gaussian random variables (and processes or, more generally, fields) and be faithful. Our proposal is to realize Grassmann Gaussian probability over a non-commutative probability space given by a large class of non-Fock (and non-tracial) quasi-free states of the CAR algebra, and in particular by the Araki–Wyss factors  [6]. These algebras are obtained in the GNS representation associated with a quasi-free state  [5, 8], so that we can extend quite naturally the Osterwalder–Schrader embedding to them in order to represent arbitrary Grassmann Gaussians. We also notice that they are factors of type III, so that our construction proves to be a further significant application of such factors in mathematical physics  [30, 63].

The associated Tomita–Takesaki modular automorphism gives rise, via a standard construction due to Haagerup  [21], to an appropriate notion of non-commutative $L^p (\mathcal {M})$ spaces in this non-tracial setting. Non-tracial $L^p$ spaces have been extensively studied in the literature  [44], in particular standard martingale inequalities are available, see, e.g., the work of Pisier–Xu  [46], Junge  [27], Junge–Xu  [28], and hypercontractivity of the Araki–Wyss factors has been proved by Lee–Richard  [36] in the greater generality of the *q*-Gaussian setting introduced by Hiai  [23]. However, despite this extensive literature, the use of these non-tracial $L^p$ spaces in non-commutative probabilistic *applications* (other than proving inequalities) has remained underdeveloped mainly because of their abstract nature. While Haagerup’s theory is quite efficient and tries to bring the analysis to the tracial setting (via a crossed-product construction originally due to Connes  [14]), this happens at the price of the appearance of many complications. Various reformulations of the $L^p$ theory have been proposed, in particular by Connes–Hilsum  [25], Araki–Masuda  [2] and more concretely, with applications to Markovian semigroups in quantum statistical mechanics by Majewski–Zegarliski  [38].

The main difficulty is that there is no canonical identification of elements of the von Neumann algebra (vNa) $\mathcal {M}$ in the $L^p$ space and as a consequence there is no canonical way to extend the algebra product into a continuous bilinear map compatible with the expected Hölder inequality. Another difficulty is related to the abstract nature of these spaces, whereas in non-commutative probability it would be appropriate to identify normal unbounded elements affiliated with the vNa $\mathcal {M}$ with unbounded complex-valued random variables (i.e. operators on a Hilbert space). No such identification is possible, to our knowledge, in general for the normal elements of the $L^p$ spaces. Some early ideas on how to associate concrete closed unbounded operators on a Hilbert space to elements of $L^2$ spaces of general quasi-free states of CAR and CCR algebras are present in the pioneering wo rk of Barnett–Streater–Wilde  [10], which follows their systematic development of the $L^2$-based stochastic calculus in the tracial CAR setting  [9]. See also the follow-up work of Wilde  [62], and  [18, 19] for further work on the tracial $L^2$-based stochastic calculus. In  [10] the authors identify some additional conditions which allow one to associate a closed operator to a subset of elements of $L^2$. However, the work of these authors is restricted to the $L^2$ setting and due to the lack of a natural product structure, it does not allow them to construct a fully fledged stochastic calculus.

Considering that our goal is to build applicable tools based on the previously established results in algebraic probability, our main contributions can be summarized as follows.

- We define Banach spaces $\mathbb {L}^p$ (twisted $L^p$ spaces) in such a way that they densely contain the algebra $\mathcal {M}_a$ of analytic elements of $\mathcal {M}$. These spaces are constructed via Haagerup’s $L^p$ spaces and for which the product in $\mathcal {M}_a$ extends canonically to a continuous bilinear map $\mathbb {L}^p \times \mathbb {L}^q \rightarrow \mathbb {L}^r$ for the standard Hölder relation among exponents $1 / p + 1 / q = 1 / r$ (Sect. 2.3).
- We extend standard non-commutative probabilistic results to these spaces, including martingale inequalities and hypercontractivity of Gaussian random variables (in particular Grassmann-valued).
- Given a filtered probability space supporting a Grassmann Brownian motion, we construct natural notions of $\mathbb {L}^p$-valued Itô stochastic integrals and Itô processes (Sect. 3) and prove an Itô formula for polynomial functionals of Itô processes (Sect. 5.2).
- We prove a Grassmann version of Lévy’s martingale characterization of Brownian motion, Girsanov’s theorem (Sect. 5.3) and give a notion of weak solutions to SDEs (Sect. 5.4)
- We use the above tools to construct various examples of unbounded random variables in the Grassmann context, in particular:
  A Grassmann analog of the $\Phi ^4_2$ measure in a bounded domain (Sect. 6.1);
  A Grassmann analog of a Langevin SPDE driven by a renormalized cubic polynomial (Sect. 6.2);
- We identify conditions under which elements in $L^p$ can be canonically interpreted as unbounded operators on a Hilbert space $\mathcal {H}$ (here $\mathcal {H}$ can be taken to be $L^2 (\mathcal {M})$ without loss of generality) (Appendix B).

In contrast to the works  [1, 16], which deal with the stochastic quantisation of Grassmann measures via singular SPDEs and forward-backward SDEs respectively, the present work focuses on advancing Grassmann stochastic analysis, including the development of tools like stochastic integration and Girsanov’s formula, and can thus be thought of as complementary to earlier work. In particular, the reader should note that in  [1, 16] an averaged Itô formula proves sufficient for the purposes of stochastic quantisation, without the need of the stochastic integral.

Furthermore, note that, even with these $\mathbb {L}^p$ spaces we are not able to provide a positive answer to the original problem formulated in  [1], namely to solve non-linear singular renormalized Grassmann SPDEs with additive noise. The main difficulty is the lack of an appropriate Banach space where we can find estimates of solutions via fixed-point methods or, alternatively, via appropriate coercive estimates. Informally put “the estimates do not close”, as it is the case also for commutative SPDEs, since $L^p (\Omega , \mathbb {P})$ spaces are not adequate for finding solutions via fixed-point methods. Yet, we find that our investigation uncovered a very interesting and largely unexplored area of non-commutative stochastic analysis. Another unexpected byproduct is the identification of quasi-free modular states (i.e. thermal) as the natural arena for non-commutative stochastic analysis. We plan to address in future work the consequences of this observation outside the Grassmann setting, both for CAR and CCR algebras.

## Non-commutative $L^p$ spaces

In this section, we briefly recall Haagerup’s construction of the non-commutative $L^p$ spaces associated with a general vNa  [21], see also  [58] for a detailed exposition. This construction is based on embedding $\mathcal {M}$ in a larger semifinite vNa obtained as a crossed product with the group $\mathbb {R}$, in which non-trivial elements of the $L^p$ spaces are intrinsically identified with affiliated unbounded operators. Haagerup’s $L^p$ spaces possess desirable properties, but still lack some crucial features needed for applications to stochastic calculus. In Sect. 2.3 we introduce some novel twisted spaces that address this difficulty and will serve our purposes throughout the paper. In the final part of this section, we discuss conditional expectation and martingale inequalities for $L^p$ spaces and their twisted counterpart.

### Modular theory and crossed products

The modular theory by Tomita and Takesaki  [23, 33, 52, 53, 57, 59] is the key that opened the way to understanding factors without traces, that is, of type III, see also  [37]. Let $\mathcal {M}$ be a $\sigma$-finite vNa acting on $\mathcal {H}$, that is, $\mathcal {M}$ has a faithful normal state $\omega$ and, via the GNS representation, it is isomorphic to a vNa that has a cyclic and separating vector  [7].^1^ Without loss of generality, we can simply assume that $\omega (\cdot ) = \left\langle \Omega , \cdot \, \Omega \right\rangle$, with $\Omega \in \mathcal {H}$ cyclic and separating for $\mathcal {M}$, so that $x \mapsto x \Omega$ establishes a bijection between $\mathcal {M}$ and the dense subspace $\mathcal {M} \Omega \subset \mathcal {H}$. The analysis of the operator acting on $\mathcal {H}$ associated with the involution $^{*}$ is the starting point of the Tomita–Takesaki modular theory. We let $S_{\omega }$ be the closure of the anti-linear operator on $\mathcal {M} \Omega$ defined by $S_{\omega } x \Omega :=x^{*} \Omega$ and let $S_{\omega } = : J_{\omega } \Delta _{\omega }^{1 / 2}$ be its polar decomposition. Here $J_{\omega }$ is an anti-unitary operator called *modular conjugation* with the property $J_{\omega }^2 = \mathbb {1}$, whereas $\Delta _{\omega }$ is a positive non-singular self-adjoint operator called *modular operator*. If $\omega$ is a trace, then $\Vert S_{\omega } x \Omega \Vert ^2_{\mathcal {H}} = \Vert x \Omega \Vert ^2_{\mathcal {H}}$, that is, $S_{\omega }$ is an isometry, so that $S_{\omega } = J_{\omega }$ and $\Delta _{\omega } = \mathbb {1}$. More generally, the modular operator reflects the non-tracial character in some way, as will be made precise below.

A crucial result is Tomita’s fundamental theorem  [53, 59].

#### Theorem 2.1

(Tomita 1967). $J_{\omega } \mathcal {M}J_{\omega } =\mathcal {M}'$ and $\Delta _{\omega }^{\textrm{i}t} \mathcal {M} \Delta _{\omega }^{- \textrm{i}t} =\mathcal {M}$ for any $t \in \mathbb {R}$, $\mathcal {M}'$ denoting the commutant of $\mathcal {M}$.

As a consequence, one can introduce a one-parameter group of $^{*}$-automorphism $\mathbb {R} \ni t \mapsto \sigma _t^{\omega }$ called the *modular automorphism group* associated with the pair $(\mathcal {M}, \omega )$ and defined by

$$\begin{aligned} \sigma _t^{\omega } (x) = \Delta _{\omega }^{\textrm{i}t} x \Delta _{\omega }^{- \textrm{i}t}. \end{aligned}$$

If $\Delta _{\omega }$ is bounded, then one has the stronger condition $\Delta _{\omega }^z \mathcal {M} \Delta _{\omega }^{- z} =\mathcal {M}$ for any $z \in \mathbb {C}$. Otherwise, it is useful to introduce the set of analytic elements in the vNa.

#### Definition 2.2

The set of entire elements $\mathcal {M}_a \subset \mathcal {M}$ is such that $x \in \mathcal {M}_a$ iff $z \mapsto \sigma _z^{\omega } (x)$ extends to an entire $\mathcal {M}$-valued function.

Note that $\mathcal {M}_a$ is a $w^{*}$-dense and $\sigma ^{\omega }$-invariant $^{*}$-sub-algebra of $\mathcal {M}$. The modular automorphism group makes the non-traciality of $\omega$ quantitative precise via the KMS condition (or, more precisely, the $\beta = - 1$ KMS condition  [8]):

$$\begin{aligned} \omega (x \sigma ^{\omega }_{- \textrm{i}} (y)) = \omega (yx), \qquad \forall x, y \in \mathcal {M}_a, \end{aligned}$$

showing again that $\omega$ is a trace iff $\Delta _{\omega } = \mathbb {1}$.

We now introduce the cross product of the vNa with the modular automorphism group  [7, 14, 55]. This larger vNa turns is at the heart of Haagerup’s construction of the non-commutative $L^p$ spaces associated with an arbitrary $\sigma$-finite vNa. Such non-commutative integration spaces were first introduced by Dixmier, Segal and Kunz in the tracial setting  [17, 34, 48] and later extended to an arbitrary vNa by Haagerup  [21]. See also the work of Kosaki  [31], of Araki–Masuda  [2] for alternative definitions and the work of Trunov–Sherstnev  [60, 61] for preliminary work on $L^1$ spaces for general vNas.

Given the pair $(\mathcal {M}, \omega )$ with $\omega$ modular, the crossed product $\widetilde{\mathcal {M}} :=\mathcal {M} \otimes _{\sigma ^{\omega }} \mathbb {R}$ is the vNa acting on $L^2 (\mathbb {R}, \mathcal {H})$ and generated by the operators $(\pi (x))_{x \in \mathcal {M}}$ and $(\lambda (s))_{s \in \mathbb {R}}$ defined by

$$\begin{aligned} (\pi (x) \xi ) (t) = \sigma ^{\omega }_{- t} (x) \xi (t), \qquad (\lambda (s) \xi ) (t) = \xi (t - s), \qquad \forall \xi \in L^2 (\mathbb {R}, \mathcal {H}), \quad a.e. \quad t \in \mathbb {R}. \end{aligned}$$

Note that $\pi$ is a normal faithful representation of $\mathcal {M}$ on $L^2 (\mathbb {R}, \mathcal {H})$ and $\lambda$ gives the modular automorphism group

2.1 $$\begin{aligned} \pi (\sigma ^{\omega }_t (x)) = \lambda (t) \pi (x) \lambda (t)^{*}, \qquad x \in \mathcal {M}, \quad t \in \mathbb {R}, \end{aligned}$$

We will henceforth identify $\mathcal {M} \equiv \pi (\mathcal {M}) \subset \widetilde{\mathcal {M}}$. One can also define a dual automorphism (dual via Fourier transform): since $(\lambda (s))_{s \in \mathbb {R}}$ is the group of translations, one lets $(W (t))_{t \in \mathbb {R}}$ be the unitary representation of $\mathbb {R}$ in $L^2 (\mathbb {R}, \mathcal {H})$ given by $W (t) \xi (s) = \textrm{e}^{- \textrm{i}ts} \xi (s)$ and define

$$\begin{aligned} {\hat{\sigma }}_t (x) = W (t) xW (t)^{*}, \qquad x \in \mathcal {R}, t \in \mathbb {R}. \end{aligned}$$

The following relations hold

$$\begin{aligned} {\hat{\sigma }}_t (x) = x, \qquad {\hat{\sigma }}_t (\lambda (s)) = \textrm{e}^{- i s t} \lambda (s), \qquad x \in \mathcal {M}, \, s, t \in \mathbb {R} \end{aligned}$$

which likewise determine the action of ${\hat{\sigma }}_t$ uniquely. In particular this allows one to identify $\mathcal {M} \equiv \pi (\mathcal {M})$ as the subset of $\widetilde{\mathcal {M}}$ invariant under $({\hat{\sigma }}_t)_{t \in \mathbb {R}}$. For further details on the cross product construction for a general $W^{*}$-dynamical system, see  [7].

It is well-known  [14, 55] that the crossed product $\widetilde{\mathcal {M}}$ is semifinite and it has a normal semifinite faithful trace $\tau$ such that $\tau \circ {\hat{\sigma }}_t = \textrm{e}^{- t} \tau$ for any $t \in \mathbb {R}$. Let ${\overline{P}} (\mathcal {M})$ and ${\overline{P}} (\widetilde{\mathcal {M}})$ denote the set of normal semifinite weights on $\mathcal {M}$ and $\widetilde{\mathcal {M}}$ respectively  [24]. Any $\varphi \in {\overline{P}} (\mathcal {M})$ induces a dual ${\tilde{\varphi }} \in {\overline{P}} (\widetilde{\mathcal {M}})$ which admits a Radon–Nikodym derivative $h_{\varphi }$ with respect to $\tau$  [22],

$$\begin{aligned} {\tilde{\varphi }} (\cdot ) = \tau (h_{\varphi } \cdot ). \end{aligned}$$

The mapping $\varphi \mapsto {\tilde{\varphi }}$ is a bijection of the set of normal semifinite weights on $\mathcal {M}$ to the subset of normal semifinite weights on $\widetilde{\mathcal {M}}$ such that ${\tilde{\varphi }} \circ {\hat{\sigma }}_t = {\tilde{\varphi }}$, $\forall t \in \mathbb {R}$, see  [58]. This allows one to obtain a mapping ${\overline{P}} (\mathcal {M}) \ni \varphi \mapsto h_{\varphi } \in \widetilde{\mathcal {M}}$ with the following properties:^2^

#### Theorem 2.3

The mapping $\varphi \mapsto h_{\varphi }$ extends to a bijection from the predual $\mathcal {M}_{*}$ to the subspace $\left\{ h \in \widetilde{\mathcal {M}} | \, \forall t \in \mathbb {R} \quad {\hat{\sigma }}_t (h_{\varphi }) = \textrm{e}^{- t} h_{\varphi } \right\}$ and satisfies, for any $\varphi \in \mathcal {M}_{*}$ and $x, y \in \mathcal {M}$:

2.2 $$\begin{aligned} h_{x \varphi y} = x h_{\varphi } y, \qquad h_{\varphi ^{*}} = h_{\varphi }^{*}, \qquad | h_{\varphi } | = h_{| \varphi |}. \end{aligned}$$

#### Remark 2.4

Note that for any $\varphi \in \mathcal {M}_{*}$, one can always define its modulus $| \varphi | \in \mathcal {M}_{*}$ by duality, see  [56].

In particular, the dual state ${\tilde{\omega }}$ has Radon–Nikodym derivative with respect to $\tau$ which, following standard notation in the literature, we denote by $D = h_{\omega }$ (and exclusively reserve *D* for this purpose) so that on $\widetilde{\mathcal {M}}_+$ one has^3^

$$\begin{aligned} {\tilde{\omega }} (\cdot ) = \tau (D \cdot ). \end{aligned}$$

It is important to recall that *D* is a positive invertible self-adjoint operator on $L^2 (\mathbb {R}, \mathcal {H})$ affiliated with $\widetilde{\mathcal {M}}$ and

$$\begin{aligned} \lambda (t) = D^{\textrm{i}t}, \qquad t \in \mathbb {R} \end{aligned}$$

which follows from the fact that $\widetilde{\mathcal {M}}$ is semifinite.

### Haagerup’s $L^p$ spaces

Because $\tau$ is a trace on $\widetilde{\mathcal {M}}$, one can form the topological algebra $L^0 (\widetilde{\mathcal {M}}, \tau )$ of $\tau$-measurable operators affiliated with $\widetilde{\mathcal {M}}$, see  [40, 58]. Note that $L^0 (\widetilde{\mathcal {M}}, \tau )$ also includes unbounded operators. Haagerup’s spaces $L^p (\mathcal {M}, \omega )$ are defined as follows  [21]:

#### Definition 2.5

(Haagerup’s $L^p$ spaces). Set

$$\begin{aligned} \begin{array}{ccccc} L^p (\mathcal {M}, \omega ) &  :=&  \left\{ x \in L^0 (\widetilde{\mathcal {M}}, \tau ) | \, {\hat{\sigma }}_t (x) = \textrm{e}^{- \frac{t}{p}} x, \quad t \in \mathbb {R} \right\} &  \qquad &  p \in [1, \infty ),\\ L^{\infty } (\mathcal {M}, \omega ) &  :=&  \left\{ x \in L^0 (\widetilde{\mathcal {M}}, \tau ) | \, {\hat{\sigma }}_t (x) = x, \quad t \in \mathbb {R} \right\} . &  &  \end{array} \end{aligned}$$

#### Remark 2.6

Note that $L^p (\mathcal {M}, \omega )$ is a $^{*}$-invariant linear subspace of $L^0 (\widetilde{\mathcal {M}}, \tau )$. By definition, we also have that $L^p (\mathcal {M}, \omega ) \cap L^q (\mathcal {M}, \omega ) = \{ 0 \}$ if $p \ne q$.

By the considerations in the previous section we have that $L^{\infty } (\mathcal {M}, \omega ) =\mathcal {M}$ and $L^1 (\mathcal {M}, \omega ) \cong \mathcal {M}_{*}$, so that we can equip $L^1 (\mathcal {M}, \omega )$ with the norm of $\mathcal {M}_{*}$. It is then evident that, up to an isometry, for $p = 1, \infty$ $L^p (\mathcal {M}, \omega )$ do not depend on $\omega$, hence it can be dropped from the notation. This can actually be done for any $p \in [1, \infty ]$.

#### Proposition 2.7

Let $x \in L^0 (\widetilde{\mathcal {M}}, \tau )$ with the polar decomposition $x = u | x |$ and let $p \in [1, \infty )$. Then, $x \in L^p (\mathcal {M}, \omega )$ iff $u \in L^{\infty } (\mathcal {M})$ and $| x |^p \in L^1 (\mathcal {M})$.

We shall henceforth write $L^p (\mathcal {M}) \equiv L^p (\mathcal {M}, \omega )$ for any $p \in [1, \infty ]$. As a consequence of Proposition 2.7, the norm on $\mathcal {M}_{*}$ then induces a norm on the $L^p (\mathcal {M})$ spaces; this can also be done via Haagerup’s trace.

#### Definition 2.8

(Haagerup’s trace). On $L^1 (\mathcal {M})$ define the following (positive contractive) linear functional:

$$\begin{aligned} \operatorname {tr}_{\textrm{H}} (h_{\varphi }) :=\varphi (\mathbb {1}). \end{aligned}$$

By the arguments presented in the previous section, we can recover our distinguished state $\omega$ as follows

$$\begin{aligned} \omega (x) = \operatorname {tr}_{\textrm{H}} (Dx) \qquad \forall x \in \mathcal {M}, \end{aligned}$$

where $D = h_{\omega }$. This functional is suggestively called a trace because for any $x \in L^p (\mathcal {M})$ and $y \in L^q (\mathcal {M})$ with $p, q \in [1, \infty ]$ such that $1 / p + 1 / q = 1$, we have $xy, yx \in L^1 (\mathcal {M})$ and

$$\begin{aligned} \operatorname {tr}_{\textrm{H}} (xy) = \operatorname {tr}_{\textrm{H}} (yx). \end{aligned}$$

In particular we also have that for $x \in L^1 (\mathcal {M})$ and $u \in L^{\infty } (\mathcal {M})$ unitary $\operatorname {tr}_{\textrm{H}} (u xu^{*}) = \operatorname {tr}_{\textrm{H}} (x)$, and for $y \in L^2 (\mathcal {M})$ $\operatorname {tr}_{\textrm{H}} (y^{*} y) = \operatorname {tr}_{\textrm{H}} (yy^{*})$.

The property in (2.2) implies that $\operatorname {tr}_{\textrm{H}} (| h_{\varphi } |) = \operatorname {tr}_{\textrm{H}} (h_{| \varphi |}) = | \varphi | (\mathbb {1}) \equiv \Vert \varphi \Vert _{\mathcal {M}_{*}}$, so that $x \mapsto \operatorname {tr}_{\textrm{H}} (| x |)$ is precisely the norm on $L^1 (\mathcal {M})$ induced by $\Vert \cdot \Vert _{\mathcal {M}_{*}}$. We can then define a norm on $L^p (\mathcal {M})$ as follows.

#### Definition 2.9

On $L^p (\mathcal {M})$ for $p \in [1, \infty )$ define

$$\begin{aligned} \Vert x \Vert _{L^p (\mathcal {M})} :=(\operatorname {tr}_{\textrm{H}} (| x |^p))^{\frac{1}{p}} \qquad \forall x \in L^p (\mathcal {M}). \end{aligned}$$

We also set $\Vert \cdot \Vert _{L^{\infty } (\mathcal {M})} :=\Vert \cdot \Vert$.

If no confusion arises we abridge $\Vert \cdot \Vert _{L^p (\mathcal {M})}$ to $\Vert \cdot \Vert _p$. One can prove the following:

#### Theorem 2.10

(Hölder’s inequality). Let $p, q, r \in [1, \infty ]$ such that $1 / p + 1 / q = 1 / r$ and let $x \in L^p (\mathcal {M})$ and $y \in L^q (\mathcal {M})$. Then, $xy \in L^r (\mathcal {M})$ and

$$\begin{aligned} \Vert x y \Vert _r \leqslant \Vert x \Vert _p \Vert y \Vert _q. \end{aligned}$$

Furthermore, for $p \in [1, \infty )$ $(x, y) \mapsto \operatorname {tr}_{\textrm{H}} (xy)$ defines a duality between $L^p (\mathcal {M})$ and $L^q (\mathcal {M})$, $(L^p (\mathcal {M}))^{*} = L^q (\mathcal {M})$ isometrically.

In particular, this duality allows one to prove Minkowski’s inequality so that $\Vert \cdot \Vert _p$ is indeed a norm. The space $L^p (\mathcal {M})$ is complete in the said norm.

#### Theorem 2.11

$(L^p (\mathcal {M}), \Vert \cdot \Vert _p)$ is Banach space for any $p \in [1, \infty ]$. In particular, $L^2 (\mathcal {M})$ is a Hilbert space with scalar product $\langle x, y \rangle _{L^2 (\mathcal {M})} :=\operatorname {tr}_{\textrm{H}} (x^{*} y)$.

Haagerup’s $L^p$ spaces are uniformly convex  [32], see also  [58] for a proof of Clarkson’s inequality.

#### Proposition 2.12

$(L^p (\mathcal {M}), \Vert \cdot \Vert _p)$ is uniformly convex for any $p \in (1, \infty )$.

We have learnt that the only non-trivial elements in $L^p (\mathcal {M})$ are unbounded operators if $p \ne \infty$. The operator *D* is unbounded and it is clear that $D^{\frac{1}{p}} \in L^p (\mathcal {M})$. We also have the following stronger result, see  [28].

#### Lemma

For any $p \in [1, \infty )$ $\mathcal {M}_a D^{\frac{1}{p}}$ is dense in $L^p (\mathcal {M})$ and we have $D^{\frac{1}{2 p} + \tau } \mathcal {M}_a D^{\frac{1}{2 p} - \tau }$=$\mathcal {M}_a D^{\frac{1}{p}}$, for any $\tau \in \mathbb {R}$.

#### Proof

By Hölder’s inequality $\mathcal {M}_a D^{\frac{1}{p}} \subset L^p$. The density is proved by duality: let $y \in L^q$ with $1 = 1 / p + 1 / q$ be such that $\operatorname {tr}_{\textrm{H}} \left( xD^{\frac{1}{p}} y \right) = 0$ for any $x \in \mathcal {M}_a$. By Theorem 2.10, $D^{\tfrac{1}{p}} y \in L^1$ and $\mathcal {M}= (L^1)^{*}$. Since $\mathcal {M}_a$ is $w^{*}$-dense in $\mathcal {M}$ we have $D^{\frac{1}{p}} y = 0$ and thus $y = 0$ by the invertibility of *D*. To prove the identity, we note that if $\forall x \in \mathcal {M}_a$ then $[x]_{- \frac{1}{2 p} - \tau } \in \mathcal {M}_a$ for any $\tau \in \mathbb {R}$ and thus $D^{\frac{1}{2 p} + \tau } \mathcal {M}_a D^{\frac{1}{2 p} - \tau } \ni D^{\frac{1}{2 p} + \tau } [x]_{- \frac{1}{2 p} - \theta } D^{\frac{1}{2 p} - \tau } = xD^{\frac{1}{p}}$, that is, $\mathcal {M}_a D^{\frac{1}{p}} \subset D^{\frac{1}{2 p} + \tau } \mathcal {M}_a D^{\frac{1}{2 p} - \tau }$. The other inclusion is proved in the same way. $\square$

The dense subspaces $\mathcal {M}_a D^{\frac{1}{p}}$ will play a crucial role in our non-commutative stochastic calculus and, more generally, in the calculus of unbounded operators, see Appendix B. This is not at all surprising, given their importance for actual computations, see, e.g.  [27–29].

Before delving into the details of our construction, let us conclude this section by recalling the special case in which $\mathcal {M}$ is semifinite. In this case, $\mathcal {M}$ has a faithful trace, see, e.g., [24], so that one can introduce the tracial $L^p$ spaces  [17, 34, 48]. Let $\tau$ denote the faithful semifinite normal trace on $\mathcal {M}$. Recall that $\mathcal {M} \otimes _{\sigma ^{\tau }} \mathbb {R}$ acts on $L^2 (\mathbb {R}, \mathcal {H}) \cong \mathcal {H} \otimes L^2 (\mathbb {R})$. Let $\mathcal {F} : L^2 (\mathbb {R}) \rightarrow L^2 (\mathbb {R})$ denote the Fourier transform and, for any *f* measurable function on $\mathbb {R}$, let *m*(*f*) denote the multiplication operator by *f* on $L^2 (\mathbb {R})$. We have

$$\begin{aligned} \mathcal {M} \otimes _{\sigma ^{\tau }} \mathbb {R}=\mathcal {M} \otimes \mathcal {F}^{- 1} m (L^{\infty } (\mathbb {R})) \mathcal {F}. \end{aligned}$$

#### Proposition 2.13

Let $\mathcal {M}$ be semifinite, let $\tau$ be the faithful semifinite normal trace on it and let $L^p (\mathcal {M}, \tau )$ denote the tracial $L^p$ space for $p \in [1, \infty )$. Then, the following isometry holds

$$\begin{aligned} L^p (\mathcal {M}) \cong L^p (\mathcal {M}, \tau ) \otimes \mathcal {F}^{- 1} m \left( \textrm{e}^{\frac{\cdot }{p}} \right) \mathcal {F}. \end{aligned}$$

Note that for $p \in [1, \infty )$ $\textrm{e}^{\frac{\cdot }{p}}$ is not bounded and in fact $L^p (\mathcal {M})$ contains only unbounded non-vanishing operators on $\mathcal {H} \otimes L^2 (\mathbb {R})$.

### Twisted $L^p$ spaces

Even though Haagerup’s $L^p$ spaces are very general and elegant, they turn out not to be so convenient for constructing a general non-commutative stochastic calculus. This is ultimately due to the following problems. First of all, unlike the tracial $L^p$ spaces, a scale is completely missing: in fact, we have $L^p (\mathcal {M}) \cap L^q (\mathcal {M}) = \{ 0 \}$ if $p \ne q$. This is intrinsically due to the crossed-product structure, as can be seen directly when $\mathcal {M}$ is semifinite, see Proposition 2.13.

One natural way to recover such a property is to consider the closure of the injection of $\mathcal {M}_a$ into $L^p$, e.g., via $\mathcal {M}_a D^{\frac{1}{p}}$. In fact, if we have $(a_n)_n \subset \mathcal {M}_a$ such that $a_n D^{\frac{1}{p}} \rightarrow A$ in $L^p$, then by Hölder’s inequality it is straightforward to see that $a_n D^{\frac{1}{q}}$ is convergent in $L^q$ for any $q < p$. Note that in  [38] a similar construction was adopted by considering the symmetric injection $\mathcal {M}_a \rightarrow D^{\frac{1}{2 p}} \mathcal {M}_a D^{\frac{1}{2 p}}$ into $L^p$.

Yet, this simple Ansatz is inconvenient when considering products. To see this, let $(a_n), (b_n) \subset \mathcal {M}_a$ be such that $a_n D^{\frac{1}{p}}$ and $b_n D^{\frac{1}{q}}$ converge in the $L^p$ and $L^q$ topologies respectively. But $a_n b_n D^{\frac{1}{r}} = a_n D^{\frac{1}{p}} \sigma _{\textrm{i}\frac{1}{p}} (b_n) D^{\frac{1}{q}}$, where $\sigma _z$ is the analytic continuation of the automorphism group on $\mathcal {M}_a$, so that the product sequence can be controlled only if we can control the twisted sequence $\sigma _{\textrm{i}\frac{1}{p}} (b_n) D^{\frac{1}{q}}$ in the $L^q$ topology. The same issue appears when taking the expectation (and conditional expectations) as well : by the definition of Haagerup’s trace, one has $\omega (a_n b_n) = \operatorname {tr}_{\textrm{H}} \left( a_n D^{\frac{1}{p}} \sigma _{\textrm{i}\frac{1}{p}} (b_n) D^{\frac{1}{q}} \right)$ with $1 = 1 / p + 1 / q$, hence the control of a twisted sequence is needed once more.

This discussion justifies the introduction of some novel “twisted” $L^p$ spaces, as a natural adaptation of the existing setting. First of all, let us denote the analytic continuation of the automorphism group on $\mathcal {M}_a$ by $[\cdot ]_t$, that is, $[x]_t :=\sigma _{- \textrm{i}t} (x)$. Then, we introduce the curve of embeddings $T^{(p)}_t : \mathcal {M}_a \rightarrow L^p (\mathcal {M})$ defined by

2.3 $$\begin{aligned} T^{(p)}_t (x) :=D^{\frac{1}{2 p}} [x]_t D^{\frac{1}{2 p}}, \end{aligned}$$

satisfying $T^{(p)}_t (x^{*}) = (T^{(p)}_{- t} (x))^{*}$. This is indeed an embedding by Lemma 2.2:

#### Corollary 2.14

The map $T^{(p)}_t$ has kernel $\{ 0 \}$ and $T^{(p)}_t (\mathcal {M}_a)$ is dense in $L^p (\mathcal {M})$.

The triviality of the kernel is a straightforward consequence of the invertibility of *D*. We can now introduce the twisted $L^p$ spaces, denoted by $\mathbb {L}^p (\mathcal {M}, \omega )$, as follows:

#### Definition 2.15

Let $\mathcal {M}$ be a vNa, let $\omega$ be a nsf state on $\mathcal {M}$ and let $T^{(p)}_t$ be the embedding of $\mathcal {M}_a$ in $L^p (\mathcal {M})$ as defined in (2.3). We denote by $\mathbb {L}^p (\mathcal {M}, \omega )$ the completion of $\mathcal {M}_a$ with respect to the norm

$$\begin{aligned} \Vert x \Vert _{\mathbb {L}^p (\mathcal {M}, \omega )} :=\sup _{| t | \leqslant 1 - \frac{1}{2 p}} \Vert T^{(p)}_t (x) \Vert _{L^p (\mathcal {M})}. \end{aligned}$$

We abridge the notation to $\mathbb {L}^p \equiv \mathbb {L}^p (\mathcal {M}, \omega )$ and $\Vert \cdot \Vert _{\mathbb {L}^p} = \Vert \cdot \Vert _{\mathbb {L}^p (\mathcal {M}, \omega )}$ if no confusion arises.

This definition is clearly meaningful because $T^{(p)}_t$ is an embedding and because $\Vert \cdot \Vert _{L^p (\mathcal {M})}$ is a norm. With abuse of notation, we henceforth extend the map $T^{(p)}_t$ to the the $\mathbb {L}^p$ spaces as well, $T^{(p)}_t : \mathbb {L}^p (\mathcal {M}, \omega ) \rightarrow L^p (\mathcal {M})$. Therefore, for any $x_n \rightarrow x$ in $\mathbb {L}^p$, we let

$$\begin{aligned} T^{(p)}_t (x) :=L^p - \lim _{n \rightarrow \infty } T^{(p)}_t (x_n), \end{aligned}$$

for any $$| t | \leqslant 1 - \frac{1}{2 p}$$.

We note the following properties.

#### Lemma 2.16

We have:

i. For any $p \in [1, \infty ]$ and $t \geqslant 0$ $\bigcup _{| t' | \leqslant t} T^{(p)}_{t'} (^{} \mathbb {L}^p (\mathcal {M}, \omega ))$ is a $^{*}$-invariant subspace of $L^0 (\widetilde{\mathcal {M}}, \tau )$ and $\Vert x \Vert _{\mathbb {L}^p} = \Vert x^{*} \Vert _{\mathbb {L}^p}$, $\forall x \in \mathbb {L}^p {(\mathcal {M}, \omega )}$.
ii. Let $p, q, r \in [1, \infty ]$ such that $1 / p + 1 / q = 1 / r$. Then, for any $x, y \in \mathcal {M}_a$ we have
  2.4 $$\begin{aligned} T^{(r)}_t (xy) = T^{(p)}_{t + \frac{1}{2 q}} (x) T^{(q)}_{t - \frac{1}{2 p}} (y), \end{aligned}$$
  and
  2.5 $$\begin{aligned} \Vert x y \Vert _{\mathbb {L}^r} \leqslant \Vert x \Vert _{\mathbb {L}^p} \Vert y \Vert _{\mathbb {L}^q}. \end{aligned}$$

#### Proof

The first claim follows from the definition of the extension $T^{(p)}_t : \mathbb {L}^p (\mathcal {M}, \omega ) \rightarrow L^p (\mathcal {M})$, the fact that Haagerup’s $L^p$ spaces are $^{*}$-invariant subspaces of $L^0 (\widetilde{\mathcal {M}}, \tau )$ and $T^{(p)}_t (x^{*}) = (T^{(p)}_{- t} (x))^{*}$. The relation (2.4) is a straightforward computation:

$$\begin{aligned} T^{(r)}_t (xy) = D^{\frac{1}{2 q} + \frac{1}{2 p} + t} xD^{- \frac{1}{2 q} + \frac{1}{2 p} - t} D^{\frac{1}{2 q} - \frac{1}{2 p} + t} yD^{\frac{1}{2 q} + \frac{1}{2 p} - t}. \end{aligned}$$

Finally, we note that if $| t | \leqslant 1 - \frac{1}{2 r}$, then $- 1 + \frac{1}{2 r} + \frac{1}{2 q} \leqslant t + \frac{1}{2 q} \leqslant 1 - \frac{1}{2 p}$, and $- 1 + \frac{1}{2 q} \leqslant t - \frac{1}{2 p} \leqslant 1 - \frac{1}{2 r} - \frac{1}{2 p}$, that is,

$$\begin{aligned} \left| t + \frac{1}{2 q} \right| \leqslant 1 - \frac{1}{2 p}, \qquad \left| t - \frac{1}{2 p} \right| \leqslant 1 - \frac{1}{2 q}, \end{aligned}$$

and thus also (2.5) holds true by Hölder’s inequality for Haagerup’s $L^p$ spaces. $\square$

One would like to extend (2.5) to the whole twisted spaces. To this end, by Lemma 2.16 we extend the product on $\mathcal {M}_a \times \mathcal {M}_a \rightarrow \mathcal {M}_a$ to a product on $\mathbb {L}^p \times \mathbb {L}^q$.

#### Definition 2.17

Define the product $\cdot \, : \mathbb {L}^p \times \mathbb {L}^q \rightarrow \mathbb {L}^r$, for any $p, q, r \in [1, \infty ]$ with $1 / p + 1 / q = 1 / r$

$$\begin{aligned} (x, y) \mapsto x \cdot y :=\mathbb {L}^r - \lim _{n \rightarrow \infty } x_n y_n, \end{aligned}$$

for any $(x_n), (y_n) \subset \mathcal {M}_a$ such that $x =\mathbb {L}^p - \lim _{n \rightarrow \infty } x_n$ and $y =\mathbb {L}^q - \lim _{n \rightarrow \infty } y_n$.

#### Remark 2.18

This product is well-defined thanks to Lemma 2.16. In fact, it is straightforward to check that $x_n y_n$ is a Cauchy sequence in $\mathbb {L}^r$ and that furthermore the limit does not depend on the sequences $(x_n), (y_n) \subset \mathcal {M}_a$.

#### Proposition 2.19

We have:

i. Let $p, q, r \in [1, \infty ]$ such that $1 / p + 1 / q = 1 / r$. Then, for any $x \in \mathbb {L}^p (\mathcal {M})$ and $y \in \mathbb {L}^q (\mathcal {M})$, $x \cdot y \in \mathbb {L}^r (\mathcal {M})$ and Hölder’s inequality holds true
  2.6 $$\begin{aligned} \Vert x \cdot y \Vert _{\mathbb {L}^r} \leqslant \Vert x \Vert _{\mathbb {L}^p} \Vert y \Vert _{\mathbb {L}^q}. \end{aligned}$$
ii. $\mathbb {L}^p (\mathcal {M}) \subset \mathbb {L}^q (\mathcal {M})$ for any $1 \leqslant q \leqslant p \leqslant \infty$ and $\Vert x \Vert _{\mathbb {L}^q} \leqslant \Vert x \Vert _{\mathbb {L}^p}$ for any $x \in \mathbb {L}^p (\mathcal {M})$.

#### Proof

The first claim is a direct consequence of Lemma 2.16 and Definition 2.17. The second statement follows from (2.6) since $\Vert \mathbb {1} \Vert _{\mathbb {L}^p (\mathcal {M})} = (\operatorname {tr}_{\textrm{H}} (D))^{\frac{1}{p}} = \omega (\mathbb {1}) = 1$ for any $p \in [1, \infty ]$. $\square$

#### Remark 2.20

Note that if $x \in \mathbb {L}^p (\mathcal {M})$ and $y \in \mathbb {L}^q (\mathcal {M})$, then their product $x \cdot y$ can be made sense of as the $\mathbb {L}^r$-limit for any $1 / r \geqslant 1 / p + 1 / q$, that is, for any $x_n \rightarrow x$ and $y_n \rightarrow y$ the sequence $x_n y_n$ is convergent in any such $\mathbb {L}^r$ topology. Furthermore, the embedding map $T^{(p)}_t$ can actually be extended to a map from $\mathbb {L}^q (\mathcal {M}) \rightarrow L^p (\mathcal {M})$ for any $q \geqslant p$.

Thanks to Proposition 2.19, we can also extend the expectation $\omega$ to any $\mathbb {L}^p$. In fact, the following lemma holds true.

#### Lemma 2.21

For any $p \in [1, \infty ]$ and $x \in \mathbb {L}^p$, the limit

$$\begin{aligned} \omega (x) :=\lim _{n \rightarrow \infty } \omega (x_n) \end{aligned}$$

exists for any $(x_n)_n \subset \mathcal {M}_a$ such that $x =\mathbb {L}^p - \lim _{n \rightarrow \infty } x_n$ and is independent of the sequence $(x_n)_n$.

#### Proof

We have $| \omega (x_n) - \omega (x_m) | \leqslant \Vert x_n - x_m \Vert _1 \leqslant \Vert x_n - x_m \Vert _{\mathbb {L}^1} \leqslant \Vert x_n - x_m \Vert _{\mathbb {L}^p}$, where in the last step we used Proposition 2.19, so that $\omega (x_n)$ is a Cauchy sequence and thus converges. If $(y_n)_n \subset \mathcal {M}_a$ is another sequence such that $y_n \rightarrow x$ in the $\mathbb {L}^p$ topology, then $| \omega (x_n) - \omega (y_n) | \leqslant \Vert x_n - y_n \Vert _{\mathbb {L}^p}$, so that the limit does not depend on the sequence. $\square$

#### Remark 2.22

It is possible to consider some generalisation of the norm in Definition 2.15: instead of taking the sup norm, with respect to the *t* variable, we can introduce for $q, p \in [1, \infty ]$

$$\begin{aligned} \Vert x \Vert _{\mathbb {L}^p_q (\mathcal {M}, \omega )} :={\left( \int ^{1 - \frac{1}{2 p}}_{\frac{1}{2 p} - 1} \Vert T^{(p)}_t (x) \Vert _{L^p (\mathcal {M})}^q \textrm{d}t \right) ^{\frac{1}{q}}}. \end{aligned}$$

The norm $\Vert \cdot \Vert _{\mathbb {L}^p_q (\mathcal {M}, \omega )}$ gives rise to a Banach space analogous to $\mathbb {L}^p (\mathcal {M}, \omega )$, but in this case the map $T^{(p)}_t$is not usually well-defined on $\mathbb {L}^p_q (\mathcal {M}, \omega )$ (but in some sense this is the case for almost every $t \in \left[ - 1 + \frac{1}{2 p}, 1 - \frac{1}{2 p} \right]$). For such spaces, the product enjoys a Hölder inequality and extends continuously to a mapping $\cdot : \mathbb {L}^p_q (\mathcal {M}, \omega ) \times \mathbb {L}^{p'}_{q'} (\mathcal {M}, \omega ) \rightarrow \mathbb {L}^{p''}_{p''} (\mathcal {M}, \omega )$, provided that $1 / p'' = 1 / p + 1 / p'$ and $1 / q'' = 1 / q + 1 / q'$.

### Conditional expectation and $L^p$ processes

It is a well-known result by Takesaki  [54] that for any vN subalgebra $\mathcal {N} \subset \mathcal {M}$ that is invariant under the modular automorphism group, that is, $\sigma _t (\mathcal {N}) =\mathcal {N}$ $\forall t \in \mathbb {R}$, there exists a conditional expectation $\omega _{\mathcal {N}}: \mathcal {M} \rightarrow \mathcal {N}$ and it satisfies

2.7 $$\begin{aligned} \omega \circ \omega _{\mathcal {N}} = \omega , \qquad \sigma _t \circ \omega _{\mathcal {N}} = \omega _{\mathcal {N}} \circ \sigma _t. \end{aligned}$$

One can extend the conditional expectation to a positive contractive projection from $L^p (\mathcal {M})$ to $L^p (\mathcal {N})$ such that $\omega _{\mathcal {N}} (xyz) = x \omega _{\mathcal {N}} (y) z$ for any $x, z \in \mathcal {N}$ and $y \in L^p (\mathcal {M})$, see  [28]. This is a general result that can be applied to any completely positive contraction and is also relevant for proving hypercontractivity for suitable random variables. See  [29] for a general construction of such extensions and  [36] for its application to the proof of hypercontractivity within the *q*-deformed Araki–Woods factors, that is, algebras interpolating between the Araki–Wyss and the Araki–Woods ones as the deformation parameter *q* goes from $- 1$ to 1.^4^ For the sake of completeness, we report these results here. Let $P: \mathcal {M} \rightarrow \mathcal {N}$ be a completely positive contraction that is also state preserving (that is, $\omega \circ P = \omega$). Since $\mathcal {M}D^{\frac{1}{p}}$ is dense in $L^p$, one can introduce the densely-defined operators, for $p \in [1, \infty )$

2.8 $$\begin{aligned} P^{(p)}: \mathcal {M}D^{\frac{1}{p}} \rightarrow \mathcal {N}D^{\frac{1}{p}} \qquad xD^{\frac{1}{p}} \mapsto P (x) D^{\frac{1}{p}}. \end{aligned}$$

which extend to a contraction on $L^p (\mathcal {M})$, see  [29, Section 7] for the proof.

One can apply this result to the extension of the conditional expectation to Haagerup’s and to the twisted $L^p$ spaces. On $D^{\frac{1}{2 p} + \tau } \mathcal {M}_a D^{\frac{1}{2 p} - \tau }$ one can define

2.9 $$\begin{aligned} \omega _{\mathcal {N}}^{(p)} \left( {D^{\frac{1}{2 p} + \tau }} xD^{\frac{1}{2 p} - \tau } \right) = D^{\frac{1}{2 p} + \tau } \omega _{\mathcal {N}} (x) D^{\frac{1}{2 p} - \tau }. \end{aligned}$$

Since $\omega _{\mathcal {N}}$ maps $\mathcal {M}_a$ into the entire elements $\mathcal {N}_a$, which can be seen from (2.7), one sees that because of Lemma 2.2 and the state preserving property (2.7) $\omega ^{(p)}_{\mathcal {N}}$ does not depend on the parameter $\tau$, which was omitted from the notation on purpose. One can extend the map in (2.9) to the $L^p$ spaces and prove that such a map satisfies the usual properties  [28].

#### Proposition 2.23

The map $\omega _{\mathcal {N}}^{(p)}$ extends to a contractive projection, still denoted by $\omega ^{(p)}_{\mathcal {N}}$, from $L^p (\mathcal {M})$ to $L^p (\mathcal {N})$. Furthermore, the following properties hold true:

i. Let $p \in [1, \infty ]$. Then, $\forall x \in L^p (\mathcal {M})$ $(\omega _{\mathcal {N}}^{(p)} (x))^{*} = \omega _{\mathcal {N}}^{(p)} (x^{*})$ and $x \geqslant 0 \Rightarrow \omega _{\mathcal {N}}^{(p)} (x) \geqslant 0$.
ii. Let $p \in [2, \infty ]$. Then, $\forall x \in L^p (\mathcal {M})$ $(\omega _{\mathcal {N}}^{(p)} (x))^{*} \omega _{\mathcal {N}}^{(p)} (x) \leqslant \omega _{\mathcal {N}}^{(p)} (x^{*} x)$.
iii. Let $p, q, r \in [1, \infty ]$ such that $1 / p + 1 / q + 1 / r \leqslant 1$. Then, $\omega _{\mathcal {N}}^{(p)} (yxz) = y \omega _{\mathcal {N}}^{(p)} (x) z$ for any $x \in L^p (\mathcal {M})$, $y \in L^q (\mathcal {N})$ and $z \in L^r (\mathcal {N})$.

#### Remark 2.24

In particular, since $T^{(p)}_t (\omega _{\mathcal {N}} (x)) = \omega _{\mathcal {N}}^{(p)} (T^{(p)}_t (x))$ for $x \in \mathcal {M}_a$ this result allows us to extend the conditional expectation to the $\mathbb {L}^p$ spaces as well, extension which we shall denote by $\omega _{\mathcal {N}}$ with abuse of notation, that is, if $x \in \mathbb {L}^p$

$$\begin{aligned} \omega _{\mathcal {N}} (x) :=\mathbb {L}^p - \lim _n \omega _{\mathcal {N}} (x_n), \end{aligned}$$

for any $x_n \rightarrow x$ in the $\mathbb {L}^p$ topology. It follows that, see Remark 2.20,

$$\begin{aligned} T^{(p)}_t (\omega _{\mathcal {N}} (x)) = \omega _{\mathcal {N}}^{(p)} (T^{(p)}_t (x)) \qquad x \in \mathbb {L}^q, \, q \geqslant p. \end{aligned}$$

#### Remark 2.25

Note the following duality relation, for $x \in L^p (\mathcal {M})$ and $y \in L^q (\mathcal {M})$ with $1 / p + 1 / q = 1$

2.10 $$\begin{aligned} \operatorname {tr}_{\textrm{H}} (y \omega _{\mathcal {N}}^{(p)} (x)) = \operatorname {tr}_{\textrm{H}} (\omega _{\mathcal {N}}^{(q)} (y) x), \end{aligned}$$

which is a consequence of the tower property and the density of $\mathcal {M}_a D^{\frac{1}{p}}$ in $L^p (\mathcal {M})$. In fact, we have

$$\begin{aligned} \operatorname {tr}_{\textrm{H}} \left( D^{\frac{1}{q}} a \omega ^{(p)}_{\mathcal {N}} \left( bD^{\frac{1}{p}} \right) \right) = \omega (a \omega _{\mathcal {N}} (b)) = \omega (\omega _{\mathcal {N}} (a) b) = \operatorname {tr}_{\textrm{H}} \left( \omega ^{(q)}_{\mathcal {N}} \left( D^{\frac{1}{q}} a \right) bD^{\frac{1}{p}} \right) , \end{aligned}$$

from which (2.10) is obtained by continuity.

More generally, we will consider filtrations of vN subalgebras $(\mathcal {M}_t)_t$ invariant under the modular automorphism. In this case, it is useful to introduce the following nomenclature.

#### Definition 2.26

(Filtered modular space). The triple $(\mathcal {M}, \omega , (\mathcal {M}_t)_{t \in \mathbb {R}_+})$ consisting of a vNa $\mathcal {M}$, a modular state $\omega$ and a filtration of vNa’s $\mathcal {M}_t$ such that $\bigcup _{t \in \mathbb {R}_+} \mathcal {M}_t$ is $w^{*}$-dense in $\mathcal {M}$ is a filtered modular space if $\mathcal {M}_t$ are invariant under the modular group. In such a case, denote by $\omega _t: \mathcal {M} \rightarrow \mathcal {M}_t$ the conditional expectation and its extension to $L^p (\mathcal {M}_t)$ by $\omega ^{(p)}_t$.

In a filtered modular space, one can define adapted processes and martingales in the usual way. Of course, thanks to Proposition 2.23 this definition extends naturally to the $L^p$ and $\mathbb {L}^p$ spaces, the former including $\mathcal {M}$ as a special case.

#### Definition 2.27

Let $(\mathcal {M}, \omega , (\mathcal {M}_t)_{t \in \mathbb {R}_+})$ be a filtered modular space. We say that $(x_t)_t$ is an $L^p$ process if $x_t \in L^p (\mathcal {M})$, that is an adapted $L^p$ process if $x_t \in L^p (\mathcal {M}_t)$ for any $t \in \mathbb {R}_+$ and that it is an $L^p$ martingale if additionally $\omega ^{(p)}_s (x_t) = x_s$ for any $s \leqslant t$. An $L^p$ process is bounded if $\sup _{t \in \mathbb {R}_+} \Vert x_t \Vert _p < \infty$. An $L^p$ martingale is finite if there exists $t_0$ such that $x_t = x_{t_0}$ for any $t \geqslant t_0$.

#### Remark 2.28

The same definitions hold in the case of processes valued in $\mathcal {M}_a$ or in $\mathbb {L}^p$ with the obvious substitutions.

We can also introduce the notion of *independent-increment process*, which will play an important role in Sect. 3.

#### Definition 2.29

(Independent increment process) Let $X: \mathbb {R}_+ \times \mathfrak {h} \rightarrow \mathcal {M}$ be a stochastic process on $(\mathcal {M}, \omega , (\mathcal {M}_t)_t)$ indexed by $\mathfrak {h}$. We say that *X* has independent increments if for any $s \leqslant t \in \mathbb {R}_+$, for any $k \in \mathbb {N}$ and for any $f_1, \ldots , f_k \in \mathfrak {h}$ we have

$$\begin{aligned} \begin{array}{l} \omega _s ((X_t (f_1) - X_s (f_1)) \cdots (X_t (f_k) - X_s (f_k)))\\ \hspace{3em} = \omega ((X_t (f_1) - X_s (f_1)) \cdots (X_t (f_k) - X_s (f_k))). \end{array} \end{aligned}$$

One can also introduce the notion of independent subalgebras of $\mathcal {M}$.

#### Definition 2.30

Let $(\mathcal {M}, \omega )$ be a modular space and let $\mathcal {A}_1, \mathcal {A}_2 \subset \mathcal {M}$ be two subalgebras. We say that $\mathcal {A}_1, \mathcal {A}_2$ are two independent algebras if for any $a_1 \in \mathcal {A}_1, a_2 \in \mathcal {A}_2$ we have

2.11 $$\begin{aligned} \omega (a_1 a_2) = \omega (a_2 a_1) = \omega (a_1) \omega (a_2). \end{aligned}$$

#### Remark 2.31

A consequence of Definition 2.29 and the tower property for the conditional expectation, is that if the process $X_t$ has independent increments then, for any $s \leqslant t$, the algebra $\mathcal {M}_s$ and the algebra $\operatorname {span} \left\{ (X_t (f_1) - X_s (f_1)) \cdots (X_t (f_k) - X_s (f_k)),\right. \left. k \in \mathbb {N} \text { and } f_1, \ldots , f_k \in \mathfrak {h} \right\}$ are independent in the sense of Definition 2.30.

If $(t_n)_{n \geqslant 0} \subset \mathbb {R}_+$ is an unbounded set, having no accumulation point, such that $0 = t_0< \cdots < t_n$, with abuse of notation, we will denote by $(\mathcal {M}_n)_{n \in \mathbb {N}_0}$ the filtration of vNa’s $(\mathcal {M}_{t_n})_{n \in \mathbb {N}_0}$. We can then introduce *simple adapted processes* in $L^p$: *F* is an $L^p$ simple adapted process if there exists an unbounded set $(t_n)_{n \geqslant 0} \subset \mathbb {R}_+$ as before, such that

2.12 $$\begin{aligned} F_t = \sum _{n \geqslant 0} F_{t_n} \varvec{1}_{[t_n, t_{n + 1})} (t), \qquad F_{t_n} \in L^p (\mathcal {M}_n). \end{aligned}$$

Let us conclude this section by recalling the following fact about bounded $L^p$ martingales with respect to a discrete filtration $(\mathcal {M}_n)_{n \in \mathbb {N}_0}$.

#### Lemma 2.32

If $p \in (1, \infty )$ any bounded $L^p$ martingales $x = (x_n)_{n \in \mathbb {N}_0}$ has a limit $x_{\infty }$ in $L^p$ and $x_n = \omega _n (x_{\infty })$.

This is a general fact due to the uniform convexity of the $L^p$ spaces (Proposition 2.12), see, e.g., [46, Remark 1.3] for the tracial setting and  [28] for the non-tracial one. We provide a sketch of the proof for the reader’s convenience.

#### Proof

Since $x = (x_n)_n$ is bounded in $L^p$ it has a weakly convergent subsequence in $L^p$, $x_{n_k} \rightharpoonup X$, for which $x_n = \omega _n^{(p)} (X)$ holds for any $n \in \mathbb {N}$. Let us now assume that there is another subsequence $x_{m_k} \rightharpoonup Y$, with $Y \ne X$. Without loss of generality we may assume $\Vert X \Vert _p = 1$ and $\Vert Y \Vert _p \leqslant 1$. By uniform convexity, we have that there exists $\delta > 0$ such that $\Vert (X + Y) / 2 \Vert _p \leqslant 1 - \delta$. Then for all $n > 0$, by Proposition 2.23

$$\begin{aligned} \Vert x_n \Vert _p = \left\| \omega _n^{(p)} \left( \frac{X + Y}{2} \right) \right\| _p \leqslant \left\| \frac{X + Y}{2} \right\| _p \leqslant 1 - \delta . \end{aligned}$$

On the other hand, by duality we have weak lower semicontinuity

$$\begin{aligned} 1 = \Vert X \Vert _p \leqslant \lim \inf _k \Vert x_{n_k} \Vert _p \leqslant 1 - \delta \end{aligned}$$

which is a contradiction, therefore $X = Y$, that is, the weak limit is unique. We also have

$$\begin{aligned} \lim \sup _n \Vert x_n \Vert _p \leqslant \Vert X \Vert _p, \end{aligned}$$

and again by the weak lower semicontinuity $1 = \Vert X \Vert _p \leqslant \lim \inf _n \left\| \frac{X + x_n}{2} \right\| _p$. Thus, $\left\| \frac{X + x_n}{2} \right\| _p \rightarrow 1$ and by uniform convexity $\Vert x_n - X \Vert _p \rightarrow 0$. $\square$

#### Remark 2.33

Conversely, note that if $$x_{\infty } \in L^p$$, $$1 \leqslant p \leqslant \infty $$ then $$x = (x_n)_n$$ with $$x_n = \omega _n^{(p)} (x_{\infty })$$ defines a bounded $${L^p} $$ martingale and $$x_n \rightarrow x_{\infty }$$ in the $$L^p$$ topology. Thus, can identify a bounded $${L^p} $$ martingale with $$x_{\infty }$$ and viceversa.

### Martingale inequalities

Martingale inequalities play a crucial role in defining the Itô integral for a sufficiently large class of integrands, see Sect. 3. In the tracial setting, they have been established in  [46] and applied to the construction of the Itô–Clifford integral for suitable $L^p$ processes, extended the seminal result in $L^2$  [9]. Because non-trivial Grassmann martingales cannot be represented in the tracial setting, one has to resort to martingale inequalities in Haagerup’s $L^p$ spaces, which were established in  [28]. Below, we recall these results and adapt some of the details to the twisted $\mathbb {L}^p$ setting.

Let $(\mathcal {M}, \omega , (\mathcal {M}_n)_{n \in \mathbb {N}_0})$ be a filtered modular space, see Definition 2.26, and denote by $\omega _n^{(p)}$ the extension of the conditional expectation $\omega _n$ to the spaces $L^p (\mathcal {M})$. We shall now introduce some Hardy spaces of non-commutative martingales as follows. Consider finite sequences $x :=(x_n)_{n \geqslant 0} \subset L^p (\mathcal {M})$, with the topologies  [28, 46]

2.13 $$\begin{aligned}  &   \Vert x \Vert _{L^p (\mathcal {M}; \ell _c^2)} :=\left\| \left( \sum _{n \geqslant 0} | x_n |^2 \right) ^{1 / 2} \right\| _p, \quad \Vert x \Vert _{L^p_{\operatorname {cond}} (\mathcal {M}; \ell _c^2)} :=\left\| \left( \sum _{n \geqslant 0} \omega _{n - 1}^{\left( \frac{p}{2} \right) } (| x_n |^2) \right) ^{1 / 2} \right\| _p,\nonumber \\  &   \Vert x \Vert _{L^p (\mathcal {M}; \ell _r^2)} :=\left\| \left( \sum _{n \geqslant 0} | x_n^{*} |^2 \right) ^{1 / 2} \right\| _p, \quad \Vert x \Vert _{L^p_{\operatorname {cond}} (\mathcal {M}; \ell _r^2)} :=\left\| \left( \sum _{n \geqslant 0} \omega _{n - 1}^{\left( \frac{p}{2} \right) } (| x_n^{*} |^2) \right) ^{1 / 2} \right\| _p,\nonumber \\ \end{aligned}$$

with the identification $\omega _{- 1}^{(p)} = \omega _0^{(p)}$. To be precise, in the case of $L^p_{\operatorname {cond}} (\mathcal {M}; \ell _{\sharp }^2)$ for $1 \leqslant p < 2$ one should consider finite sequences in $\mathcal {M}_a D^{\frac{1}{p}}$, since the extension theorem for the conditional expectation does not hold for those values of *p*. The quantities in (2.13) define a norm on the space of finite sequences in $L^p (\mathcal {M})$ (or $\mathcal {M}_a D^{\frac{1}{p}}$ if needed) for any $p \geqslant 1$, see  [27, 28]. Additionally, consider the space $\ell ^p (L^p (\mathcal {M}))$ of sequences $x = (x_n)_n$ in $L^p (\mathcal {M})$ such that

$$\begin{aligned} \Vert x \Vert _{\ell ^p (L^p (\mathcal {M}))} :=\left( \sum _{n \geqslant 0} \Vert x_n \Vert ^p_p \right) ^{\frac{1}{p}} < \infty . \end{aligned}$$

Define the difference sequence $\delta x :=(\delta x_n)_{n \geqslant 0}$ with $\delta x_n :=x_n - x_{n - 1}$, where $x_{- 1} \equiv 0$. For any finite $L^p$ martingale introduce the norms

2.14 $$\begin{aligned} \begin{array}{l} \Vert x \Vert _{\mathcal {H}^p_{\sharp } (\mathcal {M})} :=\Vert \delta x \Vert _{L^p (\mathcal {M}, \ell ^2_{\sharp })}, \qquad \Vert x \Vert _{h^p_{\sharp }} :=\Vert \delta x \Vert _{L^p_{\operatorname {cond}} (\mathcal {M}, \ell ^2_{\sharp })}, \qquad \sharp = c, r,\\ \Vert x \Vert _{h^p_d} :=\Vert \delta x \Vert _{\ell ^p (L^p (\mathcal {M}))}. \end{array} \end{aligned}$$

The closure of the space of finite $L^p$ martingales under the norms in (2.14) are denoted respectively by $\mathcal {H}_{\sharp }^p (\mathcal {M})$, $h^p_{\sharp } (\mathcal {M})$ and $h^p_d (\mathcal {M})$. The Hardy spaces of non-commutative martingales are then defined as follows.

#### Definition 2.34

(Hardy spaces). For $p \in [1, 2)$ set

$$\begin{aligned} \mathcal {H}^p (\mathcal {M}) :=\mathcal {H}^p_c (\mathcal {M}) + \mathcal {H}^p_r (\mathcal {M}), \hspace{3em} h^p (\mathcal {M}) :=h^p_d (\mathcal {M}) + h^p_c (\mathcal {M}) + h^p_r (\mathcal {M}) \end{aligned}$$

with normsFor $p \in [2, \infty )$ set

$$\begin{aligned} \mathcal {H}^p (\mathcal {M}) :=\mathcal {H}^p_c (\mathcal {M}) \cap \mathcal {H}^p_r (\mathcal {M}), \hspace{3em} h^p (\mathcal {M}) :=h^p_d (\mathcal {M}) \cap h^p_c (\mathcal {M}) \cap h^p_r (\mathcal {M}) \end{aligned}$$

with norms

$$\begin{aligned} \Vert x \Vert _{\mathcal {H}^p (\mathcal {M})} :=\max _{\sharp = c, r} \Vert x \Vert _{\mathcal {H}^p_{\sharp } (\mathcal {M})}, \hspace{3em} \Vert x \Vert _{h^p (\mathcal {M})} :=\max _{\sharp = c, r, d} \Vert x \Vert _{h^p_{\sharp } (\mathcal {M})}. \end{aligned}$$

The next theorem extends the Burkholder–Gundy and Burkholder inequalities to the non-commutative setting of Haagerup’s $L^p$ spaces, see  [28].

#### Theorem 2.35

(Junge–Xu, 2003). Let $(\mathcal {M}, \omega , (\mathcal {M}_n)_n)$ be a filtered modular space. Let $1< p < \infty$ and let $x = (x_n)_{n \geqslant 0}$ be an $L^p$-martingale. Then, *x* is bounded iff $x \in \mathcal {H}^p (\mathcal {M})$ and iff $x \in h^p (\mathcal {M})$. In this case, there exist constants $\alpha _p, \beta _p, \delta _p, \eta _p > 1$ such that

2.15 $$\begin{aligned} \alpha _p^{- 1} \Vert x \Vert _{\mathcal {H}^p (\mathcal {M})} \leqslant \Vert x \Vert _p \leqslant \beta _p \Vert x \Vert _{\mathcal {H}^p (\mathcal {M})}, \end{aligned}$$

2.16 $$\begin{aligned} \delta _p^{- 1} \Vert x \Vert _{h^p (\mathcal {M})} \leqslant \Vert x \Vert _p \leqslant \eta _p \Vert x \Vert _{h^p (\mathcal {M})}, \end{aligned}$$

where the quantity in the middle identifies $x \equiv x_{\infty } \in L^p (\mathcal {M})$.

In particular, bearing in mind the identification $x \equiv x_{\infty }$ for bounded martingales, see Remark 2.33, one has the following corollary.

#### Corollary 2.36

For any $p \in (1, \infty )$ we have $L^p (\mathcal {M}) = \mathcal {H}^p (\mathcal {M}) = h^p (\mathcal {M})$ with equivalent norms.

Stein’s inequality  [28] is a further corollary of Theorem 2.35.

#### Corollary 2.37

Let $(\mathcal {M}, \omega , (\mathcal {M}_n)_n)$ be a filtered modular space and let $p \in (1, \infty )$. On finite sequences $x = (x_n)_n \subset L^p (\mathcal {M})$ define the map $Q (x) = (\omega ^{(p)}_n (x_n))_n$. Then, there exists a constant $\theta _p > 1$ such that

$$\begin{aligned} \Vert Q (x) \Vert _{L^p (\mathcal {M}, \ell ^2_{\sharp })} \leqslant \theta _p \Vert x \Vert _{L^p (\mathcal {M}, \ell ^2_{\sharp })}, \qquad \sharp = c, r \end{aligned}$$

that is *Q* extends to a bounded projection on $L^p (\mathcal {M}, \ell ^2_{\sharp })$ for $\sharp = c, r$.

For constructing the Itô integral, some further spaces of adapted processes are needed. We denote by $S_{\operatorname {ad}}^p$ the linear space of all $L^p$ simple adapted processes and by $\mathbb {S}_{\operatorname {ad}}$ the simple adapted processes taking values in $\mathcal {M}_a$. Following  [46], we introduce the following norms on $S_{\operatorname {ad}}^p$

2.17 $$\begin{aligned} \Vert F \Vert _{\mathcal {H}^p_c ([0, t])} :=\left\| \left( \int _0^t | F_s |^2 \textrm{d}s \right) ^{\frac{1}{2}} \right\| _p, \qquad \Vert F \Vert _{\mathcal {H}^p_r ([0, t])} :=\left\| \left( \int _0^t | F^{*}_s |^2 \textrm{d}s \right) ^{\frac{1}{2}} \right\| _p.\nonumber \\ \end{aligned}$$

From these norms, one can introduce Hardy spaces as was done in the discrete setting, see Definition 2.34 and details in  [46, Section 4]. For applications to the twisted setting, we consider instead the following twisted Hardy spaces.

#### Definition 2.38

For $\sharp = c, r$ let $\mathbb {H}^P_{\sharp } ([0, t])$ be the completion of $\mathbb {S}_{\operatorname {ad}}$ with respect to the twisted norms

$$\begin{aligned} \Vert F \Vert _{\mathbb {H}_{\sharp }^p ([0, t])} :=\sup _{| \tau | \leqslant 1 - \frac{1}{2 p}} \Vert T^{(p)}_{\tau } (F) \Vert _{\mathcal {H}^p_{\sharp } ([0, t])}. \end{aligned}$$

For $$p \in [2, \infty )$$ set

$$\begin{aligned} \mathbb {H}^p ([0, t]) :=\mathbb {H}_c^p ([0, t]) \cap \mathbb {H}_r^p ([0, t]), \qquad \Vert \cdot \Vert _{\mathbb {H}^p ([0, t])} :=\max _{\sharp = c, r} \Vert \cdot \Vert _{\mathbb {H}_{\sharp }^p ([0, t])}, \end{aligned}$$

and let $\mathbb {H}^p_{\operatorname {loc}} (\mathbb {R}_+)$ denote the space of processes on $\mathbb {R}_+$ whose restriction to [0, *t*] is in $\mathbb {H}^p ([0, t])$.

In the rest of this section, we prove the density of $\mathbb {S}_{\operatorname {ad}}$ in $\mathbb {H}^p$. Whereas such a property holds by construction in $\mathbb {H}^p_{\sharp }$ for $\sharp = c, r$, this is not a priori true for $\mathbb {H}^p$ because of the intersection topology. As shown in  [46] in the tracial setting, it is straightforward to project any element of $\mathbb {H}^p_{\operatorname {loc}} (\mathbb {R}_+)$ onto an approximating sequence in $\mathbb {S}_{\operatorname {ad}}$. To this end, one introduces the following bounded projections.

#### Lemma 2.39

Let $\sigma = (t_j)_{j \geqslant 0}$ be a subdivision of $\mathbb {R}_+$ with $t_0 = 0$. On $\mathbb {S}_{\operatorname {ad}}$ let

2.18 $$\begin{aligned} (Q_{\sigma } x) (t) :=\frac{1}{t_{k + 1} - t_k} \int _{t_k}^{t_{k + 1}} \omega _k (x (s)) \textrm{d}s, \qquad t_k \leqslant t < t_{k + 1}. \end{aligned}$$

Then, for any $p \in [2, \infty )$, $t \geqslant 0$ and $\sharp = c, r$ $Q_{\sigma }$ extends to a bounded projection on $\mathbb {H}_{\sharp }^p ([0, t])$.

#### Proof

The proof is an application of Stein’s inequality in Theorem 2.37, see  [46, Lemma 4.2] for details, and the following operator inequality for a family of bounded operators $(a_j)$ and the finite sequence $(r_j) \subset \mathbb {R}_+$, $\sum _j r_j = 1$

2.19 $$\begin{aligned} \left| \sum _j r_j a_j \right| ^2 \leqslant \sum _j r_j | a_j |^2, \end{aligned}$$

which is a consequence of convexity. The proof of  [46] for the tracial case carries over: for fixed $\tau$ and $x \in \mathbb {S}_{\operatorname {ad}}$ we have

$$\begin{aligned} \begin{array}{lll} \Vert T^{(p)}_{\tau } (Q_{\sigma } x) \Vert _{\mathcal {H}_c^p ([0, t])} &  = &  \left\| \left( \int _0^t | T^{(p)}_{\tau } (Q_{\sigma } x) |^2 \textrm{d}s \right) ^{\frac{1}{2}} \right\| _p\\ &  \leqslant &  \left\| \left( \sum _k \sum _{t_k \leqslant s_j< t_{k + 1}} (s_{j + 1} - s_j) | T^{(p)}_{\tau } (\omega _k (x (s_j))) |^2 \right) ^{\frac{1}{2}} \right\| _p\\ &  \leqslant &  \theta _p \left\| \left( \sum _k \sum _{t_k \leqslant s_j < t_{k + 1}} (s_{j + 1} - s_j) | T^{(p)}_{\tau } (x (s_j)) |^2 \right) ^{\frac{1}{2}} \right\| _p\\ &  = &  \theta _p \Vert T^{(p)}_{\tau } (x) \Vert _{\mathcal {H}_c^p ([0, t])}, \end{array} \end{aligned}$$

where the second line follows by (2.19) with $r_j = (s_{j + 1} - s_j) / (t_{k + 1} - t_k)$ and the definition of $Q_{\sigma }$, whereas the third line by Stein’s inequality. $\square$

One can also prove that, for any $x \in \mathbb {H}_{\sharp , \operatorname {loc}}^p (\mathbb {R}_+)$, by choosing $\sigma \rightarrow 0$, in the sense that $\sup _j (t_{j + 1} - t_j) \rightarrow 0$,

$$\begin{aligned} \lim _{\sigma \rightarrow 0} Q_{\sigma } x = x, \end{aligned}$$

$\operatorname {in} \mathbb {H}_{\sharp }^p ([0, t])$, for $\sharp = c, r$ and for any $t \geqslant 0$. In other words, one can produce an approximating sequence in $\mathbb {S}_{\operatorname {ad}}$ starting from $\mathbb {H}_{\sharp }^p ([0, t])$. If $x \in \mathbb {H}^p ([0, t])$ the approximating sequence converges in both $\{ \mathbb {H}_{\sharp }^p ([0, t]) \}_{\sharp = c, r}$ and thus in the $\mathbb {H}^p ([0, t])$ topology. We emphasize this result in the following corollary.

#### Corollary 2.40

For any $p \in [2, \infty )$ and $t \geqslant 0$ $\mathbb {S}_{\operatorname {ad}} ([0, t])$ is dense in $\mathbb {H}^p ([0, t])$.

##  Itô integration in the twisted $L^p$ spaces

Itô integration in the tracial setting has been constructed for Clifford random variables in  [9, 11, 12, 46]. In this section, we describe a general theory of integration with respect to $\mathbb {L}^p$ martingales under some mild assumptions on the martingale. Unlike the case of Clifford random variables, we deal with martingales whose square is not simply a multiple of the identity, property which was crucially used in the former works to compute the quadratic variation of Clifford martingales.

### Scalar Itô integral

We begin by considering the integration of an $\mathbb {L}^p$ simple adapted process against an $\mathbb {L}^q$ martingale.

#### Definition 3.1

(Itô integral). Let $q, p \in [1, \infty ]$ such that $1 / p + 1 / q \leqslant 1$. Let $F_t$ be a simple adapted process in $\mathbb {L}^p$ as defined in (2.12) and let $X_t$ be an $\mathbb {L}^q$ martingale; letting $X_{s, t} :=X_t - X_s$ for $s \leqslant t$, the Itô integral of *F* with respect to *X* is the process $Y = (Y_t)_t$

3.1 $$\begin{aligned} Y_t = \int _0^t F_s \cdot \textrm{d}X_s :=\sum _{j = 0}^k F_{t_j} \cdot X_{t_j, t_{j + 1} \wedge t}, \end{aligned}$$

where *k* is such that $t \in [t_k, t_{k + 1})$ and where the product between elements of the twisted spaces was given in Definition 2.17.

#### Remark 3.2

The Itô integral defines an $\mathbb {L}^r$ martingale, with $1 / r = 1 / p + 1 / q$.

In the rest of this section, we will extend the Itô integral to a larger class of adapted process. Note that in Definition 3.1 we could consider the integration of an $L^p$ simple adapted process against an $L^q$ martingale. However, one cannot extend the definition to more general $L^p$ adapted processes unless the martingale is analytic, stressing once more the relevance of the twisted $\mathbb {L}^p$ spaces.

As a warm up, we consider $\mathbb {L}^2$ valued processes and integration against a martingale in $\mathcal {M}_a$. We introduce the following subspace of $\mathbb {H}^p ([0, t])$, see Definition 2.38, for $p \in [2, \infty )$

3.2 $$\begin{aligned} \tilde{\mathbb {H}}^p ([0, t]) :=\left\{ ^{} F \in \mathbb {H}^p ([0, t]) \, | \, \int _0^t \Vert F_s \Vert _{\mathbb {L}^p}^2 \textrm{d}s < \infty \right\} . \end{aligned}$$

That this is indeed a subspace follows from the fact that for any $F \in \mathbb {S}_{\operatorname {ad}}$

$$\begin{aligned} \Vert F \Vert _{\mathbb {H}^p_{\sharp } ([0, t])}^2= &   \sup _{| \tau | \leqslant 1 - \frac{1}{2 p}} \Vert T_{\tau }^{(p)} (F) \Vert _{\mathcal {H}^p_{\sharp } ([0, t])}^2 = \sup _{| \tau | \leqslant 1 - \frac{1}{2 p}} \left\| \int _0^t | T_{\tau }^{(p)} (F_s)^{\sharp } |^2 \textrm{d}s \right\| _{\frac{p}{2}}\\\leqslant &   \int _0^t \Vert F_s \Vert _{\mathbb {L}^p}^2 \textrm{d}s, \end{aligned}$$

where we used the shorthand $T_{\tau }^{(p)} (F_s)^{\sharp }$ for $T_{\tau }^{(p)} (F_s)$ or $T_{\tau }^{(p)} (F_s)^{*}$ depending on whether $\sharp = c$ or $\sharp = r$.

#### Proposition 3.3

Let *F* be an $\mathbb {L}^2$ simple adapted process, let $X = (X_t)_t$ be an $\mathcal {M}_a$ martingale with independent increments, see Definition 2.29, such that, for any $0 \leqslant s \leqslant t$

3.3 $$\begin{aligned} \omega _s ([X_{s, t}^{*}]_r [X_{s, t}]_{r'}) = (t - s) c'_{r, r'}, \qquad \forall 0 \leqslant s \leqslant t, \qquad \forall r, r' \in \mathbb {R}, \end{aligned}$$

for some constant $c'_{r, r'}$. Let $Y_t$ denote the Itô integral as in Definition 3.1. Then, for any $| \tau | \leqslant \frac{3}{4}$ there is a constant $c_{\tau }$ (independent of *F*) such that

3.4 $$\begin{aligned} \Vert T^{(2)}_{\tau } (Y_t) \Vert _2^2 = c_{\tau } \int _0^t \Vert T^{(2)}_{\tau } (F_r) \Vert _2^2 \textrm{d}r. \end{aligned}$$

In particular, the Itô integral extends to a map from $\tilde{\mathbb {H}}^2 ([0, t])$ to $C^0 ([0, t] ; \mathbb {L}^2)$ and

$$\begin{aligned} \Vert Y_t \Vert ^2_{\mathbb {L}^2} \lesssim \int _0^t \Vert F_s \Vert _{\mathbb {L}^2}^2 \textrm{d}s. \end{aligned}$$

#### Remark 3.4

In Definition 4.13 will see examples of martingales that satisfy (3.3), compare with Lemma 4.15.

#### Proof

We fix $t \in \mathbb {R}_+$ and the set $(t_j)_{j \geqslant 0} \subset \mathbb {R}_+$, we let *k* be such that $t \in [t_k, t_{k + 1})$ and set $\tilde{t}_j = t_j \wedge t$ for $j = 0, \ldots , k$. We also abridge our notation by introducing $F_j \equiv F_{\tilde{t}_j}$ and $\delta X_j \equiv X_{\tilde{t}_{j + 1}} - X_{\tilde{t}_j}$. For $F_j \in \mathcal {M}_a \cap \mathcal {M}_j$, we have by the definition of Haagerup’s trace

$$\begin{aligned} \Vert T^{(2)}_{\tau } (Y_t) \Vert _2^2 = \operatorname {tr}_{\textrm{H}} (T^{(2)}_{- \tau } (Y_t^{*}) T^{(2)}_{\tau } (Y_t)) = \omega (Y_t^{*} [Y_t]_s), \end{aligned}$$

where we set $s = \frac{1}{2} + 2 \tau$. Then, by the modular property we can write

$$\begin{aligned} \Vert T^{(2)}_{\tau } (Y_t) \Vert _2^2 = \sum _{j, j'} \omega (\delta X_j^{*} F_j^{*} [F_{j'} \delta X_{j'}]_s) = \sum _{j, j'} \omega (F_j^{*} [F_{j'}]_s [\delta X_{j'}]_s [\delta X_j^{*}]_1). \end{aligned}$$

If $j' > j$, then we have

$$\begin{aligned} \omega (F_j^{*} [F_{j'}]_s [\delta X_{j'}]_s [\delta X_j^{*}]_1) = \omega (F_j^{*} [F_{j'}]_s \omega _{j'} ([\delta X_{j'}]_s) [\delta X_j^{*}]_1) = 0, \end{aligned}$$

since the conditional expectation is state preserving, see (2.7), and since *X* is an $\mathcal {M}_a$ martingale. Similarly, is the expectation vanishing if $j < j'$. Therefore, by assumption (3.3)

3.5 $$\begin{aligned} \Vert T^{(2)}_{\tau } (Y_t) \Vert _2^2 = \sum _j \omega (F_j^{*} [F_j]_s) \omega _j (\delta X_j^{*} [\delta X_j]_s) = c_{0, s}' \sum _j \Vert T^{(2)}_{\tau } (F_j) \Vert _2^2 \delta t_j, \end{aligned}$$

so that, by continuity, this holds true for any $\mathbb {L}^2$ valued simple adapted *F*, proving the claim (3.4). Since $c'_{0, s}$ is locally bounded, we can take the sup over $\tau$ we obtain $\Vert Y_t \Vert ^2_{\mathbb {L}^2} \lesssim \int _0^t \Vert F_s \Vert _{\mathbb {L}^2}^2 \textrm{d}s$ for any $F \in \mathbb {S}_{\operatorname {ad}}$. The conclusion follows by the density of $\mathbb {S}_{\operatorname {ad}}$ in $\tilde{\mathbb {H}}^2 ([0, t])$, see Lemma 2.40. $\square$

To extend this result to the general case of integration of an $\mathbb {L}^p$ adapted process against an $\mathbb {L}^q$ martingale, we shall now resort to martingale inequalities, see Theorem 2.35. The following is the main result of this section.

#### Theorem 3.5

Let $p, q, r \in (2, \infty ]$ with $1 / r \geqslant 1 / p + 1 / q$. Let $X = (X_t)_t$ be an $\mathbb {L}^q$ martingale with independent increments and such that

3.6 $$\begin{aligned} \Vert X_{s, t} \Vert _{\mathbb {L}^q} \lesssim (t - s)^{\frac{1}{2}}, \qquad \forall 0 \leqslant s \leqslant t . \end{aligned}$$

Then, the Itô integral extends to a map $F \mapsto Y_{\cdot } = \int _0^{\cdot } F_s \cdot \textrm{d}X_s$ from $\mathbb {H}_{\operatorname {loc}}^p (\mathbb {R}_+)$ to $C^0 (\mathbb {R}_+, \mathbb {L}^r)$ and

3.7 $$\begin{aligned} \Vert Y_t \Vert _{\mathbb {L}^r} \lesssim _r \Vert F \Vert _{\mathbb {H}^p ([0, t])}. \end{aligned}$$

#### Remark 3.6

Note once more that (3.6) is satisfied by the martingales introduced in Definition 4.13, see Lemma 4.15.

#### Proof

For the sake of brevity, we denote by $\mathcal {M}_j \equiv \mathcal {M}_{t_j}$ and write $F_j :=F_{t_j}$, $X_j :=X_{t_j}$, $\delta X_j :=X_{j + 1} - X_j$ and $\delta Y_j = F_{j - 1} \cdot \delta X_{j - 1}$ for $j \geqslant 1$ and $\delta Y_0 :=0$. The martingale property follows by direct inspection, since $F_j$ is adapted and $\omega ^{(q)}_{j'} \left( {\delta X_j} \right) = 0$, $\forall j' \leqslant j$, being $X_t$ a martingale.

To prove (3.7), we control the norm $\Vert T^{(r)}_{\tau } (Y_t) \Vert _r$ at fixed $\tau$ by means of Burkholder’s inequality (2.16). Thus, we shall bound the norms $\Vert \cdot \Vert _{h^r_c}$, $\Vert \cdot \Vert _{h^r_r}$ and $\Vert \cdot \Vert _{h^r_d}$. First of all, we consider $F_j, X_j \in \mathcal {M}_a \cap \mathcal {M}_j$ and note that

$$\begin{aligned} | T^{(r)}_{\tau } (\delta Y_{j + 1}) |^2 = D^{\frac{1}{2 r} - \tau } \delta X_j^{*} F^{*}_j [F_j \delta X_j]_{\frac{1}{r} + 2 \tau } D^{\frac{3}{2 r} + 2 \tau }. \end{aligned}$$

Thus, by definition of $\omega _j^{\left( \frac{r}{2} \right) }$ and by the properties of $\omega _j$

$$\begin{aligned} \begin{array}{lll} \omega _j^{\left( \frac{r}{2} \right) } (| T^{(r)}_{\tau } (\delta Y_{j + 1}) |^2) &  = &  {D^{\frac{1}{2 r} - \tau }} \omega _j \left( \delta X_j^{*} F^{*}_j [F_j \delta X_j]_{\frac{1}{r} + 2 \tau } \right) D^{\frac{3}{2 r} + \tau }\\ &  = &  {D^{\frac{1}{2 r} - \tau }} \omega _j \left( F^{*}_j [F_j \delta X_j]_{\frac{1}{r} + 2 \tau } [\delta X_j^{*}]_1 \right) D^{\frac{3}{2 r} + \tau }\\ &  = &  | T^{(r)}_{\tau } (F_j) |^2 \omega _j \left( \delta X_j^{*} [\delta X_j]_{\frac{1}{r} + 2 \tau } \right) , \end{array} \end{aligned}$$

where we used that $F_j, F_j^{*} \in \mathcal {M}_j$ and that $\omega _j \left( \delta X_j^{*} [\delta X_j]_{\frac{1}{r} + 2 \tau } \right) \in \mathbb {C}$, *X* having independent increments. The latter property also implies

$$\begin{aligned} \left\| \omega _j^{\left( \frac{r}{2} \right) } (| T^{(r)}_{\tau } (\delta X_j) |^2) \right\| _{\frac{r}{2}}= &   \left\| D^{\frac{1}{2 r} - \tau } \omega _j \left( \delta X_j^{*} [\delta X_j]_{\frac{1}{r} + 2 \tau } \right) D^{\frac{3}{2 r} + \tau } \right\| _{\frac{r}{2}}\\= &   \left| \omega _j \left( \delta X_j^{*} [\delta X_j]_{\frac{1}{r} + 2 \tau } \right) \right| . \end{aligned}$$

Accordingly, for any $| \tau | \leqslant 1 - \frac{1}{2 r}$, by Proposition 2.23 and by Hölder’s inequality we have, recalling that $r \leqslant q$

$$\begin{aligned} \left| \omega _j \left( \delta X_j^{*} [\delta X_j]_{\frac{1}{r} + 2 \tau } \right) \right| \leqslant \Vert | T^{(r)}_{\tau } (\delta X_j) | \Vert _r^2 \leqslant \Vert \delta X_j \Vert _{\mathbb {L}^q}^2. \end{aligned}$$

Since by Proposition 2.23$\omega _j^{\left( \frac{r}{2} \right) } (| T^{(r)}_{\tau } (\delta Y_{j + 1}) |^2) \geqslant 0$, we obtain

3.8 $$\begin{aligned} {\sum _{j \geqslant 0}} \omega _j^{\left( \frac{r}{2} \right) } (| T^{(r)}_{\tau } (\delta Y_{j + 1}) |^2) \leqslant \sum _{j \geqslant 0} | T^{(r)}_{\tau } (F_j) |^2 \Vert \delta X_j \Vert _{\mathbb {L}^q}^2, \end{aligned}$$

which can be extended by continuity to $X_j \in \mathbb {L}^q$. All in all, by assumption (3.6) we have

$$\begin{aligned} \sum _{j \geqslant 0} \omega _j^{\left( \frac{r}{2} \right) } (| T^{(r)}_{\tau } (\delta Y_{j + 1}) |^2) \lesssim \sum _{j \geqslant 0} | T^{(r)}_{\tau } (F_j) |^2 \delta t_j = D^{\frac{1}{q}} \left( \sum _{j \geqslant 0} \left| T^{(p)}_{\tau + \frac{1}{2 q}} (F_j ) \right| ^2 \delta t_j \right) D^{\frac{1}{q}} \end{aligned}$$

We observe that

$$\begin{aligned} \left( D^{\frac{1}{q}} \left( \sum _{j \geqslant 0} \left| T^{(p)}_{\tau + \frac{1}{2 q}} (F_j ) \right| ^2 \delta t_j \right) D^{\frac{1}{q}} \right) ^{\frac{1}{2}} = \left| \left( \sum _{j \geqslant 0} \left| T^{(p)}_{\tau + \frac{1}{2 q}} (F_j ) \right| ^2 \delta t_j \right) ^{\frac{1}{2}} D^{\frac{1}{q}} \right| , \end{aligned}$$

and thus, since $\Vert | x | \Vert _r = \Vert x \Vert _r$, by Hölder’s inequality we have

3.9 $$\begin{aligned} \begin{array}{lll} \Vert T^{(r)}_{\tau } (Y_t) \Vert _{h^r_c} &  \lesssim &  \left\| \left( D^{\frac{1}{q}} \left( \sum _{j \geqslant 0} \left| T^{(p)}_{\tau + \frac{1}{2 q}} (F_j ) \right| ^2 \delta t_j \right) D^{\frac{1}{q}} \right) ^{\frac{1}{2}} \right\| _r\\ &  =&  \left\| \left( \sum _{j \geqslant 0} \left| T^{(p)}_{\tau + \frac{1}{2 q}} (F_j ) \right| ^2 \delta t_j \right) ^{\frac{1}{2}} D^{\frac{1}{q}} \right\| _r\\ &  \leqslant &  \left\| \left( \sum _{j \geqslant 0} \left| T^{(p)}_{\tau + \frac{1}{2 q}} (F_j ) \right| ^2 \delta t_j \right) ^{\frac{1}{2}} \right\| _p. \end{array} \nonumber \\ \end{aligned}$$

We observe that if $| \tau | \leqslant 1 - \frac{1}{2 r}$, clearly $\left| \tau + \frac{1}{2 q} \right| \leqslant 1 - \frac{1}{2 r} + \frac{1}{2 q} = 1 - \frac{1}{2 p}$, hence

$$\begin{aligned} \sup _{| \tau | \leqslant 1 - \frac{1}{2 r}} \Vert T^{(r)}_{\tau } (Y_t) \Vert _{h^r_c} \lesssim \sup _{| \tau | \leqslant 1 - \frac{1}{2 p}} \left\| \left( \int _0^t | T^{(p)}_{\tau } (F_s ) |^2 \textrm{d}s \right) ^{\frac{1}{2}} \right\| _p \equiv \Vert F \Vert _{\mathbb {H}_c^p ([0, t])}, \end{aligned}$$

and similarly for the $\Vert \cdot \Vert _{h^r_r}$ norm. We control the diagonal Hardy norm by using Hölder’s inequality for the twisted spaces and the monotonicity of $x \mapsto x^{\frac{1}{r}}$ and $x \mapsto x^r$ for $x \in \mathbb {R}_+$,

$$\begin{aligned} \sup _{\tau } \left( \sum _j \Vert \delta Y_j \Vert ^r_r \right) ^{\frac{1}{r}}\leqslant &   \left( \sum _j (\sup _{\tau } \Vert \delta Y_j \Vert _r)^r \right) ^{\frac{1}{r}} = \left( \sum _j \Vert \delta Y_j \Vert _{\mathbb {L}^r}^r \right) ^{\frac{1}{r}}\\\lesssim &   \left( \sum _j \Vert F_j \Vert ^r_{\mathbb {L}^p} \delta t_j^{\frac{r}{2}} \right) ^{\frac{1}{r}}, \end{aligned}$$

which can be made as small as desirable for $r > 2$. Thus, the $h^p$ norm is dominated by the contribution $h^p_{\sharp }$ for $\sharp = c, r$, and the claim follows by Burkholder’s inequality

$$\begin{aligned} \Vert Y_t \Vert _{\mathbb {L}^r} \lesssim _p \max _{\sharp = c, r} \sup _{| \tau | \leqslant 1 - \frac{1}{2 r}} \Vert T^{(r)}_{\tau } (Y_t) \Vert _{h^r_{\sharp }} \lesssim \max _{\sharp = c, r} \Vert F \Vert _{\mathbb {H}_{\sharp }^p ([0, t])} = \Vert F \Vert _{{\mathbb {H}^p ([0, t])}}. \end{aligned}$$

By Lemma 2.40, $\mathbb {S}_{\operatorname {ad}}$ is dense in $\mathbb {H}^p$ hence (3.1) extends by continuity to any $F \in \mathbb {H}^p ([0, t])$. $\square$

#### Remark 3.7

It is possible to modify the hypotheses of Theorem 3.5, in such a way to consider processes *X* satisfying inequality (3.6) without having independent increments. Indeed, if we consider a martingale *X* such that $\Vert X_{s, t} \Vert _{\mathbb {L}^{\infty }} \lesssim (t - s)^{\frac{1}{2}}$ and $F \in \tilde{\mathbb {H}}^p_{\operatorname {loc}}$, for some $p \in (2, \infty ]$, see (3.2), then $Y_t = \int _0^t F_s \cdot \textrm{d}X_s$ is well-defined and, for every $r \in (2, p]$, $\Vert Y_t \Vert _{\mathbb {L}^r} \lesssim \Vert F \Vert _{\tilde{\mathbb {H}}^p ([0, t])}$. Under the described hypotheses, for any simple process $F \in \tilde{\mathbb {H}}^p_{\operatorname {loc}}$, we get

$$\begin{aligned} \left\| \sum _{j \geqslant 0} \omega _j^{\left( \frac{r}{2} \right) } (| T^{(r)}_{\tau } (\delta Y_{j + 1}) |^2) \right\| _{\mathbb {L}^r} \lesssim \sum _{j \geqslant 0} \Vert F_j \Vert _{\mathbb {L}^p} \delta t_j, \end{aligned}$$

where we used the fact that $\left\| \omega _j \left( \delta X_j^{*} [\delta X_j]_{\frac{1}{r} + 2 \tau } \right) \right\| _{L^{\infty }} \leqslant \Vert \delta X_j \Vert _{\mathbb {L}^{\infty }}^2 \lesssim \delta t_j$.

### Multidimensional Itô integral

We shall now consider martingales and adapted processes indexed by a Hilbert space $\mathfrak {h}$ with conjugation $\Theta$. We begin with the following definition.

#### Definition 3.8

Let $p \in [1, \infty ]$. We say that *F* is a simple adapted process in $\mathbb {L}^p$ indexed by the Hilbert space $\mathfrak {h}$ if $F : \mathbb {R}_+ \times \mathfrak {h} \rightarrow \mathbb {L}^p$ such that for any $t \in \mathbb {R}_+$, the map $v \mapsto F_t (v)$, $v \in \mathfrak {h}$, is linear, and for any $v \in \mathfrak {h}$, the process $t \mapsto F_t (v)$ is simple and adapted. Furthermore, we say that *F* is an $\mathbb {L}^p$ finite-dimensional simple adapted process, if furthermore there exists a set $\{ v_1, \ldots , v_k \} \subset \mathfrak {h}$ such that $F_t (v) = 0$ for any $v \in \operatorname {span} \{ v_1, \ldots , v_k \}^{\bot }$.

For finite-dimensional simple adapted processes the multidimensional Itô integral is the following generalisation of Definition 3.1.

#### Definition 3.9

Let $q, p \in [1, \infty ]$ such that $1 / p + 1 / q \leqslant 1$. Let *F* be an $\mathbb {L}^p$ finite-dimensional simple adapted process and $X = (X_t)_t$ an $\mathbb {L}^q$ martingale, both indexed by the Hilbert space $\mathfrak {h}$. The multidimensional Itô integral of *F* with respect to *X* is the process $Y = (Y_t)_t$

$$\begin{aligned} Y_t = \int _0^t \langle F_s, \textrm{d}X_s \rangle :=\sum _{\alpha } \int _0^t F_s (v_{\alpha }) \cdot \textrm{d}X_s (v_{\alpha }), \end{aligned}$$

where $\{ v_{\alpha } \}_{\alpha \in \mathbb {N}}$ is any orthonormal real basis wrt the conjugation $\Theta$ and where the r.h.s. is the scalar Itô integral of Definition 3.1.

#### Remark 3.10

Note that *Y* does not depend on the choice of the orthonormal real basis $\{ v_{\alpha } \}_{a \in \mathbb {N}}$.

#### Notation 3.11

Let $A: \mathfrak {h} \rightarrow \mathfrak {h}$ be a bounded linear operator and $F : \mathfrak {h} \rightarrow \mathbb {L}^p$, $H : \mathfrak {h} \rightarrow \mathbb {L}^q$, for some $q, p \geqslant 2$, be some linear (or anti-linear) maps. We set

$$\begin{aligned} \operatorname {Tr}_A (F \otimes H) :=\sum _{\alpha , \beta } \langle Av_{\alpha }, v_{\beta } \rangle _{\mathfrak {h}} F (v_{\alpha }) \cdot H (v_{\beta }) \end{aligned}$$

where $\{ v_{\alpha } \}_{\alpha \in \mathbb {N}}$ is some orthonormal real basis of $\mathfrak {h}$ wrt the conjugation $\Theta$, whenever the series is absolutely convergent in $\mathbb {L}^r$, $1 / r = 1 / p + 1 / q$, in which case it does not depend on the choice of the real orthonormal basis $\{ v_{\alpha } \}$.

#### Remark 3.12

When $A \geqslant 0$ commutes with $\Theta$, there is $\tilde{A}$ such that $\tilde{A}^2 = A$ and for any orthonormal (real) basis $\{ v_{\alpha } \}$ of h we have $\langle \tilde{A} v_{\alpha }, v_{\beta } \rangle _{\mathfrak {h}} = \langle \tilde{A} v_{\beta }, v_{\alpha } \rangle _{\mathfrak {h}} \in \mathbb {R}$ and as a series of positive operators

3.10 $$\begin{aligned} \operatorname {Tr}_A (F^{*} \otimes F) = \sum _{\alpha } | F (\tilde{A} (v_{\alpha })) |^2. \end{aligned}$$

For this reason it is appealing to introduce the notation

$$\begin{aligned} | F |_A^2 :=\operatorname {Tr}_A (F^{*} \otimes F). \end{aligned}$$

As long as *F* is finite-dimensional, the Itô integral can be extended as was done in the previous section to processes such that $F (v_{\alpha }) \in \mathbb {H}^p ([0, t])$ for $\alpha$ in a finite set. Otherwise, we introduce a finer topology in the following way. We let $\mathbb {S}_{\operatorname {ad}} (\mathfrak {h})$ denote the linear space of finite-dimensional simple adapted processes indexed by the Hilbert space $\mathfrak {h}$ and taking value in $\mathcal {M}_a$. On $\mathbb {S}_{\operatorname {ad}} (\mathfrak {h})$ we introduce the norms, compare with (2.17)

3.11 $$\begin{aligned} \Vert F \Vert _{\mathcal {H}^p_c ([0, t]); A} :=\left\| \left( \int _0^t | F_s |^2_A \textrm{d}s \right) ^{\frac{1}{2}} \right\| _p, \qquad \Vert F \Vert _{\mathcal {H}^p_r ([0, t]); A} :=\left\| \left( \int _0^t | F_s^{*} |^2_A \textrm{d}s \right) ^{\frac{1}{2}} \right\| _p.\nonumber \\ \end{aligned}$$

We then introduce the following extension of the twisted Hardy spaces of Definition 2.38.

#### Definition 3.13

Let $\mathfrak {h}$ be a separable Hilbert space with conjugation $\Theta$. Let $A \geqslant 0$ be a bounded linear operator on $\mathfrak {h}$ commuting with $\Theta$. For $\sharp = c, r$ let $\mathbb {H}^p_{A, \sharp } ([0, t])$ be the completion of $\mathbb {S}_{\operatorname {ad}} (\mathfrak {h})$ with respect to the twisted norms

$$\begin{aligned} \Vert F \Vert _{\mathbb {H}_{\sharp , A}^p ([0, t])} :=\sup _{| \tau | \leqslant 1 - \frac{1}{2 p}} \Vert T^{(p)}_{\tau } (F) \Vert _{\mathcal {H}^p_{\sharp } ([0, t]); A}. \end{aligned}$$

For $$p \in [2, \infty )$$ set

$$\begin{aligned} \mathbb {H}^p_A ([0, t]) :=\mathbb {H}_{A, c}^p ([0, t]) \cap \mathbb {H}_{A; r}^p ([0, t]), \qquad \Vert \cdot \Vert _{\mathbb {H}^p_A ([0, t])} :=\max _{\sharp = c, r} \Vert \cdot \Vert _{\mathbb {H}_{A, \sharp }^p ([0, t])}, \end{aligned}$$

and let $\mathbb {H}^p_{A, \operatorname {loc}} (\mathbb {R}_+)$ denote the space of processes on $\mathbb {R}_+$ whose restriction to [0, *t*] is in $\mathbb {H}^p_{{A}} ([0, t])$.

#### Remark 3.14

It is important to note that $\mathbb {S}_{\operatorname {ad}}$ is dense also in $\mathbb {H}^p_A$ and this is a simple generalisation of Corollary 2.40. In fact, it suffices to observe that (2.19), which was used in the proof of Stein’s inequality, holds for $| \cdot |_A$ as well: by (3.10), we have

$$\begin{aligned} \left| \sum _j r_j F_j \right| _A^2 = \sum _{\alpha } \left| \sum _j r_j F_j (\tilde{A} v_{\alpha }) \right| ^2 \leqslant \sum _{\alpha } \sum _j r_j | F_j (\tilde{A} v_{\alpha }) |^2 = \sum _j r_j | F_j |^2_A. \end{aligned}$$

We also introduce the following subspace of $\mathbb {H}_A^2 ([0, t])$, compare with (3.2)

$$\begin{aligned} \tilde{\mathbb {H}}_A^2 ([0, t]) :=\left\{ F \in \mathbb {H}_A^2 ([0, t]) | \int _0^t \Vert | F_s |_A \Vert _{\mathbb {L}^2}^2 \textrm{d}s < \infty \right\} . \end{aligned}$$

We consider integration of an $\mathbb {L}^p$ adapted process for $p \geqslant 2$ against a $\mathcal {M}_a$ martingale. In comparison with Proposition 3.3, note that we consider more general adapted processes, as was done in Theorem 3.5, albeit we still have some strong assumption on the martingale.

#### Theorem 3.15

Let $\mathfrak {h}$ be a separable Hilbert space with conjugation $\Theta$. Let $X = (X_t)_t$ be an $\mathcal {M}_a$ martingale indexed by $\mathfrak {h}$ such that

3.12 $$\begin{aligned} \omega ([X_{s, t}^{*} (f)]_r [X_{s, t} (g)]_{r'}) = (t - s) c'_{r, r'} \langle f, Ag \rangle _{\mathfrak {h}}, \qquad \forall 0 \leqslant s \leqslant t, \quad \forall f, g \in \mathfrak {h}, \quad \forall r, r' \in \mathbb {R},\nonumber \\ \end{aligned}$$

for some constant $c'_{r, r'}$ and some $A \geqslant 0$ commuting with $\Theta$. Then, the Itô integral extends to a map $F \mapsto Y_{\cdot } = \int _0^{\cdot } \langle F_s, \textrm{d}X_s \rangle$ from $\tilde{\mathbb {H}}_A^2 ([0, t])$ to $C^0 ([0, t] ; \mathbb {L}^2)$ and

$$\begin{aligned} \Vert Y_t \Vert ^2_{\mathbb {L}^2} \lesssim \int _0^t \Vert | F_s |_A \Vert _{\mathbb {L}^2}^2 \textrm{d}s. \end{aligned}$$

Assume furthermore that $\Vert [X_{s, t} (f)]_r \Vert \lesssim _{r, f} | t - s |^{1 / 2}$. Then, the Itô integral extends to a map $F \mapsto Y_{\cdot } = \int _0^{\cdot } \langle F_s, \textrm{d}X_s \rangle$ from $\mathbb {H}_{A, \operatorname {loc}}^p (\mathbb {R}_+)$ to $C^0 (\mathbb {R}_+ ; \mathbb {L}^p)$ for $p \in (2, \infty ]$ and

$$\begin{aligned} \Vert Y_t \Vert _{\mathbb {L}^p} \lesssim _p \Vert F \Vert _{\mathbb {H}^p_A ([0, t])}. \end{aligned}$$

#### Proof

In the case of $p = 2$ the proof of Proposition 3.3 carries over and one obtains

$$\begin{aligned}\begin{array}{lll} \Vert T^{(2)}_{\tau } (Y_t) \Vert _2^2 &  = &  \sum _j \sum _{\alpha , \beta } \omega (F_j^{*} (v_{\alpha }) [F_j (v_{\beta })]_s) \omega _j (\delta X_j^{*} (v_{\alpha }) [\delta X_j (v_{\beta })]_s)\\ &  = &  c_{0, s}' \sum _j \sum _{\alpha , \beta } \operatorname {tr}_{\textrm{H}} (T^{(2)}_{\tau } (F_j (v_{\alpha }))^{*} T^{(2)}_{\tau } (F_j (v_{\beta }))) \delta t_j \langle v_{\alpha }, Av_{\beta } \rangle _{\mathfrak {h}}\\ &  = &  c_{0, s}' \sum _j \Vert | T^{(2)}_{\tau } (F_j) |_A^2 \Vert \delta t_j, \end{array} \end{aligned}$$

in place of (3.5), hence the claim. The case $p \in (2, \infty ]$ is an adaptation of the proof of Theorem 3.5. Following the said proof, we obtain the identity

$$\begin{aligned} \begin{array}{lll} \omega _j^{\left( \frac{p}{2} \right) } (| T^{(p)}_{\tau } (\delta Y_{j + 1}) |^2) &  = &  \sum _{\alpha , \beta } T^{(p)}_{\tau } (F_j (v_{\alpha }))^{*} T^{(p)}_{\tau } (F_j (v_{\beta })) \omega _j \left( \delta X_j (v_{\alpha })^{*} [\delta X_j (v_{\beta })]_{\frac{1}{p} + 2 \tau } \right) \\ \, &  = &  c'_{0, \frac{1}{p} + 2 \tau } | T^{(p)}_{\tau } (F_j) |^2_A \delta t_j \end{array} \end{aligned}$$

compare with (3.8). Thus, instead of (3.9) we obtain the estimate

$$\begin{aligned} \Vert T^{(p)}_{\tau } (Y_t) \Vert _{h^p_c} \lesssim \left\| \left( \sum _{j \geqslant 0} | T^{(p)}_{\tau } (F_j ) |_A^2 \delta t_j \right) ^{\frac{1}{2}} \right\| _p, \end{aligned}$$

and likewise for the $\Vert \cdot \Vert _{h^p_r}$ norm. To control the diagonal Hardy norm, we use the additional assumption $\Vert [X_{s, t} (f)]_r \Vert \lesssim _{r, f} | t - s |^{1 / 2}$ and obtain

$$\begin{aligned} \sup _{\tau } \left( \sum _j \Vert \delta Y_j \Vert ^p_p \right) ^{\frac{1}{p}} \lesssim \left( \sum _j \left( \sum _{\alpha } C_{\alpha } \Vert F_j (v_{\alpha }) \Vert _{\mathbb {L}^p} \right) ^p \delta t_j^{\frac{p}{2}} \right) ^{\frac{1}{p}}, \end{aligned}$$

which for $p > 2$ and finite-dimensional processes can be made arbitrary small by making the partition of [0, *t*] finer. The conclusion holds by the density of $\mathbb {S}_{\operatorname {ad}}$ in $\mathbb {H}^p_A$, see Remark 3.14. $\square$

#### Remark 3.16

If one requires weaker assumptions, e.g.

3.13 $$\begin{aligned} \Vert X_{s, t} (f) \Vert _{\mathbb {L}^q} \lesssim (t - s)^{\frac{1}{2}} \Vert A^{1 / 2} f \Vert _{\mathfrak {h}}, \qquad \forall 0 \leqslant s \leqslant t, \quad \forall f \in \mathfrak {h}, \end{aligned}$$

then one can obtain the result of Theorem 3.15 provided that the topology on $\mathbb {S}_{\operatorname {ad}}^p$ is modified in a base-dependent way. Indeed, if we suppose that $F \in \mathbb {H}^p_A$ is such that there is a basis $\{ v_{\alpha } \}$ of $\mathfrak {h}$ for which, for any $t \in \mathbb {R}_+$ and for $F^{\#} = F, F^{*}$, we have

3.14 $$\begin{aligned} \left\| \left( \int _0^t \left| \sum _{\alpha } F_s^{\#} (v_{\alpha }) \Vert A^{1 / 2} v_{\alpha } \Vert _{\mathfrak {h}} \right| ^2 \textrm{d}s \right) ^{\frac{1}{2}} \right\| _{\mathbb {L}^r} < \infty , \end{aligned}$$

then the integral $Y_t = \int _0^t \langle F_s, \textrm{d}X_s \rangle$ is well-defined. Indeed, for any $F \in \mathbb {S}_{\operatorname {ad}}$, we get

$$\begin{aligned} \begin{array}{lll} \omega _j^{\left( \frac{r}{2} \right) } (| T^{(r)}_{\tau } (\delta Y_{j + 1}) |^2) &  = &  \sum _{\alpha , \beta } T^{(r)}_{\tau } (F_j (v_{\alpha }))^{*} T^{(r)}_{\tau } (F_j (v_{\beta })) \omega _j \left( \delta X_j (v_{\alpha })^{*} [\delta X_j (v_{\beta })]_{\frac{1}{r} + 2 \tau } \right) \\ \, &  \leqslant &  \delta t_j \left| \sum _{\alpha } T^{(r)}_{\tau } (F_j (v_{\alpha })) \Vert A^{1 / 2} v_{\alpha } \Vert _{\mathfrak {h}} \right| ^2, \end{array} \end{aligned}$$

from which one can conclude along the lines of the proof of Theorem 3.15.

## Grassmann random variables

### Araki–Wyss factors

While the tracial setting is not suitable for hosting non-trivial Grassmann martingales, see discussion in the Introduction, the Araki–Wyss factors  [6, 24] represent a convenient modular setting that serves our purposes. We introduce Araki–Wyss factors in the GNS representation associated with a quasi-free gauge invariant state characterised by an operator $0< \varrho < 1$.

#### Definition 4.1

(Araki–Wyss factors). Let $\mathcal {H}$ be a complex separable Hilbert space with conjugation $\Theta$ (that is, $\Theta$ is anti-unitary and $\Theta ^2 = \mathbb {1}$). Let $0< \varrho < 1$ be an operator acting on $\mathcal {H}$ such that $\Theta \varrho \Theta = \varrho$. Let $\Gamma _- (\mathcal {H} \oplus \mathcal {H})$ be the antisymmetric Fock space associated with $\mathcal {H} \oplus \mathcal {H}$ and let $a^{*}$, *a* denote the creation and annihilation operators on it. Set

$$\begin{aligned} \gamma _{\varrho } (f) :=a (\varrho f \oplus 0) + a^{*} (0 \oplus \varrho ^{- 1} \Theta f), \qquad f \in \mathcal {H}, \end{aligned}$$

and

$$\begin{aligned} \mathcal {A} (\mathcal {H}, \varrho ) :=\{ \gamma _{\varrho } (f) |f \in \mathcal {H} \}''. \end{aligned}$$

#### Remark 4.2

One could more generally introduce the *q*-deformed Araki–Woods factors by considering *q*-Fock spaces instead, but this is beyond the scope of this paper.

Note that this definition of $\mathcal {A} (\mathcal {H}, \varrho )$ makes the isomorphism with the Baby Fock models used in  [36, 41] evident, with the choice $I =\mathbb {Z}_0$, and $(\mu _j^{- 1})_{j \in \mathbb {N}}$ equal to the spectrum of $\varrho$. This is particularly convenient, as the results of  [36] on hypercontractivity carry over more easily, see Sect. 4.3.

We could equivalently work with the representation introduced by Shlyakhtenko  [50], see also  [23] for the *q*-deformation. The following isomorphism holds.

#### Proposition 4.3

In the same setting of Definition 4.1, $\mathcal {A} (\mathcal {H}, \varrho ) \cong \Gamma _{- 1} (\mathcal {H}_{\mathbb {R}}, (U^{(\varrho )}_t)_t)$, where $\Gamma _q$ is the *q*-deformed Araki–Woods factor introduced in  [23], where $\mathcal {H}_{\mathbb {R}}$ is the real Hilbert space such that $\mathcal {H}$ is its complexification and where $U^{(\varrho )}_t$ is the following orthogonal transformation

4.1 $$\begin{aligned} U_t^{(\varrho )} :=\left( \begin{array}{cc} \cos (t \log \varrho ^4) \varvec{1}_{\mathcal {H}_{\mathbb {R}}} &  - \sin (t \log \varrho ^4) \varvec{1}_{\mathcal {H}_{\mathbb {R}}}\\ \sin (t \log \varrho ^4) \varvec{1}_{\mathcal {H}_{\mathbb {R}}} &  \cos (t \log \varrho ^4) \varvec{1}_{\mathcal {H}_{\mathbb {R}}} \end{array} \right) . \end{aligned}$$

#### Remark 4.4

Note that orthogonal transformations of the type in (4.1) are already considered in  [23, 41].

The operators $\gamma _{\varrho }$’s satisfy the following anticommutation relations

4.2 $$\begin{aligned} \{ \gamma _{\varrho } (f), \gamma _{\varrho } (g) \} = 0, \qquad \{ \gamma _{\varrho }^{*} (f), \gamma _{\varrho } (g) \} = \langle g, (\varrho ^2 + \varrho ^{- 2}) f \rangle _{\mathcal {H}}, \qquad \forall f, g \in \mathcal {H},\nonumber \\ \end{aligned}$$

where $\{ \cdot , \cdot \}$ denotes the anticommutator. One can check that the Fock vacuum $\omega (\cdot ) :=\left\langle \Omega , \cdot \, \Omega \right\rangle$ is a gauge-invariant quasi-free state (see, e.g., [8, Example 5.2.18]) and that

4.3 $$\begin{aligned} \omega (\gamma _{\varrho }^{*} (f) \gamma _{\varrho } (g)) = \langle g, \varrho ^{- 2} f \rangle _{\mathcal {H}}, \hspace{3em} \omega (\gamma _{\varrho } (g) \gamma ^{*}_{\varrho } (f)) = \langle g, \varrho ^2 f \rangle _{\mathcal {H}}. \end{aligned}$$

For convenience, we set $\mathcal {K} :=\mathcal {H} \oplus \mathcal {H}$, let ${\hat{\Theta }}$ be the conjugation on $\mathcal {K}$ such that ${\hat{\Theta }} (f \oplus g) = \Theta g \oplus \Theta f$ and define the linear operators $\beta _{\varrho }: \mathcal {K} \rightarrow \mathcal {A} (\mathcal {H}, \varrho )$

4.4 $$\begin{aligned} \begin{array}{lll} \beta _{\varrho } (f \oplus f') &  :=&  \gamma _{\varrho }^{*} (\varrho ^{- 1} f) + \gamma _{\varrho } (\Theta \varrho f')\\ &  = &  a^{*} (f \oplus f') + a ((\varrho ^2 \oplus \varrho ^{- 2}) {\hat{\Theta }} (f \oplus f')), \end{array} \end{aligned}$$

which likewise generate $\mathcal {A} (\mathcal {H}, \varrho )$, that is, $\mathcal {A} (\mathcal {H}, \varrho ) = \{ \beta _{\varrho } (f) |f \in \mathcal {K} \}''$. For any $f, g \in \mathcal {K}$ we have

4.5 $$\begin{aligned} \omega (\beta _{\varrho } (f) \beta _{\varrho } (g)) = \langle {\hat{\Theta }} f, (\varrho ^2 \oplus \varrho ^{- 2}) g \rangle _{\mathcal {K}}, \quad \omega (\beta _{\varrho }^{*} (f) \beta _{\varrho } (g)) = \langle f, g \rangle _{\mathcal {K}}. \end{aligned}$$

We then introduce Wick’s polynomials.

#### Definition 4.5

Wick’s Polynomials in the $\beta _{\varrho }$’s are defined recursively by setting $\llbracket \beta _{\varrho } (f) \rrbracket :=\beta _{\varrho } (f)$ for any $f \in \mathcal {K}$ and for $f, f_1, \ldots f_n \in \mathcal {K}$

Furthermore, for any $F = f_1 \wedge \cdots \wedge f_n \in \mathcal {K}^{\wedge n}$, $n \in \mathbb {N}$, we set $\beta _{\varrho } (F) :=\beta _{\varrho } (f_1) \cdots \beta _{\varrho } (f_n)$ together with $a^{*} (F) :=a^{*} (f_1) \cdots a^{*} (f_n)$ and extend this by linearity to polynomials in $\Gamma _- (\mathcal {K})$. By straightforward computations, one can prove that for any *F* polynomial in $\Gamma _- (\mathcal {K})$

4.6 $$\begin{aligned} \llbracket \beta _{\varrho } (F) \rrbracket \Omega = a^{*} (F) \Omega , \end{aligned}$$

so that $\Omega$ is cyclic for $\mathcal {A} (\mathcal {H}, \varrho )$. As a simple consequence of (4.6), we can show that $\Omega$ is modular for $\mathcal {A} (\mathcal {H}, \varrho )$ and thus $(\mathcal {A} (\mathcal {H}, \varrho ), \omega )$ is in its GNS representation. To this end, one argues as follows. Consider the vNa

$$\begin{aligned} \tilde{\mathcal {A}} (\mathcal {H}, \varrho ) :=\{ a (\varrho ^{- 1} f \oplus 0) - a^{*} (0 \oplus \varrho \Theta f) |f \in \mathcal {H} \}''. \end{aligned}$$

Then, $\tilde{\mathcal {A}} (\mathcal {H}, \varrho )$ is in the commutant of $\mathcal {A} (\mathcal {H}, \varrho )$, that is, $\tilde{\mathcal {A}} (\mathcal {H}, \varrho ) \subset \mathcal {A} (\mathcal {H}, \varrho )'$. We have proved that $\Omega$ is cyclic for $\mathcal {A} (\mathcal {H}, \varrho )$ and likewise one can prove that $\Omega$ is cyclic for $\tilde{\mathcal {A}} (\mathcal {H}, \varrho )$. Accordingly, $\Omega$ is cyclic also for $\mathcal {A} (\mathcal {H}, \varrho )'$ and thus separating for $\mathcal {A} (\mathcal {H}, \varrho )$, that is, $\Omega$ is modular for the latter.

From (4.6) one can also deduce the identity

4.7 $$\begin{aligned} \omega (\llbracket \beta _{\varrho } (F) \rrbracket ^{*} \llbracket \beta _{\varrho } (G) \rrbracket ) = \langle F, G \rangle _{\Gamma _- (\mathcal {K})}. \end{aligned}$$

Since $\Omega$ is modular, one can compute the modular automorphism group and obtain the explicit formulas, compare with  [23]:

4.8 $$\begin{aligned} \Delta = \Gamma (\varrho ^{- 4} \oplus \varrho ^4), \quad \sigma _t (\beta _{\varrho } (f)) = \beta _{\varrho } ((\varrho ^{- 4 \textrm{i}t} \oplus \varrho ^{4 \textrm{i}t}) f), \quad \forall f \in \mathcal {K}, \end{aligned}$$

where for any bounded operator $B: \mathcal {K} \rightarrow \mathcal {K}$, $\Gamma (B)$ denotes the operator such that $\Gamma (B) f_1 \otimes \cdots \otimes f_n = Bf_1 \otimes \cdots \otimes Bf_n$. The simplest way to compute $\Delta$ is by inspection of (4.5): in fact since $\omega$ is modular we have

$$\begin{aligned} \langle (\varrho ^{- 2} \oplus \varrho ^2) {\hat{\Theta }} g, f \rangle _{\mathcal {K}}= &   \omega (\beta _{\varrho } (f) \beta _{\varrho } (g)) = \omega (\beta _{\varrho } (g) \sigma _{- \textrm{i}} (\beta _{\varrho } (f)))\\= &   \langle (\varrho ^2 \oplus \varrho ^{- 2}) {\hat{\Theta }} g, f' \rangle _{\mathcal {K}}, \end{aligned}$$

so that $f' = (\varrho ^{- 4} \oplus \varrho ^4) f$ and by the cyclic property obtain the expression for $\sigma _t (\beta _{\varrho } (f))$.

We conclude this section with the following classification result, which is a corollary of  [23, Theorem 3.1].

#### Corollary 4.6

Let *G* be the closed multiplicative subgroup of $\mathbb {R}_+$ generated by the spectrum of $\varrho$. Then, $\mathcal {A} (\mathcal {H}, \varrho )$ is a factor of type

$$\begin{aligned} \left\{ \begin{array}{lll} \textrm{I}\textrm{I}\textrm{I}_{\lambda } &  \quad \operatorname {if} \, &  G = \{ \lambda ^n |n \in \mathbb {Z}, \lambda \in (0, 1) \},\\ \textrm{I}\textrm{I}\textrm{I}_1 &  \quad \operatorname {if} \, &  G =\mathbb {R}_+. \end{array} \right. \end{aligned}$$

### Grassmann Brownian martingales

Let us recall the definition of Grassmann random variables, see  [1, 16] for more details.

#### Definition 4.7

(Grassmann random variable). Let $\mathcal {M}$ be a vNa and $\mathcal {H}$ a complex separable Hilbert space. A Grassmann random variable indexed by $\mathcal {H}$ is a linear map $\psi : \mathcal {H} \rightarrow \mathcal {M}$ such that

$$\begin{aligned} \{ \psi (f), \psi (g) \} = 0, \hspace{3em} \forall f, g \in \mathcal {H}. \end{aligned}$$

#### Remark 4.8

Denote by $\bigwedge \mathcal {H}$ the Grassmann algebra associated with the Hilbert space $\mathcal {H}$, that is, the quotient of the tensor algebra $\bigoplus _{n \geqslant 0} \mathcal {H}^{\otimes n}$ by the two-sided ideal generated by $\{ f \otimes f|f \in \mathcal {H} \}$. It is implied that a Grassmann random variable extends to an algebra homomorphism of the Grassmann algebra $\bigwedge \mathcal {H}$ into $\mathcal {M}$, by setting $\psi (f \wedge g) :=\psi (f) \psi (g)$. Equivalently, the set of Grassmann random variables on $\mathcal {M}$ indexed by $\mathcal {H}$ is identified with $\operatorname {Hom} \left( \bigwedge \mathcal {H} ; \mathcal {M} \right)$.

Given two Grassmann random variables $\psi _1$ and $\psi _2$, in general one has that $\{ \psi _1 (f), \psi _2 (g) \} \ne 0$, for some $f, g \in \mathcal {H}$, that is, the random variable $\zeta$ indexed by $\mathcal {H} \oplus \mathcal {H}$ and defined by $\zeta (f \oplus g) :=\psi _1 (f) + \psi _2 (g)$, is not a Grassmann random variable. If this is the case, we say that the Grassmann random variables $\psi _1$ and $\psi _2$ are compatible. The prototypical example of a Grassmann random variable not anticommuting with $\psi$ is the adjoint $\psi ^{*}$.

A process $\Psi _t$ is said to be Grassmann if $\Psi _t : \mathfrak {h} \rightarrow \mathcal {M}$ and if it satisfies anticommutation relations $\{ \Psi _t (f), \Psi _s (g) \} = 0$ for any *s*, *t* and any $\forall f, g \in \mathfrak {h}$. In a similar way, one defines Grassmann random variables and processes valued in the $\mathbb {L}^p$ spaces.

If $\Psi$ and $\Psi '$ are Grassmann random variables on different modular spaces, it is useful to be able to construct a modular space where $\Psi$ and $\Psi '$ are represented as independent and compatible Grassmann random variables.

#### Lemma 4.9

Let $(\mathcal {M}, \omega )$ and $(\mathcal {M}', \omega ')$ be two modular spaces and let $\Psi$ and $\Psi '$ be two Grassmann random variables taking value in the analytic elements $\mathcal {M}_a$ and $\mathcal {M}_a'$ respectively. Suppose further that the canonical representations $C, C'$ of $(\mathcal {M}, \omega )$ and $(\mathcal {M}', \omega ')$ respectively, admit some involutions $R, R'$, leaving some cyclic vectors invariant, anticommuting with $C (\Psi )$ and $C' (\Psi ')$. Then, it is always possible to build a modular space $(\mathcal {M}_{\operatorname {tot}}, \omega _{\operatorname {tot}})$ such that $\mathcal {M} \hookrightarrow \mathcal {M}_{\operatorname {tot}}$ and $\mathcal {M}' \hookrightarrow \mathcal {M}_{\operatorname {tot}}$, and $\omega _{\operatorname {tot}} |_{\mathcal {M}} = \omega$ and $\omega _{\operatorname {tot}} |_{\mathcal {M}'} = \omega '$. Furthermore, if we identify $\Psi$ and $\Psi '$ with their images in $\mathcal {M}_{\operatorname {tot}}$ under the embeddings mentioned above, then $\Psi$ and $\Psi '$ are independent compatible Grassmann random variables.

#### Proof

The proof can be found in  [1, Lemma 5] in the case of $C^{*}$-algebras. The case of modular spaces, discussed in the lemma, is a straightforward generalisation. $\square$

#### Remark 4.10

The hypotheses of Lemma 4.9 are satisfied by all Grassmann random variables indexed by a finite-dimensional (or more generally countably-generated) Hilbert space. These examples cover all the cases of Grassmann random variables considered in the rest of the paper.

An important class of Grassmann random variables are Grassmann Brownian martingales (GBM)  [1, 16].

#### Definition 4.11

Let $G = (G_t)_t$ a family of bounded and $\Theta$-antisymmetric operators on $\mathfrak {h}$. A Grassmann Brownian martingale on $(\mathcal {M}, \omega , (\mathcal {M}_t)_t)$ indexed by $\mathfrak {h}$ and with covariance *G* is the adapted centred Gaussian Grassmann process $X = (X_t)_t$ with independent increments, that is, $\omega _s ((X_t - X_s) (f)) = 0$ for any $s \leqslant t$ and $f \in \mathfrak {h}$, and such that

4.9 $$\begin{aligned} \omega (X_t (f) X_s (g)) = \langle \Theta f, G_{t \wedge s} g \rangle _{\mathfrak {h}} \hspace{3em} \forall s, t, \forall f, g \in \mathfrak {h}. \end{aligned}$$

We shall now show how non-trivial GBMs are constructed within Araki–Wyss factors. This is basically the Osterwalder–Schrader construction  [43] already implemented in  [1, 16], although in such cases a Fock state was used instead.

Let *h* be a complex separable Hilbert space, and let $$\Theta = \left( \begin{array}{cc} 0 &  \mathbb {1}\\ \mathbb {1} &  0 \end{array}\right) \kappa $$ be a conjugation on $\mathfrak {h} :=h \oplus h$, $\kappa$ denoting complex conjugation. We set

$$\begin{aligned} \mathcal {H} :=L^2 (\mathbb {R}_+; \mathfrak {h}), \qquad \mathcal {H}_t :=L^2 ([0, t]; \mathfrak {h}), \end{aligned}$$

and for $0< \mu < 1$ we introduce the vNa’s

$$\begin{aligned} \mathcal {M}^{(\mu )}_t :=\mathcal {A} (\mathcal {H}_t, \mu \mathbb {1}_{\mathcal {H}_t}) \subset \mathcal {M}^{(\mu )} :=\mathcal {A} (\mathcal {H}, \mu \mathbb {1}_{\mathcal {H}}). \end{aligned}$$

One can easily see that $(\mathcal {M}^{(\mu )}_t)_{t \geqslant 0}$ is a filtration of vN subalgebras of $\mathcal {M}^{(\mu )}$ invariant under the modular automorphism group, see (4.8), in the sense that $\sigma _s (\mathcal {M}_t^{(\mu )}) \subset \mathcal {M}_t^{(\mu )}$ for any $t \geqslant 0$ and $s \in \mathbb {R}$. Thus, there exists the conditional expectation $\omega _t : \mathcal {M}^{(\mu )} \rightarrow \mathcal {M}^{(\mu )}_t$, so that $(\mathcal {M}^{(\mu )}, \omega , (\mathcal {M}_t^{(\mu )})_{t \geqslant 0})$ is a filtered modular space, see Definition 2.26.

#### Lemma 4.12

Let $(G_t)_t$ be a differentiable family of bounded $\Theta$-antisymmetric operators on $\mathfrak {h}$. Let $\dot{G}_t = C^2_t U_t$ be the polar decomposition of $\dot{G}_t$, with $U_t$ unitary and let $0< \mu < 1$. Then, for any $t \geqslant 0$ the linear operators $\mathcal {C}_t, \tilde{\mathcal {C}}_t : \mathfrak {h} \rightarrow \mathcal {H}_t$ defined by

$$\begin{aligned} \mathcal {C}_t f :=(\mu ^{- 2} - \mu ^2)^{- \frac{1}{2}} \int _0^t \delta _s \otimes C_s U_s f \textrm{d}s, \quad \tilde{\mathcal {C}}_t f :=(\mu ^{- 2} - \mu ^2)^{- \frac{1}{2}} \int _0^t \delta _s \otimes C_s \Theta f \textrm{d}s, \end{aligned}$$

are bounded,

$$\begin{aligned} \Vert \mathcal {C}_t f \Vert _{\mathcal {H}}^2 \lesssim _{\mu } \int _0^t \Vert C_s U_s f \Vert _{\mathfrak {h}}^2 \textrm{d}s, \qquad \Vert \tilde{\mathcal {C}}_t f \Vert _{\mathcal {H}}^2 \lesssim _{\mu } \int _0^t \Vert C_s \Theta f \Vert _{\mathfrak {h}}^2 \textrm{d}s, \end{aligned}$$

and furthermore

$$\begin{aligned} \langle \tilde{\mathcal {C}}_s g, \mathcal {C}_t f \rangle _{\mathcal {H}} = - \langle \tilde{\mathcal {C}}_t f, \mathcal {C}_s g \rangle _{\mathcal {H}} = (\mu ^2 - \mu ^{- 2})^{- 1} \langle \Theta g, G_{s \wedge t} f \rangle _{\mathfrak {h}}. \end{aligned}$$

We abridge our notation to $\gamma \equiv \gamma _{\varrho = \mu \mathbb {1}_{\mathcal {H}}}$ and introduce GBM’s as follows.

#### Definition 4.13

Let $(\mathcal {M}^{(\mu )}, \omega , (\mathcal {M}_t^{(\mu )})_{t \geqslant 0})$ be as above and, let $(G_t)_t$ be a differentiable family of bounded $\Theta$-antisymmetric operators on $\mathfrak {h}$. Letting $\mathcal {C}_t$ and $\tilde{\mathcal {C}}_t$ be as in Lemma 4.12, we define the process $X_t: \mathfrak {h} \rightarrow \mathcal {M}_t^{(\mu )}$

4.10 $$\begin{aligned} X_t (f) :=\gamma ^{*} (\mathcal {C}_t f) - \gamma (\tilde{\mathcal {C}}_t f). \end{aligned}$$

#### Proposition 4.14

The process $X_t$ of Definition 4.13 is a GBM valued in $\mathcal {M}_a^{(\mu )} \cap \mathcal {M}^{(\mu )}_t$, with covariance $G_t$ and satisfies

4.11 $$\begin{aligned} \Vert X_{s, t} (f) \Vert ^2 \lesssim _{\mu } \int _s^t (\Vert C_r U_r f \Vert _{\mathfrak {h}}^2 + \Vert C_r \Theta f \Vert _{\mathfrak {h}}^2) \textrm{d}r. \end{aligned}$$

#### Proof

That *X* is adapted is clear by inspection. We check anticommutation relations by (4.2) and by Lemma 4.12

$$\begin{aligned} \begin{array}{lll} \{ X_t (f), X_s (g) \} &  = &  - (\{ \gamma ^{*} (\mathcal {C}_t f), \gamma (\tilde{\mathcal {C}}_s g) \} + \{ \gamma (\tilde{\mathcal {C}}_t f), \gamma ^{*} (\mathcal {C}_s g) \})\\ &  = &  - (\mu ^2 + \mu ^{- 2}) (\langle \tilde{\mathcal {C}}_s g, \mathcal {C}_t f \rangle _{\mathcal {H}} + \langle \tilde{\mathcal {C}}_t f, \mathcal {C}_s g \rangle _{\mathcal {H}}) = 0. \end{array} \end{aligned}$$

Furthermore, $X_t$ are Gaussian random variables because $\omega$ is quasi-free on $\gamma$’s. We compute the covariance with the aid of (4.3) and Lemma 4.12

$$\begin{aligned} \begin{array}{lll} \omega (X_t (f) X_s (g)) &  = &  - (\omega (\gamma ^{*} (\mathcal {C}_t f) \gamma (\tilde{\mathcal {C}}_s g) + \omega (\gamma (\tilde{\mathcal {C}}_t f) \gamma ^{*} (\mathcal {C}_s g))) \\ &  = &  - \mu ^{- 2} \langle \tilde{\mathcal {C}}_s g, \mathcal {C}_t f \rangle _{\mathcal {H}} - \mu ^2 \langle \tilde{\mathcal {C}}_t f, \mathcal {C}_s g \rangle _{\mathcal {H}}\\ &  = &  \langle \Theta g, G_{s \wedge t} f \rangle _{\mathfrak {h}} \end{array} \end{aligned}$$

Finally, (4.11) is obtained by Lemma 4.12 and by recalling that $\Vert a (f) \Vert , \Vert a^{*} (f) \Vert \leqslant \Vert f \Vert _{\mathcal {H}}$, for any $f \in \mathcal {H}$. $\square$

An important special case of GBM is the Grassmann Brownian motion, corresponding to the choice $G_t = tC^2 U$ with $U = \mathbb {1} \oplus - \mathbb {1}$ and some bounded $C \geqslant 0$. In Sect. 5, we will only consider this special case, while more general GBM’s will be used only in Sect. 6.1. In the former case, we note the following.

#### Lemma 4.15

If $X_t$ is as in Definition 4.13, then for any $f \in \mathfrak {h}$, $t \in \mathbb {R}_+$, $\tau \in \mathbb {R}$

4.12 $$\begin{aligned} [X_t (f)]_{\tau } = \mu ^{- 4 \tau } X_t (f). \end{aligned}$$

Assume furthermore that $G_t = tC^2 U$ with $[C, \Theta ] = [C, U] = 0$. Then, there exist constants $c_{r, r'}$ and $c_{r, r'}'$ depending on $\mu$ and locally bounded in $r, r' \in \mathbb {R}$ such that, for any $f, g \in \mathfrak {h}$, $t, s \in \mathbb {R}_+$, $r, r' \in \mathbb {R}$

4.13 $$\begin{aligned} \begin{array}{lll} \omega ([X_t (f)]_r [X_s (g)]_{r'}) &  = &  c_{r, r'} (s \wedge t) \langle \Theta f, Gg \rangle _{\mathfrak {h}},\\ \omega ([X_t^{*} (f)]_r [X_s (g)]_{r'}) &  = &  c_{r, r'}' (s \wedge t) \langle f, C^2 g \rangle _{\mathfrak {h}}. \end{array} \end{aligned}$$

Moreover, setting $X_{s, t} :=X_t - X_s$ we have $\Vert X_{s, t} (f) \Vert _{\mathbb {L}^p} \lesssim _{\mu , p} | t - s |^{\frac{1}{2}} \Vert Cf \Vert _{\mathfrak {h}}$ for any $p \in [1, \infty ]$.

#### Proof

The action of the automorphism group on $\beta \equiv \beta _{\varrho = \mu \mathbb {1}_{\mathcal {H}}}$, see (4.4) and (4.8) is simply $\sigma _t (\beta (f)) = \mu ^{- 4 \textrm{i}t} \beta (f)$, hence its analytical continuation is $[\beta (f)]_{\tau } = \mu ^{- 4 \tau } \beta (f)$. We can write $X_t (f) = \beta (f_t \oplus \tilde{f}_t)$, see Lemma 4.12, with $f_t :=\mu \mathcal {C}_t f$ and $\tilde{f}_t :=\mu ^{- 1} \tilde{\mathcal {C}}_t f$ so that (4.12) follows. This fact together with Proposition 4.14 implies the first identity in (4.13). The second identity follows by noting that $\mathcal {C}_t f \propto _{\mu } \varvec{1}_{[0, t]} \otimes UCf$ and $\tilde{\mathcal {C}}_t f \propto _{\mu } \varvec{1}_{[0, t]} \otimes \Theta Cf$, so that

$$\begin{aligned} \omega (X_t^{*} (f) X_s (g)) = \mu ^{- 2} \langle \tilde{\mathcal {C}}_s g, \tilde{\mathcal {C}}_t f \rangle _{\mathcal {H}} + \mu ^2 \langle \mathcal {C}_t f, \mathcal {C}_s g \rangle _{\mathcal {H}} = \frac{\mu ^2 + \mu ^{- 2}}{\mu ^{- 2} - \mu ^2} (s \wedge t) \langle f, C^2 g \rangle _{\mathfrak {h}}. \end{aligned}$$

Regarding the bound, by Hölder’s inequality and by (4.11) we have

$$\begin{aligned} \Vert T^{(p)}_{\tau } (X_{s, t} (f)) \Vert _p \leqslant \Vert [X_{s, t} (f)]_{\tau } \Vert \lesssim _{\mu , \tau } | t - s |^{\frac{1}{2}} \Vert Cf \Vert _{\mathfrak {h}}, \end{aligned}$$

implying the claim. $\square$

On the other hand, the following identity will be useful in Sect. 6.

#### Lemma 4.16

Let $X_t$ be as in Definition 4.13, then, for any $(f_j)_{j = 1}^n, (g_j)_{j = 1}^n \subset \mathfrak {h}$, $s, t \in \mathbb {R}_+$ and $\tau \in \mathbb {R}$

4.14 $$\begin{aligned}  &   \langle T^{(2)}_{\tau } (\llbracket X_t (f_1) \cdots X_t (f_n) \rrbracket ), T^{(2)}_{\tau } (\llbracket X_s (g_1) \cdots X_s (g_n) \rrbracket ) \rangle _{L^2}\nonumber \\  &   \qquad = \mu ^{- 2 (1 + 4 \tau ) n} \langle (f_{1, t} \oplus \tilde{f}_{1, t}) \wedge \cdots \wedge (f_{n, t} \oplus \tilde{f}_{n, t}), (g_{1, s} \oplus \tilde{g}_{1, s})\nonumber \\    &   \qquad \wedge \cdots \wedge (g_{n, s} \oplus \tilde{g}_{n, s}) \rangle _{\Gamma _- (\mathcal {K})},\nonumber \\ \end{aligned}$$

where $f_{j, t} :=\mu \mathcal {C}_t f_j$ and $\tilde{f}_{j, t} :=\mu ^{- 1} \tilde{\mathcal {C}}_t f_j$ and where $g_{j, s} :=\mu \mathcal {C}_s g_j$ and $\tilde{g}_{j, s} :=\mu ^{- 1} \tilde{\mathcal {C}}_s g_j$.

#### Proof

We note that

$$\begin{aligned} \begin{array}{lll} T^{(2)}_{\tau } (\llbracket X_t (f_1) \cdots X_t (f_n) \rrbracket ) &  = &  [\llbracket \beta (f_{1, t} \oplus \tilde{f}_{1, t}) \cdots \beta (f_{n, t} \oplus \tilde{f}_{n, t}) \rrbracket ]_{\frac{1}{4} + \tau } D^{\frac{1}{2}}\\ &  = &  \mu ^{- (1 + 4 \tau ) n} \llbracket \beta (f_{1, t} \oplus \tilde{f}_{1, t}) \cdots \beta (f_{n, t} \oplus \tilde{f}_{n, t}) \rrbracket D^{\frac{1}{2}} \end{array} \end{aligned}$$

and thus, by the definition of Haagerup’s trace and by (4.7) we obtain (4.14). $\square$

### Hypercontractivity

Let us now recall the hypercontractivity result in  [36] for the special case of Araki–Wyss factors. Let $P_t$ be the Ornstein–Uhlenbeck (OU) semigroup on $\mathcal {A} \equiv \mathcal {A} (\mathcal {H}, \varrho )$, defined by

$$\begin{aligned} P_t (x) \Omega :=\textrm{e}^{- t \mathcal {N}} (x \Omega ), \qquad \forall x \in \mathcal {A} (\mathcal {H}, \varrho ), \end{aligned}$$

where $\mathcal {N} = \textrm{d}\Gamma (\mathbb {1})$ is the number operator on $\Gamma _- (\mathcal {K})$. By (4.6), we have

4.15 $$\begin{aligned} P_t (\llbracket \beta _{\varrho } (f_1) \cdots \beta _{\varrho } (f_n) \rrbracket ) = \textrm{e}^{- tn} \llbracket \beta _{\varrho } (f_1) \cdots \beta _{\varrho } (f_n) \rrbracket , \end{aligned}$$

that is, Wick’s polynomials are eigenvectors of the OU semigroup. Furthermore, note that the modular operator $\Delta$ commutes with the number operator $\mathcal {N}$ and thus

4.16 $$\begin{aligned} \sigma _s (P_t (x)) = P_t (\sigma _s (x)), \qquad \forall s, t \in \mathbb {R}, \end{aligned}$$

so that $P_t$ maps the subalgebra of analytic elements $\mathcal {A}_a$ into itself. To extend this map to the $L^p$ spaces, one introduces the densely-defined operators, for $1 \leqslant p, q \leqslant \infty$,

4.17 $$\begin{aligned} P_t^{(p, q)}: \mathcal {A}D^{\frac{1}{p}} \rightarrow \mathcal {A}D^{\frac{1}{q}}, \qquad xD^{\frac{1}{p}} \mapsto P_t (x) D^{\frac{1}{q}}. \end{aligned}$$

These operators are not of the form (2.8), and in fact there is no general extension theorem that holds. Still, for suitable *p*, *q*, Lee–Ricard prove the following result  [36].

#### Theorem 4.17

(Lee–Ricard, 2011). Let $1< p < 2$. Then $P_t^{(p, 2)}$ extends to a contraction from $L^p (\mathcal {A} (\mathcal {H}, \varrho ))$ to $L^2 (\mathcal {A} (\mathcal {H}, \varrho ))$, that is, $\Vert P_t^{(p, 2)} \Vert _{L^p \rightarrow L^2} \leqslant 1$ provided that

$$\begin{aligned} \textrm{e}^{- 2 t} \lesssim \Vert \varrho \Vert _{\mathcal {H} \rightarrow \mathcal {H}}^{\frac{8}{p} - 4} (p - 1). \end{aligned}$$

#### Remark 4.18

Note that the map $P_t^{(p, q)}$ can be more generally defined on $D^{\frac{1}{2 p} + \tau } \mathcal {A}D^{\frac{1}{2 p} - \tau }$ by

4.18 $$\begin{aligned} P_t^{(p, q)} (T^{(p)}_{\tau } (x)) :=T^{(q)}_{\tau } (P_t (x)), \end{aligned}$$

compare with (2.9), and would not depend on $\tau$ because of Lemma 2.2 and the state preserving property (2.7). The statement of the theorem holds for any such map as well since in fact $T^{(p)}_t (x) = [x]_{\frac{1}{2 p} + t} D^{\frac{1}{p}}$ and by (4.16) we have $[P_t (x)]_s = P_t ([x]_s)$ for $x \in \mathcal {A}_a$, $P_t$ mapping $\mathcal {A}_a$ into itself.

#### Corollary 4.19

Let $2< p < \infty$. Then $P_t^{(2, p)}$ extends to a contraction from $L^2$ to $L^p$ provided that $\textrm{e}^{- 2 t} \lesssim \Vert \varrho \Vert _{\mathcal {H} \rightarrow \mathcal {H}}^{\frac{8}{p'} - 4} (p' - 1)$, with $1 / p' + 1 / p = 1$.

#### Proof

By duality and by the density of $\mathcal {M}_a D^{\frac{1}{p}}$, we can write, for $x \in \mathcal {M}_a$ and *t* as in the claim

$$\begin{aligned} \begin{aligned} \left\| P_t^{(2, p)} \left( xD^{\frac{1}{2}} \right) \right\| _p&= \sup _{{\begin{array}{l} y \in \mathcal {M}_a D^{1 / p'},\\ \Vert y \Vert _{p'} = 1 \end{array}}} \left| \operatorname {tr}_{\textrm{H}} \left( y^{*} P_t (x) D^{\frac{1}{p}} \right) \right| \\&= \sup _{{\begin{array}{l} y \in \mathcal {M}_a D^{1 / p'},\\ \Vert y \Vert _{p'} = 1 \end{array}}} \left| \operatorname {tr}_{\textrm{H}} \left( P_t^{(p', 2)} (y)^{*} xD^{\frac{1}{2}} \right) \right| \leqslant \left\| xD^{\frac{1}{2}} \right\| _2, \end{aligned} \end{aligned}$$

where the bound follows by Hölder’s inequality and by Theorem 4.17. This implies the claim. $\square$

The following hypercontractive estimates hold true.

#### Corollary 4.20

There exists a constant $C_{p, \varrho } \propto \Vert \varrho \Vert _{\mathcal {H} \rightarrow \mathcal {H}}^{2 - \frac{4}{p}} (p - 1)^{- \frac{1}{2}}$ such that, for any polynomial $F \in \Gamma _- (\mathcal {K})$

4.19 $$\begin{aligned} \Vert T^{(2)}_{\tau } (\llbracket \beta _{\varrho } (F) \rrbracket ) \Vert _2 \leqslant C_{p, \varrho }^{\deg (F)} \Vert T^{(p)}_{\tau } (\llbracket \beta _{\varrho } (F) \rrbracket ) \Vert _p \qquad p \in [1, 2), \end{aligned}$$

4.20 $$\begin{aligned} \Vert T^{(p)}_{\tau } (\llbracket \beta _{\varrho } (F) \rrbracket ) \Vert _p \leqslant C_{\frac{p}{p - 1}, \varrho }^{\deg (F)} \Vert T^{(2)}_{\tau } (\llbracket \beta _{\varrho } (F) \rrbracket ) \Vert _2 \qquad p \in (2, \infty ]. \end{aligned}$$

#### Proof

Take a monomial $F = f_1 \wedge \cdots \wedge f_n$. Then, by (4.15) and (4.18)

4.21 $$\begin{aligned} T^{(2)}_{\tau } (\llbracket \beta _{\varrho } (F) \rrbracket ) = \textrm{e}^{tn} T^{(2)}_{\tau } (P_t (\llbracket \beta _{\varrho } (F) \rrbracket )) = \textrm{e}^{tn} P_t^{(p, 2)} (T^{(p)}_{\tau } (\llbracket \beta _{\varrho } (F) \rrbracket )) \end{aligned}$$

and the estimate for $\Vert T^{(2)}_{\tau } (\llbracket \beta _{\varrho } (F) \rrbracket ) \Vert _2$ follows by Theorem 4.17, by choosing $\textrm{e}^t \sim \Vert \varrho \Vert _{\mathcal {H} \rightarrow \mathcal {H}}^{2 - \frac{4}{p}} (p - 1)^{- \frac{1}{2}}$. The estimate for $\Vert T^{(p)}_{\tau } (\llbracket \beta _{\varrho } (F) \rrbracket ) \Vert _p$ follows in the same way. To extend it to general polynomial $F = \sum _{n \geqslant 0} F_n$ with $F_n$ monomials, since $T^{(2)}_{\tau } (x) = [x]_{\frac{1}{4} + \tau } D^{\frac{1}{2}}$ by (4.7) we have

4.22 $$\begin{aligned} \Vert T^{(2)}_{\tau } (\llbracket \beta _{\varrho } (F) \rrbracket ) \Vert _2^2= &   \sum _{n, n'} \omega \left( [\llbracket \beta _{\varrho } (F_{n'}) \rrbracket ]_{\frac{1}{4} + \tau }^{*} [\llbracket \beta _{\varrho } (F_n) \rrbracket ]_{\frac{1}{4} + \tau } \right) \nonumber \\= &   \sum _n \left\| [\llbracket \beta _{\varrho } (F_n) \rrbracket ]_{\frac{1}{4} + \tau } \right\| _2^2, \end{aligned}$$

which allows one to obtain (4.19) from (4.21). The bound (4.20) follows by writing

$$\begin{aligned} \begin{array}{lll} \Vert T^{(p)}_{\tau } (\llbracket \beta _T (F) \rrbracket ) \Vert _p^2 &  = &  \left\| \sum _n \textrm{e}^{tn} P^{(2, p)} (T^{(2)}_{\tau } (\llbracket \beta _{\varrho } (F_n) \rrbracket )) \right\| ^2_p\\ &  \leqslant &  \left\| \sum _n \textrm{e}^{tn} T^{(2)}_{\tau } (\llbracket \beta _{\varrho } (F_n) \rrbracket ) \right\| ^2_2\\ &  = &  \sum _n \textrm{e}^{2 tn} \Vert T^{(2)}_{\tau } (\llbracket \beta _{\varrho } (F_n) \rrbracket ) \Vert ^2_2\\ &  \leqslant &  \left( C_{\frac{p}{p - 1}, \varrho }^{\deg (F)} \right) ^2 \Vert T^{(2)}_{\tau } (\llbracket \beta _{\varrho } (F) \rrbracket ) \Vert _2^2, \end{array} \end{aligned}$$

where in the first and second inequality we used Theorem 4.17 and (4.22). $\square$

## Grassmann stochastic calculus

In this section, we develop stochastic analysis of Itô–Grassmann processes, namely, we introduce stochastic integration for Grassmann-valued processes, prove Itô’s and Girsanov’s formula for such processes and finally provide a weak formulation of Grassmann SDEs.

### Itô–Grassmann integral

We begin by adapting Itô integration developed in Section to the special case of Grassmann Brownian martingales, allowing for a general definition Itô–Grassmann processes, for which a simple Itô formula can be proven.

To describe processes generated by some analytic GBM *X* and an additional independent compatible Grassmann random variable taking values in $\mathcal {M}_0$, see Definition 2.30 and Lemma 4.9, it is useful to introduce the following algebras.

#### Definition 5.1

Consider the filtered modular space $(\mathcal {M}, \omega , (\mathcal {M}_t)_t)$, let $X : \mathbb {R}_+ \times \mathfrak {h} \rightarrow \mathcal {M}_a$ be an analytic GBM, let $\tilde{X}_0 \in \operatorname {Hom} \left( \bigwedge \mathfrak {h} ; \mathcal {M}_0 \cap \mathcal {M}_a \right)$ be an independent and compatible Grassmann random variable, and set $\widetilde{\mathcal {M}}_0 :=\tilde{X}_0 \left( \bigwedge \mathfrak {h} \right)$. We denote by

$$\begin{aligned} \mathcal {G}_X :=\operatorname {span} \left\{ a_0 X_{t_1} (v_1) \cdots X_{t_n} (v_n), \text { where } a_0 \in \widetilde{\mathcal {M}}_0, \; n \in \mathbb {N}_0, \; v_1, \ldots , v_n \in \mathfrak {h}, \; t_1, \ldots , t_n \in \mathbb {R}^+ \right\} , \end{aligned}$$

the algebra of polynomials generated by *X* (and $\widetilde{\mathcal {M}}_0$). For every $p \in [1, \infty ]$ we let $\mathcal {G}^p_X$ be the closure of $\mathcal {G}_X$ with respect to the $\mathbb {L}^p$ topology.

#### Remark 5.2

The additional Grassmann random variable is typically needed to study SDE with non-trivial initial conditions, see Sects. 5.4 and 6.2.

In the following, it will be important to distinguish even and odd polynomials. To this end, recall that the Grassmann algebra $\bigwedge \mathfrak {h}$ has the superalgebra structure $\bigwedge \mathfrak {h} = \bigwedge ^+ \mathfrak {h} \oplus \bigwedge ^- \mathfrak {h}$, $\bigwedge ^+ \mathfrak {h}$ and $\bigwedge ^- \mathfrak {h}$ denoting the odd and even elements respectively. Accordingly, following Definition 5.1 , one can introduce the *even* and *odd algebras*$\mathcal {G}_{X, +}$ and$\mathcal {G}_{X, -}$ by restricting to $\bigwedge ^+ \mathfrak {h}$ and $\bigwedge ^- \mathfrak {h}$ respectively, and denote their closure in the $\mathbb {L}^p$ topology by $\mathcal {G}^p_{X, +}$and $\mathcal {G}^p_{X, -}$. Note that in general $\mathcal {G}^p_X = \overline{\operatorname {span} \{ \mathcal {G}^p_{X, +}, \mathcal {G}^p_{X, -} \}}$. However, if *X* is an analytic GBM as per Definition 4.13, with covariance $G = C^2 U$ such that *U* is unitary and $C > 0$, then, one can prove that $\mathcal {G}_{X, +}^p \cap \mathcal {G}_{X, -}^p = \{ 0 \}$ and thus we get that $\mathcal {G}_X^p$ is a superalgebra with decomposition $\mathcal {G}_X^p = \mathcal {G}_{X, +}^p \oplus \mathcal {G}_{X, -}^p$.

If an adapted process takes values in $\mathcal {G}^p_{X, +}$ or $\mathcal {G}^p_{X, -}$ for some $p \in [1, \infty ]$, we will henceforth say that it is an even or odd process respectively. In particular we have that, for any $v \in \mathfrak {h}$, *X*(*v*) is an odd process. If the simple adapted process in Definition 2.38 and 3.13 take value in either $\mathcal {G}_{X, +}$, $\mathcal {G}_{X, +}$ or $\mathcal {G}_{X, -}$, we can likewise introduce the Hardy–Grassmann spaces $\mathbb {G}_X^p$, $\mathbb {G}_{X, A}^p$, $\mathbb {G}^p_{X, \rho }$, $\mathbb {G}^p_{X, \rho , A}$, $\rho \mathop {=}\limits \pm$, in the same way in which the spaces $\mathbb {H}^p$, $\mathbb {H}^p_A$ have been defined.

We have the following straightforward but fundamental result.

#### Proposition 5.3

Let *X* be an analytic GBM with covariance $G_t = tG$ and let $F \in \mathbb {G}^p_{X, | G | ; \operatorname {loc}}$. Then, for any $t \in \mathbb {R}_+$, $\int _0^t \langle F_s, \textrm{d}X_s \rangle \in \mathcal {G}^p_X$. Furthermore if $F \in \mathbb {G}^p_{X, +, | G | ; \operatorname {loc}}$, then $\int _0^t \langle F_s, \textrm{d}X_s \rangle _ \in \mathcal {G}^p_{X, -}$ and if $F \in \mathbb {G}^p_{X, -, | G | ; \operatorname {loc}}$, then $\int _0^t \langle F_s, \textrm{d}X_s \rangle \in \mathcal {G}^p_{X, +}$.

#### Proof

The claim is obvious in the case where $F_s$ is a finite-dimensional simple process. The generic case can be obtained by the density of finite-dimensional simple processes in $\mathbb {H}^p_{| G |; \operatorname {loc}}$ and the continuity of the Itô integral in $\mathbb {L}^p$ (an thus in $\mathcal {G}^p_X$). $\square$

#### Remark 5.4

Unlike in the case of Clifford algebras [46, Theorem 4.6], we do not know if the converse of this result holds, namely if a Grassmann martingale $Y \in \mathcal {G}^{p}_{X}$ can be represented as an Itô–Grassmann integral with respect to *X* for a suitable integrand $F \in \mathbb {G}_{X,|G|;\textrm{loc}}$. The proof in the Clifford case relies significantly on the fact that the Clifford algebra is self-adjoint, that is, it is closed under the $*$ operation. This is not true for a Grassmann algebra, therefore $F^{*}$ might not belong to the Grassmann algebra.

We conclude this section by introducing the notion of Itô–Grassmann processes with respect to an analytic GBM, which, as shown in Lemma 4.15 satisfy the assumptions of Theorem 3.15.

#### Definition 5.5

(Itô–Grassmann process). Let $X = (X_t)_t$ be an analytic GBM with covariance $G_t = tG$. We say that $Y \in C^0 (\mathbb {R}_+ ; \mathcal {G}^p_X)$ is an Itô–Grassmann process (of integrability $p \geqslant 2$) if $Y_0 \in \mathcal {G}^p_X$, $H \in \mathbb {G}^p_{X, | G |, \operatorname {loc}}$, and $K : \mathbb {R}_+ \rightarrow \mathcal {G}^p_X$, $\Vert K \Vert _{\mathbb {L}^p} \in L^1_{\operatorname {loc}} (\mathbb {R}^+)$ such that

5.1 $$\begin{aligned} Y_t = Y_0 + \int _0^t \langle H_s, \textrm{d}X_s \rangle + \int _0^t K_s \textrm{d}s \end{aligned}$$

where the second integral is in the Bochner sense. If, in particular, $H_s \in \mathcal {G}^p_{X, +}$, $K_s \in \mathcal {G}^p_{X, -}$ (resp. $H_s \in \mathcal {G}^p_{X, -}$, $K_s \in \mathcal {G}^p_{X, +}$) we call *Y* an odd (resp. even) Itô–Grassmann process.

#### Remark 5.6

The above definition can be extended to the case of a multidimensional process *X* indexed by a (pre-)Hilbert space *W*. Indeed, we call the map $Y_{\cdot }: \mathbb {R}_+ \times W \rightarrow \mathcal {G}^p_X$ an Itô–Grassmann process indexed by *W* and of integrability $p \geqslant 2$, a map that for any $t \in \mathbb {R}_+$, $Y_t (\cdot )$ is a linear operator, for any $w \in W$, $Y_{\cdot } (w) \in C^0 (\mathbb {R}_+ ; \mathcal {G}^p_X)$, and there are $H : \mathbb {R}_+ \times W \times K \rightarrow \mathcal {G}^p_X$ and $K : \mathbb {R}_+ \times W \rightarrow \mathcal {G}^p_X$, which are linear in the *W* variable, and for any $w \in W$ we have $H_{\cdot } (w, \cdot ) \in \mathbb {G}^p_{X, | G | ; \operatorname {loc}}$, $\Vert K_{\cdot } (w) \Vert _{\mathbb {L}^p} \in L^1_{\operatorname {loc}} (\mathbb {R}_+)$, for which for any $t \in \mathbb {R}_+$ and $w \in W$ we have

5.2 $$\begin{aligned} Y_t (w) = Y_0 (w) + \int _0^t \langle H_s (w, \cdot ), \textrm{d}X_s \rangle + \int _0^t K_s (w) \textrm{d}s . \end{aligned}$$

If, for any $s \in \mathbb {R}_+$, $v \in \mathfrak {h}$ and $w \in W$, we have $F_s (w, v) \in \mathcal {G}^p_{X, +}$, $K_s (w) \in \mathcal {G}^p_{X, -}$ (resp. $F_s \in \mathcal {G}^p_{X, -}$, $K_s \in \mathcal {G}^p_{X, +}$) we call *Y* an odd (resp. even) Itô–Grassmann process.

#### Remark 5.7

The fact that $Y \in C^0 (\mathbb {R}_+; \mathcal {G}^p_X)$ or $Y (w) \in C^0 (\mathbb {R}_+ ; \mathcal {G}^p_X)$ follows directly from the representations (5.1) and (5.2). Furthermore, by Proposition 5.3, an odd/even Itô–Grassmann process is made (for fixed $s \in \mathbb {R}_+$ and $w \in W$) by odd/even elements of $\mathcal {G}^p_X$.

#### Remark 5.8

If $Y_t$ is an Itô process in $\mathbb {L}^p$ then, as consequence of Theorem 3.15, for every $T > 0$, we have

$$\begin{aligned} \lim _{\pi \in \left\{ \text {partitions of } [0, T] \right\} , | \pi | \rightarrow 0} \left( \sup _{t_i \in \pi } \quad \sup _{t \in [t_i, t_{i - 1}]} \Vert Y_{t_{i - 1}} - Y_s \Vert _{\mathbb {L}^{2 p}} \right) = 0 \end{aligned}$$

where the limit is taken with respect to the diameter $| \pi |$ of partitions $\pi$ going to 0. This property is analogous to the uniform continuity in $L^p$ for commutative stochastic processes.

### Itô formula

In this section we want to prove an Itô formula for Itô–Grassmann processes in the sense of Definition 5.5. We remark that the following discussion could be extended to the anticommutative analogue of continuous commutative martingales - see, e.g., [47, Chapter IV, Section 5] for the latter notion.

#### Definition 5.9

Let $p, q \in [1, \infty ]$ and let $B, B'$ be two martingales in $C^0 (\mathbb {R}_+ ; \mathbb {L}^p)$ and $C^0 (\mathbb {R}_+ ; \mathbb {L}^q)$ respectively. The quadratic variation $[B, B'] \in C^0 (\mathbb {R}_+ ; \mathbb {L}^r)$ with $1 / r = 1 / p + 1 / q$ is a process of bounded variation (in the $\mathbb {L}^r$ norm) such that the process

$$\begin{aligned} t \mapsto B_t \cdot B'_t - [B, B']_t \end{aligned}$$

is an $\mathbb {L}^r$ martingale.

#### Lemma 5.10

Let $X = (X_t)_t$ be an analytic GBM with covariance $G_t = tG$. Then, for any $v, v' \in \mathfrak {h}$ we have

$$\begin{aligned} [X (v), X (v')]_t = \langle \Theta v, G v' \rangle _{\mathfrak {h}} t. \end{aligned}$$

#### Proof

The proof is a consequence of the fact that the processes $X_t (v)$ and $X_t (v')$ have independent increments and that $\omega ((X_{s, t}) (v) (X_{s, t}) (v')) = \omega _s ((X_{s, t}) (v) (X_{s, t}) (v')) = \langle \Theta v, G v' \rangle (t - s)$, for any $0 \leqslant s \leqslant t$. $\square$

#### Theorem 5.11

Let $Y = (Y_t)_t, Y' = (Y'_t)_t$ be two Itô–Grassmann processes of integrability $p \geqslant 2$ of the form

$$\begin{aligned} Y_t = Y_0 + \int _0^t \langle H_s, \textrm{d}X_s \rangle , \quad Y'_t = Y_0' + \int _0^t \langle H_s', \textrm{d}X_s \rangle , \end{aligned}$$

where $X = (X_t)_t$ is an analytic GBM indexed by $\mathfrak {h}$with covariance $G_t = tG$. Then, we have

5.3 $$\begin{aligned} [Y, Y']_t = \int _0^t \operatorname {Tr}_{G^{*} \Theta } (H_s \otimes H'_s) \textrm{d}s, \end{aligned}$$

where $\operatorname {Tr}_{G^{*} \Theta } (\cdot )$ is defined in Notation 3.11, $\Theta$ being the conjugation in $\mathfrak {h}$.

#### Proof

We prove the theorem in the special case of $Y, Y'$ being simple finite-dimensional processes. The general case follows from the linearity of the quadratic variation and by density. On the other hand, formula (5.3) follows directly from the definition of simple processes, the properties of conditional expectation and Lemma 5.10. $\square$

For convenience, we introduce some subspaces of the spaces $\mathbb {H}^p_A$, see Definition 3.13.

#### Definition 5.12

Let *F* an $\mathbb {L}^p$ adapted process indexed by a Hilbert space $\mathfrak {h}$ with conjugation $\Theta$ and let $A \geqslant 0$ be a linear bounded operator on $\mathfrak {h}$ commuting with $\Theta$. We let

5.4 $$\begin{aligned} \Vert F_s \Vert _{\mathbb {L}^p, A} :=\Vert | F_s |_A \Vert _{\mathbb {L}^p}, \end{aligned}$$

and introduce the following subspace of $\mathbb {H}^p_{A, \operatorname {loc}}$

$$\begin{aligned} \tilde{\mathbb {H}}^p_{A, \operatorname {loc}} :=\left\{ ^{} F \in \mathbb {H}^p_{A, \operatorname {loc}} \, | \, \Vert F_s \Vert _{\mathbb {L}^p, A} \in L^2_{\operatorname {loc}} (\mathbb {R}_+) \right\} . \end{aligned}$$

#### Remark 5.13

One can check that $\tilde{\mathbb {H}}^p_{A, \operatorname {loc}}$ is indeed a subspace of $\mathbb {H}^p_{A, \operatorname {loc}}$, in fact

$$\begin{aligned} \Vert F \Vert _{\mathbb {H}^p_A ([0, t])} \lesssim \left( \int _0^t \Vert F_s \Vert ^2_{\mathbb {L}^p, A} \textrm{d}s \right) ^{\frac{1}{2}}. \end{aligned}$$

We also note that $\tilde{\mathbb {H}}^p_{A, \operatorname {loc}}$ are semimetric spaces with the set of semidistances

$$\begin{aligned} d_t (F, G) \mapsto \left( \int _0^t \Vert F_s - G_s \Vert _{\mathbb {L}^p, A}^2 \textrm{d}s \right) ^{\frac{1}{2}}. \end{aligned}$$

Furthermore the finite-dimensional processes taking values in $\mathbb {L}^p$ defined in Definition 3.9 are dense in $\tilde{\mathbb {H}}^p_{A, \operatorname {loc}}$ with respect to its natural topology, compare with Remark 3.14.

#### Corollary 5.14

Under the hypotheses of Theorem 5.11, the following inequality holds

5.5 $$\begin{aligned} \int _0^t \Vert \operatorname {Tr}_{G^{*} \Theta } (H_s \otimes H'_s) \Vert _{\mathbb {L}^r} \textrm{d}s \lesssim \Vert H \Vert _{\tilde{\mathbb {H}}^p_{| G |} ([0, t])}^2 + \Vert H' \Vert _{\tilde{\mathbb {H}}^q_{| G |} ([0, t])}^2. \end{aligned}$$

#### Proof

We will use the representation (5.3). Consider first $H, H' \in \mathbb {S}_{\operatorname {ad}}$ and let $[s, t] \subset \mathbb {R}_+$ be an interval where both *H* and $H'$ are constant so that $H_r \cdot H_r' \in \mathcal {M}_a \cap \mathcal {M}_s$ for $r \in [s, t]$ and thus

$$\begin{aligned} {\int _s^t \Vert \operatorname {Tr}_{G^{*} \Theta } (H_r \otimes H_r') \Vert _{\mathbb {L}^r} \textrm{d}r = \left\| \omega _s \left( \int _s^t \operatorname {Tr}_{G^{*} \Theta } (H_r \otimes H_r') \textrm{d}r \right) \right\| _{\mathbb {L}^r}.} \end{aligned}$$

Then, by Theorem 5.11 and by using that $t \mapsto Y_t \cdot Y_t' - [Y, Y']_t$ is a martingale

$$\begin{aligned} \begin{array}{lll} \omega _s \left( \int _s^t \operatorname {Tr}_{G^{*} \Theta } (H_r \otimes H_r') \textrm{d}r \right) &  = &  \omega _s ([Y, Y']_t - [Y, Y']_s)\\ &  = &  \omega _s \left( \int _s^t \langle H_r, \textrm{d}X_r \rangle \right) \left( \int _s^t \langle H_{r'}', \textrm{d}X_{r'} \rangle \right) . \end{array} \end{aligned}$$

By Hölder’s inequality, Theorem 3.15 and the bound $\Vert \cdot \Vert _{\mathbb {H}^p_A ([0, t])}^2 \leqslant \Vert \cdot \Vert ^2_{\tilde{\mathbb {H}}^p_A ([0, t])}$ we thus have

$$\begin{aligned} \begin{array}{lll} \int _s^t \Vert \operatorname {Tr}_{G^{*} \Theta } (H_r \otimes H_r') \Vert _{\mathbb {L}^r} \textrm{d}r &  \leqslant &  \left\| \left( \int _s^t \langle H_r, \textrm{d}X_r \rangle \right) \left( \int _s^t \langle H_{r'}', \textrm{d}X_{r'} \rangle \right) \right\| _{\mathbb {L}^r}\\ &  \lesssim &  \left( \int _s^t \Vert H_r \Vert ^2_{\mathbb {L}^p, | G |} \textrm{d}r \right) + \left( \int _s^t \Vert H_s' \Vert ^2_{\mathbb {L}^q, | G |} \textrm{d}r \right) , \end{array} \end{aligned}$$

Taking the sum over a partition where both $H_s$ and $H'_s$ are constant, we obtain (5.5). $\square$

We introduce the following standard notation.

#### Notation 5.15

If $Y, Y'$ are Itô–Grassmann processes of the form $Y_t = Y_0 + \int _0^t \left\langle {H_s} , \textrm{d}X_s \right\rangle + \int _0^t K_s \textrm{d}s$, $Y_t' = Y_0' + \int _0^t \left\langle {H_s'} , \textrm{d}X_s \right\rangle + \int _0^t K_s' \textrm{d}s$, and $\tilde{H}$ and $\tilde{K}$ are adapted processes, we use the notation

$$\begin{aligned} \int _0^t \tilde{H}_s \textrm{d}Y_s  &   :=\int _0^t \langle \tilde{H}_s H_s, \textrm{d}X_s \rangle + \int _0^t \tilde{H}_s K_s \textrm{d}s, \\ \int _0^t \tilde{K}_s \textrm{d}[Y, Y']_s  &   :=\int _0^t \tilde{K}_s \operatorname {Tr}_{G^{*} \Theta } (H_s \otimes H'_s) \textrm{d}s. \end{aligned}$$

We want to prove an Itô formula for Itô–Grassmann processes indexed by some finite-dimensional linear space *W*. To this end, we introduce the space of polynomials of degree at most $k \in \mathbb {N}$ of commutative and anticommutative random variables indexed by *W*:

$$\begin{aligned} \mathcal {F}^k (W) = \bigoplus _{n = 0}^k \bigoplus _{\ell = 0}^n \Lambda ^{n - \ell } W \otimes \odot ^{\ell } W, \end{aligned}$$

i.e., the tensor product of the antisymmetric and symmetric vector spaces generated by *W*. We also use the notation $\mathcal {F}^{\infty } (W) = \bigcup _{k \in \mathbb {N}} \mathcal {F}^k (W)$. The space $\mathcal {F}^{\infty } (W)$ is an algebra with the following multiplication: if $F \in \mathcal {F}^k (W)$, that is,

5.6 $$\begin{aligned} F = \sum _{n = 0}^k \sum _{\ell = 0}^n F^{(n, \ell )} \otimes {\hat{F}}^{(n, \ell )}, \end{aligned}$$

where $F^{(n, \ell )} \in \Lambda ^{n - \ell } W$ and ${\hat{F}}^{(n, \ell )} \in \bigodot ^{\ell } W$, for any $G \in \Lambda ^m W$ and $G' \in \bigodot ^{m'} W$ we set

5.7 $$\begin{aligned} (G \otimes G') \cdot F :=\sum _{n = 0}^k \sum _{\ell = 0}^n (G \wedge F^{(n, \ell )}) \otimes (G' \odot {\hat{F}}^{(n, \ell )}). \end{aligned}$$

We introduce commutative and anticommutative derivatives on $\mathcal {F}^k (W)$.

#### Definition 5.16

For any $w \in W$, we define two linear maps $\partial ^a_w: \mathcal {F}^k (W) \rightarrow \mathcal {F}^{k - 1} (W)$ and $\partial ^c_w: \mathcal {F}^k (W) \rightarrow \mathcal {F}^{k - 1} (W)$ by the following recursive relation, for any $v \in W$,

$$\begin{aligned} \partial ^a_w (1 \otimes v) = \partial ^c_w (v \otimes 1) = 0, \end{aligned}$$

$$\begin{aligned} \partial ^a_w ((v \otimes 1) \cdot F) = \langle w, v \rangle _W F - (w \otimes 1) \cdot \partial ^a_w F, \qquad \partial ^c_w ((1 \otimes v) \cdot F) = \langle w, v \rangle _h F + (1 \otimes w) \cdot \partial ^a_w F. \end{aligned}$$

If $Z_- : W \rightarrow \mathcal {G}^p_{X, -}$ and $F = \sum _i w_{\alpha _i^1} \wedge \cdots \wedge w_{\alpha _i^k} \in \Lambda ^k W$, $n \leqslant p$, where $w_{\alpha _i^n} \in W$, we can define

$$\begin{aligned} F (Z_-) :=\sum _i Z_- (w_{\alpha ^1_i}) \cdots Z_- (w_{\alpha ^k_i}) \end{aligned}$$

which is a well-defined object (not depending on the explicit representation of *F*) since *Z* is $\mathcal {G}^p_{X, -}$-valued. In the same way if $Z_+ : W \rightarrow \mathcal {G}^p_{X, +}$ and $F = \sum _i w_{\alpha _i^1} \odot \cdots \odot w_{\alpha _i^k} \in \bigodot ^k W$ we can define $F (Z_+) = \sum _i Z_+ (w_{\alpha _i^1}) \cdots Z_+ (w_{\alpha _i^k})$. If $Z: W \rightarrow \mathcal {G}^p_X$ can be decomposed in a unique way $Z = Z_- + Z_+$, where $Z_{\rho } : W \rightarrow \mathcal {G}^p_{X, \rho }$, $\rho \mathop {=}\limits \pm$, for any$F \in \mathcal {F}^k (W)$ we define

5.8 $$\begin{aligned} F (Z) :=\sum _{n = 0}^k \sum _{\ell = 0}^n F^{(n, \ell )} (Z_-) \cdot {\hat{F}}^{(n, \ell )} (Z_+). \end{aligned}$$

With the previous notation we have that if $F, G \in \mathcal {F}^{\infty } (W)$ and *Z* has enough integrability we have $(F \cdot G) (Z) = F (Z) \cdot G (Z)$ (where the first product is the product of polynomials in the sense of definition (5.7), and the second is the product in the twisted space $\mathbb {L}^p$).

This setting allows us to provide a suitable Taylor formula in the Grassmann variables.

#### Lemma 5.17

Consider $F \in \mathcal {F}^k (W)$, let $Z = Z_- + Z_+$ and $R = R_- + R_+$ where $Z_{\rho }, R_{\rho } : W \rightarrow \mathcal {G}^p_{X, \rho }$ for $\rho \mathop {=}\limits \pm$, and let $\{ w_j \}_{j = 1}^N$ be an orthonormal basis of *W*. Then

$$\begin{aligned} F (Z) - F (R)= &   \sum _{n = 0}^k \sum _{\ell = 0}^n \sum _{j_1, \ldots , j_{n - \ell }, j_1', \ldots , j_{\ell }' = 1}^N \frac{1}{(n - \ell ) ! \ell !} \partial ^a_{w_{j_1}} \partial ^a_{w_{j_2}} \cdots \partial ^a_{w_{j_{n - \ell }}} \partial ^c_{w_{j_1'}} \cdots \partial ^c_{w_{j_{\ell }'}} (F) (R) \cdot \\  &   \qquad \cdot \left( \prod _{o = 1}^{n - \ell } (Z_- (w_{j_o}) - R_- (w_{j_o})) \right) \cdot \left( \prod _{o = 1}^{\ell } (Z_+ (w_{j_o'}) - R_+ (w_{j_o'})) \right) . \end{aligned}$$

We omit the proof, it being a simple generalisation of the usual Taylor formula. We are in a position to prove an Itô formula for polynomials of Itô–Grassmann processes.

#### Theorem 5.18

Let $\mathfrak {h}$ be a Hilbert space with conjugation $\Theta$. Let $X = (X_t)_t$ be an analytic GBM indexed by $\mathfrak {h}$ with covariance $G_t = tG$. Let $Y = Y_- + Y_+$ be an Itô–Grassmann process of integrability $p \geqslant 2$, indexed by a finite-dimensional vector space *W*, where $Y_-$ and $Y_+$ are an odd and even Itô–Grassmann random fields such that, for any $w \in W$

$$\begin{aligned} Y_{\rho , t} (w) = Y_{\rho , 0} (w) + \int _0^t K_{\rho , s} (w) \textrm{d}s + \int _0^t \langle H_{\rho , s} (w, \cdot ), \textrm{d}X_s \rangle , \qquad \rho \mathop {=}\limits \pm \end{aligned}$$

where $K_{\rho }: W \rightarrow \mathcal {G}^p_{X, \rho }$, $H_{\rho } (w, \cdot ) \in \mathbb {G}^p_{X, \rho , | G | ; \operatorname {loc}}$, $\rho \mathop {=}\limits \pm$. Consider $F \in \mathcal {F}^n (W)$, where $n \leqslant \frac{p}{2}$, then, for any orthonormal basis $\{ v_j \}_{\alpha = 1}^N$ of *W*, we have

5.9 $$\begin{aligned} F (Y_t) - F (Y_0)= &   \sum _{\alpha } \int _0^t \partial _{v_{\alpha }}^a F (Y_s) \textrm{d}Y^{\alpha }_{-, s} + \int _0^t \partial _{v_{\alpha }}^c F (Y_s) \textrm{d}Y^{\alpha }_{+, s}\nonumber \\  &   + \frac{1}{2} \sum _{\alpha , \beta } \int _0^t \partial _{v_{\alpha }}^a \partial ^a_{v_{\beta }} F (Y_s) \textrm{d}[Y^{\alpha }_-, Y^{\beta }_-]_s \nonumber \\  &   + \frac{1}{2} \sum _{\alpha , \beta } \int _0^t \partial _{v_{\alpha }}^c \partial ^c_{v_{\beta }} F (Y_s) \textrm{d}[Y^{\alpha }_+, Y_+^{\beta }]_s \nonumber \\  &   + \sum _{\alpha , \beta } \int _0^t \partial _{v_{\alpha }}^c \partial ^a_{v_{\beta }} F (Y_s) \textrm{d}[Y^{\alpha }_+, Y^{\beta }_-]_s \end{aligned}$$

where $Y^{\alpha }_{\rho } = Y_{\rho } (v_{\alpha }),$ $\rho = \pm$, and the integrals are defined in terms of *X*, $K_{\rho }$, $H_{\rho }$ for $\rho = \pm$ as in Notation 5.15.

#### Remark 5.19

If *Y* is odd and $F \in \bigoplus _{n = 0}^k \Lambda ^n W$ the composition $F (Y_t)$ is well-defined (as a particular case of equation (5.8)) and Theorem 5.18 implies

5.10 $$\begin{aligned} F (Y_t) - F (Y_0) = \sum _{\alpha } \int _0^t \partial _{v_{\alpha }}^a F (Y_s) \textrm{d}Y^{\alpha }_s + \frac{1}{2} \sum _{\alpha , \beta } \int _0^t \partial _{v_{\alpha }}^a \partial ^a_{v_{\beta }} F (Y_s) \textrm{d}[Y^{\alpha }, Y^{\beta }]_s,\nonumber \\ \end{aligned}$$

which is the perfect analogous of the Itô formula in standard stochastic calculus. If *F* is even and $F \in \bigoplus _{n = 0}^k \bigodot ^n W$ Itô formula (5.10) holds with $\partial _{v_{\alpha }}^c$ in place of $\partial _{v_{\beta }}^a$.

#### Proof of Theorem 5.18

The proof is along the lines of the standard proof of the Itô formula in the commutative setting (see, e.g., [35, Section 5.2]). For this reason, we sketch it in the special case $K_- = K_+ = H_+ = 0$. Furthermore, we consider the convergence in $\mathbb {L}^2$ since $\mathbb {L}^p \subset \mathbb {L}^2$ for any $p > 2$; in fact, by proving the equality in $\mathbb {L}^2$, because both members belong to $\mathbb {L}^p$ we have that the equality holds in $\mathbb {L}^p$ as well.

We write $Y^{\alpha }_t = Y_{-, t} (v_{\alpha })$, and $H_s^{\alpha } (\cdot ) = H_{-, s} (v_{\alpha }, \cdot )$. Furthermore, since in the proof only anticommutative derivatives $\partial ^a$ are needed, we use the notation by $\partial _{v_{\alpha }} \equiv \partial _{v_{\alpha }}^a$, $\partial _{v_{\alpha _1} \ldots v_{\alpha _i}}^i \equiv \partial _{v_{\alpha _1}}^a \cdots \partial _{v_{\alpha _i}}^a$. Being *F* a polynomial of degree at most *n*, for any $\alpha _1, \ldots {,}\alpha _i$ we have

5.11 $$\begin{aligned} \left\| \partial _{v_{\alpha _1} \ldots v_{\alpha _i}}^i F (Y_t) \right\| _{\mathbb {L}^{2 n / (n - i)}} \lesssim (1 + \Vert Y_t \Vert _{\mathbb {L}^{2 n}})^{n - i}, \end{aligned}$$

where the constant in the symbol $\lesssim$ depends only on *F*. Furthermore, by Lemma 5.17, we get

5.12 $$\begin{aligned} \begin{array}{l} F (Y_t) - F (Y_0) = \sum _{t_i \in \pi } F (Y_{t_i}) - F (Y_{t_{i - 1}})\\ \qquad = \sum _{t_i \in \pi } \sum _{\alpha } \partial _{v_{\alpha }} F (Y_{t_{i - 1}}) (Y^{\alpha }_{t_i} - Y^{\alpha }_{t_{i - 1}}) \\ \qquad \quad + \frac{1}{2} \sum _{t_i \in \pi } \sum _{\alpha , \beta } \partial _{v_{\alpha } v_{\beta }}^2 F (Y_{t_{i - 1}}) (Y^{\alpha }_{t_i} - Y^{\alpha }_{t_{i - 1}}) (Y^{\beta }_{t_i} - Y^{\beta }_{t_{i - 1}})\\ \qquad \quad + \sum _{t_i \in \pi } \sum _{i = 3}^n \frac{1}{i!} \sum _{\alpha _1, \ldots , \alpha _i = 1}^k \partial _{v_{\alpha _1} \ldots v_{a_i}}^i F (Y_{t_{i - 1}}) (Y^{\alpha _1}_{t_i} - Y^{\alpha _1}_{t_{i - 1}}) \cdots (Y^{\alpha _i}_{t_i} - Y^{\alpha _i}_{t_{i - 1}}). \end{array} \nonumber \\ \end{aligned}$$

Furthermore, combining (5.11) and (5.12), we get

5.13 $$\begin{aligned} \left\| \partial _{v_{\alpha _1} \ldots v_{\alpha _i}}^i F (Y_t) {-} \partial _{v_{\alpha _1} \ldots v_{\alpha _i}}^i F (Y_s) \right\| _{\mathbb {L}^{2 n / (n - i)}} {\lesssim } (1 + \Vert Y_t \Vert _{\mathbb {L}^{2 n}} {+} \Vert Y_s \Vert _{\mathbb {L}^{2 n}})^{n - i} \Vert Y_t {-} Y_s \Vert _{\mathbb {L}^{2 n}}.\nonumber \\ \end{aligned}$$

Let us focus on each term in the sum in (5.12). For any $| \tau | \leqslant \frac{3}{4}$, we have that

5.14 $$\begin{aligned}  &   \left\| T^{(2)}_{\tau } \left( \sum _{t_i \in \pi } \partial _{v_{\alpha }} F (Y_{t_{i - 1}}) (Y^{\alpha }_{t_i} - Y^{\alpha }_{t_{i - 1}}) - \int _0^t \langle \partial _{v_{\alpha }} F (Y_s) H_{s, -}, \textrm{d}X_s \rangle \right) \right\| _{L^2}^2 \nonumber \\  &   \quad = \left\| T^{(2)}_{\tau } \left( \sum _{t_i \in \pi } \left( \int _{t_{i - 1}}^{t_i} \langle (\partial _{v_{\alpha }} F (Y_{t_{i - 1}}) - \partial _{v_{\alpha }} F (Y_s)) H_{s, -}, \textrm{d}X_s \rangle \right) \right) \right\| ^2_{L^2} \nonumber \\  &   \quad \lesssim \sum _{t_i \in \pi } \int _{t_{i - 1}}^{t_i} \left\| T^{\left( \frac{2 n}{n - 1} \right) }_{\tau _1} (\partial _{v_{\alpha }} F (Y_{t_{i - 1}}) - \partial _{v_{\alpha }} F (Y_s)) \cdot \right. \nonumber \\  &   \qquad \left. \cdot \operatorname {Tr}_{G^{*} \Theta } (T^{(2 n)}_{\tau _2} (H_s) \otimes T^{(2 n)}_{\tau _2} (H^{*}_s)) T^{\left( \frac{2 n}{n - 1} \right) }_{\tau _1} (\partial _{v_{\alpha }} F (Y_{t_{i - 1}}) - \partial _{v_{\alpha }} F (Y_s)) \right\| _{\mathbb {L}^1} \textrm{d}s \nonumber \\  &   \qquad \lesssim \sum _{t_i \in \pi } \sup _{s \in [t_i, t_{i - 1}]} \Vert \partial _{v_{\alpha }} F (Y_{t_{i - 1}}) - \partial _{v_{\alpha }} F (Y_s) \Vert _{\mathbb {L}^{2 n / (n - 1)}}^2\nonumber \\  &   \qquad \qquad \left( \sum _{\alpha } \int _{t_{i - 1}}^{t_i} \Vert \operatorname {Tr}_{G^{*} \Theta } (H_s^{\alpha } \otimes H^{\alpha *}_s) \Vert _{\mathbb {L}^{2 n}} \textrm{d}s \right) \nonumber \\  &   \qquad \lesssim \left( \sum _{\alpha } \int _0^t \Vert H_s^{\alpha } \Vert _{\mathbb {L}^p, | G |} \textrm{d}s \right) (\sup _{s \in [0, t]} (1 + \Vert Y_t \Vert _{\mathbb {L}^p}))^{2 n - 4} \nonumber \\  &   \qquad \qquad (\max _{t_i \in \pi } \sup _{s \in [t_i, t_{i - 1}]} \Vert Y_{t_{i - 1}} - Y_s \Vert _{\mathbb {L}^p}^2), \end{aligned}$$

where $\tau _1, \tau _2 \geqslant 0$ are suitable real numbers, and in the last step we used the inequalities (5.5) and (5.13).

By the inequality (5.13) and by Remark 5.8 applied to the Itô–Grassmann process $Y_t$, the last term goes to zero. Taking the sup over $| \tau | \leqslant \frac{3}{4}$ in (5.14), we obtain that $\sum _{t_i \in \pi } \partial _{v_{\alpha }} F (Y_{t_{i - 1}}) (Y^{\alpha }_{t_i} - Y^{\alpha }_{t_{i - 1}})$ converges to $\int _0^t \langle \partial _{v_{\alpha }} F (Y_s) H_s^{\alpha } (\cdot ), \textrm{d}X_s \rangle$ as $| \pi | \rightarrow 0$ in $\mathbb {L}^2$.

By using the fact that $Y_s^{\alpha } Y_s^{\beta } - [Y^{\alpha }, Y^{\beta }]_s$ is a martingale, see Definition 5.9, we get that, for any $| \tau | \leqslant \frac{3}{4}$,

5.15 $$\begin{aligned}  &   \left\| T^{(2)}_{\tau } \left( \sum _{t_i \in \pi } \partial _{v_{\alpha } v_{\beta }}^2 F (Y_{t_{i - 1}}) \left( (Y^{\alpha }_{t_i} - Y^{\alpha }_{t_{i - 1}}) \left( Y^{\beta }_{t_i} - Y^{\beta }_{t_{i - 1}} \right) \right. \right. \right. \nonumber \\  &   \qquad \qquad \left. \left. \left. - [Y^{\alpha }, Y^{\beta }]_{t_i} + [Y^{\alpha }, Y^{\beta }]_{t_{i - 1}} \right) \right) \right\| _{L^2}^2 = \nonumber \\  &   \quad = \sum _{t_i \in \pi } \left. \left\| T^{(n)}_{\tau _1} \left( (Y^{\alpha }_{t_i} - Y^{\alpha }_{t_{i - 1}}) (Y^{\beta }_{t_i} - Y^{\beta }_{t_{i - 1}}) - \left[ Y^{\alpha }, Y^{\beta } \right] _{t_i} + [Y^{\alpha }, Y^{\beta }]_{t_{i - 1}} \right) T^{\left( \frac{2 n}{n - 2} \right) }_{\tau _2} \right. \right. \nonumber \\  &   \phantom {\sum _{t_i \in \pi }} \left. (\partial _{v_{\alpha } v_{\beta }}^2 F (Y_{t_{i - 1}})) \cdot \cdot T^{\left( \frac{2 n}{n - 1} \right) }_{\tau _2} (\partial _{v_{\alpha } v_{\beta }}^2 F (Y_{t_{i - 1}}))^{*} T^{(n)}_{\tau _2} \left( (Y^{\alpha }_{t_i} - Y^{\alpha }_{t_{i - 1}}) (Y^{\beta }_{t_i} - Y^{\beta }_{t_{i - 1}}) \right. \right. \nonumber \\  &   \qquad \qquad \left. \left. - [Y^{\alpha }, Y^{\beta }]_{t_i} + [Y^{\alpha }, Y^{\beta }]_{t_{i - 1}} \right) ^{*} \right\| _{L^1} \nonumber \\  &   \quad \lesssim \sum _{t_i \in \pi } \Vert (Y^{\alpha }_{t_i} - Y^{\alpha }_{t_{i - 1}}) (Y^{\beta }_{t_i} - Y^{\beta }_{t_{i - 1}}) - [Y^{\alpha }, Y^{\beta }]_{t_i} \nonumber \\  &   \qquad \qquad + [Y^{\alpha }, Y^{\beta }]_{t_{i - 1}} \Vert _{\mathbb {L}^n}^2 \Vert \partial _{v_{\alpha } v_{\beta }}^2 F (Y_{t_{i - 1}}) \Vert _{\mathbb {L}^{2 n / (n - 2)}}^2 \nonumber \\  &   \quad \lesssim (\sup _{s \in [0, t]} (1 + \Vert Y_t \Vert _{\mathbb {L}^{2 n}}))^{2 n - 4} \left( \sum _{t_i \in \pi } \Vert (Y^{\alpha }_{t_i} - Y^{\alpha }_{t_{i - 1}}) (Y^{\beta }_{t_i} - Y^{\beta }_{t_{i - 1}}) \Vert _{\mathbb {L}^n}^2 + \Vert [Y^{\alpha }, Y^{\beta }]_{t_i} \right. \nonumber \\    &   \left. - [Y^{\alpha }, Y^{\beta }]_{t_{i - 1}} \Vert ^2_{\mathbb {L}^n} \right) \nonumber \\  &   \quad \lesssim (\sup _{s \in [0, t]} (1 + \Vert Y_t \Vert _{\mathbb {L}^p}))^{2 n - 4} \left( \sum _{t_i \in \pi } (\Vert H^{\alpha } \Vert ^2_{\tilde{\mathbb {H}}^n_{| G |} ([t_{i - 1}, t_i])} + \Vert H^{\beta } \Vert ^2_{\tilde{\mathbb {H}}^n_{| G |} ([t_{i - 1}, t_i])})^2 \right) \nonumber \\ \end{aligned}$$

where we used that $n \leqslant p / 2$, which goes to zero as $| \pi | \rightarrow 0$ since the map $t \mapsto \Vert H^{\alpha } \Vert ^2_{\tilde{\mathbb {H}}^p_{| G |} ([0, t])}$ (and thus the map $t \mapsto \Vert H^{\alpha } \Vert ^2_{\tilde{\mathbb {H}}^n_{| G |} ([0, t])}$) is an absolutely continuous function. On the other hand, we have that

5.16 $$\begin{aligned}  &   \left\| T^{(2)}_{\tau } \left( \sum _{t_i \in \pi } \partial _{v_{\alpha } v_{\beta }}^2 F (Y_{t_{i - 1}}) \left( [Y^{\alpha }, Y^{\beta }]_{t_i} - [Y^{\alpha }, Y^{\beta }]_{t_{i - 1}} \right) - \int _0^t \partial _{v_{\alpha } v_{\beta }}^2 F (Y_s) \operatorname {Tr}_{G^{*} \Theta } (H_s^{\alpha } (\cdot ) \otimes H_s^{\beta } (\cdot )) \textrm{d}s \right) \right\| _{L^2} \nonumber \\  &   \quad = \left\| T^{(2)}_{\tau } \left( \sum _{t_i \in \pi } \int _{t_{i - 1}}^{t_i} (\partial _{v_{\alpha } v_{\beta }}^2 F (Y_{t_{i - 1}}) - \partial _{v_{\alpha } v_{\beta }}^2 F (Y_s)) \operatorname {Tr}_{G^{*} \Theta } (H_s^{\alpha } (\cdot ) \otimes H_s^{\beta } (\cdot )) \textrm{d}s \right) \right\| _{L^2} \nonumber \\  &   \quad \lesssim \sum _{t_i \in \pi } \int _{t_{i - 1}}^{t_i} \Vert (\partial _{v_{\alpha } v_{\beta }}^2 F (Y_{t_{i - 1}}) - \partial _{v_{\alpha } v_{\beta }}^2 F (Y_s)) \Vert _{\mathbb {L}^{2 n / (n - 2)}} \Vert \operatorname {Tr}_{G^{*} \Theta } (H_s^{\alpha } (\cdot ) \otimes H^{\beta }_s (\cdot )) \Vert _{\mathbb {L}^n} \textrm{d}s \nonumber \\  &   \quad \lesssim (\sup _{s \in [0, t]} (1 + \Vert Y_t \Vert _{\mathbb {L}^{2 p}}))^{n - 3} (\sup _{t_i \in \pi } \sup _{t \in [t_i, t_{i - 1}]} \Vert Y_{t_{i - 1}} - Y_s \Vert _{\mathbb {L}^{2 p}}) (\Vert H^{\alpha } (\cdot ) \Vert ^2_{\tilde{\mathbb {H}}_{| G |}^p ([0, t])} + \Vert H^{\beta } (\cdot ) \Vert ^2_{\tilde{\mathbb {H}}^p_{| G |} ([0, t])}) \nonumber \\ \end{aligned}$$

which converges to 0 by Remark 5.8 applied to the Itô process $Y_t$. Thus, taking the supremum over $| \tau | \leqslant \frac{3}{4}$, the inequalities (5.15) and (5.16) imply that $\frac{1}{2} \sum _{t_i \in \pi } \partial _{v_{\alpha } v_{\beta }}^2 F (Y_{t_{i - 1}}) (Y^{\alpha }_{t_i} - Y^{\alpha }_{t_{i - 1}}) (Y^{\beta }_{t_i} - Y^{\beta }_{t_{i - 1}})$ converges to $\int _0^t \partial _{v_{\alpha }, v_{\beta }}^2 F (Y_s) \operatorname {Tr}_{G^{*} \Theta } (H_s^{\alpha } (\cdot ) \otimes H^{\beta }_s (\cdot )) \textrm{d}s$ in $\mathbb {L}^2$. Finally we have

$$\begin{aligned}&\left\| \sum _{t_i \in \pi } \partial _{v_{\alpha _1} \ldots v_{a_k}}^k F (Y_{t_{i - 1}}) (Y^{\alpha _1}_{t_i} - Y^{\alpha _1}_{t_{i - 1}}) \cdots (Y^{\alpha _i}_{t_i} - Y^{\alpha _i}_{t_{i - 1}}) \right\| _{\mathbb {L}^2}\\&\quad \leqslant \sum _{t_i \in \pi } \left\| \partial _{v_{\alpha _1} \ldots v_{a_k}}^k F (Y_{t_{i - 1}}) (Y^{\alpha _1}_{t_i} - Y^{\alpha _1}_{t_{i - 1}}) \cdots (Y^{\alpha _i}_{t_i} - Y^{\alpha _i}_{t_{i - 1}}) \right\| _{\mathbb {L}^2}\\&\quad \lesssim \sup _{s \in [0, t]} (1 + \Vert Y_s \Vert _{\mathbb {L}^p})^{n - k} \sum _{t_i \in \pi } \Vert Y_{t_i} - Y_{t_{i - 1}} \Vert _{\mathbb {L}^p}^k\\&\quad \lesssim \sup _{s \in [0, t]} (1 + \Vert Y_s \Vert _{\mathbb {L}^p})^{n - k} \sum _{\alpha } \left( \sum _{t_i \in \pi } \left( \int _{t_{i - 1}}^{t_i} \operatorname {Tr}_{| G |} (H_s^{\alpha } (\cdot ) \otimes H_s^{\alpha *} (\cdot )) \textrm{d}s \right) ^{\frac{k}{2}} \right) . \end{aligned}$$

The last term converges to 0 since $k > 2$. This means that

$$\begin{aligned} \begin{array}{l} F (Y_t) - F (Y_0) = \lim _{| \pi | \rightarrow 0} \sum _{t_i \in \pi } F (Y_{t_i}) - F (Y_{t_{i - 1}})\\ \quad = \sum _{\alpha } \int _0^t \langle \partial _{v_{\alpha }} F (Y_s) H_s^{\alpha } (\cdot ), \textrm{d}X_s \rangle + \frac{1}{2} \sum _{\alpha , \beta } \int _0^t \partial _{v_{\alpha }, v_{\beta }}^2 F (Y_s) \operatorname {Tr}_{G^{*} \Theta } (H_s^{\alpha } (\cdot ) \otimes H_s^{\beta } (\cdot )) \textrm{d}s. \end{array} \end{aligned}$$

#### Remark 5.20

The fact that the Itô–Grassmann process *Y* is $\mathcal {G}^p_X$-valued is used in an essential way in the proof for obtaining the last three terms in the Itô formula (5.9), namely the terms involving the quadratic variation. Indeed, in order to get the quadratic variation, we need to pull through the term $(Y^{\alpha }_{t_i} - Y^{\alpha }_{t_{i - 1}}) (Y^{\beta }_{t_i} - Y^{\beta }_{t_{i - 1}})$ in the Taylor expansion which is possible thanks to the anticommutativity or commutativity of the $Y^{\alpha }$’s.

#### Remark 5.21

An important consequence of Theorem 5.18 is that sums and products of Itô–Grassmann processes are again Itô–Grassmann processes. In other words, Itô–Grassmann processes are closed with respect to algebraic operations.

### Girsanov’s formula

In this section, we will prove a form of Girsanov’s theorem involving Itô–Grassmann processes in the algebra $\overline{\mathcal {G}}_X :=\bigcap _{p \geqslant 1} \mathcal {G}^p_X$ (see Remark 5.25 below for more details). Since $\overline{\mathcal {G}}_X$ is only a Fréchet algebra, but the notions of GBM and conditional expectation were so far discussed for filtered modular spaces $(\mathcal {M}, \omega , (\mathcal {M}_t)_t)$, we shall now extend them. First of all, we introduce the notion of signed expectation for a generic Fréchet algebra $\mathcal {A}$.

#### Definition 5.22

Let $\mathcal {A}$ be a Fréchet algebra. We call a continuous linear functional $\overline{\mathcal {E}} : \mathcal {A} \rightarrow \mathbb {C}$ a signed expectation on $\mathcal {A}$ . Let $\{ \mathcal {A}_t \}_{t \in \mathbb {R}_+}$ be a filtration of Fréchet subalgebra of $\mathcal {A}$ and let $\overline{\mathcal {E}}_t : \mathcal {A} \rightarrow \mathcal {A}_t$ be a family of linear continuous operators, we say $\overline{\mathcal {E}}_t$ is a conditional expectation associated with $\overline{\mathcal {E}}$ if for any $a \in \mathcal {A}$, $t, s \in \mathbb {R}^+$, $t \geqslant s \geqslant 0$, $b, c \in \mathcal {A}_s$ we have

1. $$\overline{\mathcal {E}} (\overline{\mathcal {E}}_t (a)) = \overline{\mathcal {E}} (a)$$,
2. $$\overline{\mathcal {E}}_s (\overline{\mathcal {E}}_t (a)) = \overline{\mathcal {E}}_s (a)$$,
3. $$\overline{\mathcal {E}}_s (b a c) = b \overline{\mathcal {E}}_s (a) c$$.

#### Remark 5.23

Note that in a generic Fréchet algebra (without a $^{*}$ operation) we do not have the notion of positive operator and thus of positive linear functional. Also in the particular case of a Fréchet $^{*}$-algebra the expectation $\overline{\mathcal {E}}$ is generally not a positive linear functional.

A process $X \in \mathcal {A}$ is a martingale with respect to the signed expectation $\overline{\mathcal {E}}$ if, as usual,

$$\begin{aligned} \overline{\mathcal {E}_s} (X_t) = X_s, \qquad \forall s \leqslant t. \end{aligned}$$

We can also define a concept of law for random fields with respect to a signed expectation $\overline{\mathcal {E}}$: we say that two random fields $B, B' : \mathfrak {h} \rightarrow \mathcal {A}$, defined on the same vector space $\mathfrak {h}$, have the same law with respect to the signed state $\overline{\mathcal {E}}$ if and only if the *n* points functions of the two fields are the same; i.e. for any $v_1, \ldots , v_n \in \mathfrak {h}$ we have

$$\begin{aligned} \overline{\mathcal {E}} (B (v_1) \cdots B (v_n)) = \overline{\mathcal {E}} (B' (v_1) \cdots B' (v_n)). \end{aligned}$$

We can extend the same notion for processes, and thus we are able to define the notion of a Brownian motion with covariance *G* with respect to $\overline{\mathcal {E}}$ and of martingale.

#### Definition 5.24

Let $\mathcal {A}$ be a Fréchet algebra with signed expectation $\overline{\mathcal {E}}$ and let $\mathcal {B} \subset \mathcal {A}$ be the algebra generated by some Grassmann random variable. Denote by $\mathcal {B}_+$, $\mathcal {B}_- \subset \mathcal {B}$ be the even and odd subspaces respectively. Let $\mathfrak {h}$ be a separable Hilbert space with conjugation $\Theta$. We say that a process $X : \mathbb {R}_+ \times \mathfrak {h} \rightarrow \mathcal {B}_-$ is a weak Grassmann Brownian motion with respect to $\overline{\mathcal {E}}$ and with covariance *G*, if it is a martingale and if, for any $v_1, \ldots , v_{2 n} \in \mathfrak {h}$ and $t_1, \ldots , t_{2 n} \in \mathbb {R}_+$, we have

$$\begin{aligned} \overline{\mathcal {E}} \left( \prod _{i = 1}^{2 n} X_{t_i} (v_i) \right) = \sum _{\mathcal {M} \in \text {Perfect matches of } \{ 1, \ldots , 2 n \} } (- 1)^{\mathcal {M}} \prod _{(i, j) \in \mathcal {M}} \langle \Theta v_i, Gv_j \rangle (t_i \wedge t_j). \end{aligned}$$

Obviously the *n*-point functions of Brownian motion defined with respect a signed state $\overline{\mathcal {E}}$ are equal to the ones of Brownian motion with respect to a positive state, as defined in Definition 4.11.

#### Remark 5.25

Hereafter we consider the following setting: we fix a filtered modular space $(\mathcal {M}, \omega , (\mathcal {M}_t)_t)$, for which there is an analytic Brownian motion *X*, defined on $\mathfrak {h}$, and the algebras $\mathcal {G}_X$ and $\mathcal {G}^p_X$ generated by *X* and by some $\widetilde{\mathcal {M}}_0 \subset \mathcal {M}_0$ (see Definition 5.1). We then consider the space

5.17 $$\begin{aligned} \overline{\mathcal {G}}_X :=\bigcap _{p \geqslant 1} \mathcal {G}^p_X, \end{aligned}$$

which is, thanks to Hölder’s inequality, a Fréchet algebra with the seminorms $\Vert \cdot \Vert _{\mathbb {L}^p}$. We also introduce the spaces $\overline{\mathcal {G}}_{X, \pm } = \bigcap _{p \geqslant 1} \mathcal {G}^p_{X, \pm }$. We call *standard setting* the case in which $\mathcal {A} = \overline{\mathcal {G}}_X$.

In Sect. 2.4 we extended the conditional expectation $\omega _t$ to the $\mathbb {L}^p$ spaces, see Remark 2.24, so that the the state $\omega$ is also a signed expectation with conditional expectation $\omega _t$ on $\overline{\mathcal {G}}_X$ in the sense of Definition 5.22. We shall now describe a standard way of building from $\omega$ and $\omega _t$ a signed expectation $\overline{\mathcal {E}}$ with a conditional expectation $\overline{\mathcal {E}}_t$ on the Fréchet algebra $\overline{\mathcal {G}}_X$.

#### Lemma 5.26

In the standard setting of Remark 5.25, consider a martingale $(Z_t)_{t \in \mathbb {R}_+} \subset \overline{\mathcal {G}}_{X, +}$ such that $Z_0 = 1$, $Z_{\infty } = \lim _{t \rightarrow + \infty } Z_t$ exists, $\omega _t (Z_{\infty }) = Z_t$, and, for any $t \in \mathbb {R}_+$ there is $Z_t^{- 1} \in \overline{\mathcal {G}}_{X, +}$ such that $Z_t \cdot Z_t^{- 1} = 1$. Then, if for every $a \in \overline{\mathcal {G}}_X$ and $t \in \mathbb {R}_+$, we define

5.18 $$\begin{aligned} \overline{\mathcal {E}}^Z (a) = \omega (a Z_{\infty }), \qquad \overline{\mathcal {E}}_t^Z (a) = \omega _t (aZ_{\infty }) Z_t^{- 1}. \end{aligned}$$

Then $\overline{\mathcal {E}}^Z$ is a signed expectation on $\overline{\mathcal {G}}_X$ with associated conditional expectation $\overline{\mathcal {E}}_t^Z$ with respect to the filtration $(\overline{\mathcal {G}}_{X, t})_{t \in \mathbb {R}_+} :=(\overline{\mathcal {G}}_X \cap \mathbb {L}^1 (\mathcal {M}_t))_{t \in \mathbb {R}_+}$ of $\overline{\mathcal {G}}_X$, in the sense of Definition 5.22.

#### Proof

The continuity of $\overline{\mathcal {E}}^Z$ and $\overline{\mathcal {E}}_t^Z$ is given by Hölder’s inequality for the twisted $\mathbb {L}^p$ spaces. Furthermore properties 1, 2, 3 of Definition 5.22 hold. Indeed, we have that, for any $a \in \overline{\mathcal {G}}_X$ and $s \leqslant t$,

$$\begin{aligned} \begin{array}{lll} \overline{\mathcal {E}}_s^Z (\overline{\mathcal {E}}_t^Z (a)) &  = &  \omega _s (\omega _t (a Z_{\infty }) Z_t^{- 1} Z_{\infty }) Z_s^{- 1}\\ &  = &  \omega _s (\omega _t (a Z_{\infty }) Z_t^{- 1} \omega _t (Z_{\infty })) Z_s^{- 1}\\ &  = &  \omega _s (\omega _t (a Z_{\infty })) Z_s^{- 1}\\ &  = &  \overline{\mathcal {E}}_s^Z (a) \end{array} \end{aligned}$$

and similarly in the case where $\overline{\mathcal {E}}_s^Z$ is replaced by $\overline{\mathcal {E}}^Z$. Furthermore for any $b, c \in \overline{\mathcal {G}}_{X, t}$ we get

$$\begin{aligned} \overline{\mathcal {E}}_t^Z (b a c) = \omega _t (b a c Z_{\infty }) Z_t^{- 1} = \omega _t (b a Z_{\infty } c) Z_t^{- 1} = b \omega _t (a Z_{\infty }) c Z_t^{- 1} = b \overline{\mathcal {E}}^Z_t (a) c \end{aligned}$$

where we used that $Z_{\infty }, Z_t^{- 1}$ commute with any element of $\overline{\mathcal {G}}_X$ being both elements in $\overline{\mathcal {G}}_{X, +}$. $\square$

Below, it will be important to characterise weak Grassmann Brownian motion. To this end, we will use the following theorem, which is a non-commutative version ofLévy’s characterization theorem.

#### Theorem 5.27

(Non-commutative Lévy’s characterization). Suppose we are in the standard setting, see Remark 5.25, and let $\overline{\mathcal {E}}$ be a signed expectation on $\overline{\mathcal {G}}_X$ admitting the conditional expectation $\overline{\mathcal {E}}_t$. Consider the Itô–Grassmann process $B_t = \int _0^t \langle H'_s, \textrm{d}X_s \rangle + \int _0^t K_s \textrm{d}s$, with $\Vert H'_{\cdot } \Vert _{\mathbb {L}^p, | G |} \in L^{\infty }_{\operatorname {loc}} (\mathbb {R}_+)$, $K \in L^{\infty }_{\operatorname {loc}} (\mathbb {R}_+, \mathcal {G}^p_X)$, for every $p \geqslant 2$, which is a martingale with respect to $\overline{\mathcal {E}}_t$. Then, *B* is a weak Brownian motion with covariance *G* and with respect to the signed state $\overline{\mathcal {E}}$ if and only if $B_0 = 0$ and the quadratic variation $[B (v), B (v')]_t$ with respect to $\overline{\mathcal {E}}_t$ is $[B (v), B (v')]_t = \langle \Theta v, Gv' \rangle t$.

The proof of Theorem 5.27 requires the following technical lemma.

#### Lemma 5.28

Let $Y, Y'$ be two Itô–Grassmann processes taking values in $\overline{\mathcal {G}}_X$ such that $Y \in L^2_{\operatorname {loc}} (\mathbb {R}_+, \mathcal {G}^{2 p}_X)$ and $Y_t' = \int _0^t \langle H'_s, \textrm{d}X_s \rangle + \int _0^t K_s' \textrm{d}s$ with $s \mapsto \Vert H'_s \Vert _{\mathbb {L}^{2 q}, | G |} \in L^2_{\operatorname {loc}} (\mathbb {R}_+)$, $K \in L^2 (\mathbb {R}_+, \mathcal {G}^{2 q}_X)$ and $\frac{1}{p} + \frac{1}{q} = 1$. Then, for each $t \in \mathbb {R}_+$, the following identity holds in $\mathbb {L}^2$

$$\begin{aligned} \lim _{| \pi | \rightarrow 0} \sum _{t_i \in \pi } Y_{t_{i - 1} \wedge t} (Y_{t_i \wedge t}' - Y_{t_{i - 1} \wedge t}') = \int _0^t Y_s \textrm{d}Y'_s. \end{aligned}$$

#### Proof

The proof is similar to the one of Theorem 5.18 (see in particular Equation (5.18)). $\square$

An important consequence of Lemma 5.28 is the following.

#### Corollary 5.29

Let $Y_t, Y'_t$be two Itô–Grassmann processes satisfying the hypotheses of Lemma 5.28. Then, if $Y'_t$ is a martingale with respect to the signed expectation $\overline{\mathcal {E}}$, also $\int _0^t Y_s \textrm{d}Y'_s$ is a martingale with respect to $\overline{\mathcal {E}}$. Furthermore, if $U, U'$ is an other pair of Itô–Grassmann processes satisfying the hypotheses of Lemma 5.28, such that $U'_t = \int _0^t \langle T_s', \textrm{d}X_s \rangle + \int _0^t L_s \textrm{d}s$ is a martingale with respect to the signed expectation $\overline{\mathcal {E}}$, then the quadratic variation of $\int _0^t Y_s \textrm{d}Y'_s$ and $\int _0^t U_s \textrm{d}U_s'$ with respect to $\overline{\mathcal {E}}$ is

$$\begin{aligned} \left[ \int _0^{\cdot } Y_s \textrm{d}Y'_s, \int _0^{\cdot } {U_s \textrm{d}U'_s} \right] _t = \int _0^t \operatorname {Tr}_{G^{*} \Theta } ((Y_s \cdot H'_s) \otimes (U_s \cdot T_s')) \textrm{d}s. \end{aligned}$$

#### Proof

The proof of the first part is a consequence of the fact that $\sum _{t_i \in \pi } Y_{t_{i - 1} \wedge t} (Y_{t_i \wedge t}' - Y_{t_{i - 1} \wedge t}')$ is a $\overline{\mathcal {E}}$ martingale whenever $Y'$ is a $\overline{\mathcal {E}}$ martingale, and the fact that the martingale property is preserved by taking limits in $\mathbb {L}^2$, $\overline{\mathcal {E}}_t$ being continuous. The second part of the statement is a consequence of the first part and the Itô formula. $\square$

#### Proof of Theorem 5.27

Let $\theta _1, \ldots , \theta _n \in \overline{\mathcal {G}}_{X, -, 0}$ be independent and compatible random variables which are (non-zero) elements of $\widetilde{\mathcal {M}}_0$ and such that the algebra generated by $(\theta _j)_j$ is a Grassmann algebra (by Lemma 4.9 it is always possible to assume that $\widetilde{\mathcal {M}}_0$ contains a numerable set of independent and compatible Grassmann random variables). Consider $f_1, \ldots , f_n : \mathbb {R}_+ \rightarrow \mathfrak {h}$ smooth compactly supported functions. Let ${I \subset [n]} :=\{ 1, \ldots , n \}$ and define

$$\begin{aligned} \mathcal {S}_I (t) = \overline{\mathcal {E}}_0 \left( \prod _{i \in I} \left( 1 + \theta _i \int _0^t \langle f_i (s), \textrm{d}B_s \rangle \right) \right) . \end{aligned}$$

The functions $\mathcal {S}_I$ take value in the finite-dimensional Grassmann algebra $\operatorname {span} \left\{ \prod _{j \in I} \theta _j, I \subset [n] \right\}$. Furthermore, the coefficients of the function $\mathcal {S}_I$ contain all the possible *n*-point functions of the Grassmann random variables $\int _0^t \langle f_i (s), \textrm{d}B_s \rangle$, indeed

$$\begin{aligned} \mathcal {S}_I (t) = \sum _{{J \subset I}} \mathbb {S}_J (t) \theta _J \end{aligned}$$

where $$\theta _J = \prod _{\ell , \{ i_1< \ldots < i_k \} = J} \theta _{i_{\ell }}$$ and

$$\begin{aligned} \mathbb {S}_J (t) = {\operatorname {sign} (J)} \overline{\mathcal {E}} \left( \prod _{\ell , \{ i_1< \ldots < i_k \} = J} \int _0^t \langle f_{i_{\ell }} (s), \textrm{d}B_s \rangle \right) , \end{aligned}$$

for some function $\operatorname {sign} (J) \in \{ \pm \}$. This means that, if for any $I \subset [n]$, the function $\mathcal {S}_I (t)$ is equal to

$$\begin{aligned} \overline{\mathcal {S}}_I (t) = \mathcal {E}_0 \left( \prod _{i \in I} \left( 1 + \theta _i \int _0^t \langle f_i (s), \textrm{d}X_s \rangle \right) \right) \end{aligned}$$

where *X* is some Brownian motion with covariance *G* with respect to $\omega$, then *B* has the same law of a Brownian motion.

Suppose that *B* is a $\overline{\mathcal {E}}$ martingale and that it has quadratic variation with respect to $\overline{\mathcal {E}}$ equal to $\langle G^{*} \Theta \cdot , \cdot \rangle t$. By Corollary 5.29 also $\int _0^t \langle f_j (s) \textrm{d}B_s \rangle$ are martingales with quadratic variation given by $\int _0^t \langle G^{*} \Theta f_i (s), f_j (s) \rangle \textrm{d}s$. This means that, if we apply Itô’s formula to the product $\prod _{i \in I} \left( 1 + \theta _i \int _0^t \langle f_i (s), \textrm{d}B_s \rangle \right)$, we get that $\mathcal {S}_I$ satisfies the following system of ODEs

$$\begin{aligned} \mathcal {S}_I (t) = 1 + \sum _{\{ i, j \} \subset I} \int _0^t \langle G^{*} \Theta f_i (s), f_j (s) \rangle \theta _j \theta _i \mathcal {S}_{I\backslash \{ i, j \}} (s) \textrm{d}s. \end{aligned}$$

Since the previous system of ODEs for $\{ \mathcal {S}_I \}_{I \subset [n]}$ takes value in the finite-dimensional Grassmann algebra $\operatorname {span} \left\{ \prod _{j \in I} \theta _j, I \subset [n] \right\}$ it has a unique solution. On the other hand, using again Itô’s formula, the previous system of ODEs is satisfied by the *n*-point functions of the Brownian motion *X* with covariance *G*, namely

$$\begin{aligned} \overline{\mathcal {S}}_I (t) = 1 + \sum _{\{ i, j \} \subset I} \int _0^t \langle G^{*} \Theta f_i (s), f_j (s) \rangle \theta _j \theta _i \overline{\mathcal {S}}_{I\backslash \{ i, j \}} (s) \textrm{d}s. \end{aligned}$$

This implies that $\{ \mathcal {S}_I (t) \}_{I \subset [n]}$ (where $T \in \mathbb {R}_+$ is such that $\operatorname {supp} (f_j) \subset [0, T]$) are equal to $\{ \overline{\mathcal {S}}_I (t) \}_{I \subset [n]}$, which is equivalent to saying that $B_t$ has, with respect to ${\bar{\omega }}$, the law of the Brownian motion *X* with covariance *G*. The opposite direction of the implication is trivial.

Theorem 5.27 is crucial for proving the following non-commutative version of Girsanov’s theorem.

#### Theorem 5.30

(Non-commutative Girsanov’s formula). Let $(\mathcal {M}, \omega , (\mathcal {M}_t)_t)$ be a filtered modular space and let *X* be an analytic Brownian motion of covariance *G*. Consider a process $H_{\cdot } : \mathbb {R}_+ \times \mathfrak {h} \rightarrow \overline{\mathcal {G}}_{X, -}$, where $\overline{\mathcal {G}}_X$ is the Fréchet algebra introduced in Remark 5.25, and let $Z_t$ be an even adapted process in $\mathbb {L}^2$ satisfying

5.19 $$\begin{aligned} Z_t = 1 + \int _0^t \langle Z_s \cdot H_s, \textrm{d}X_s \rangle . \end{aligned}$$

Let $\overline{\mathcal {E}}^Z$ be the expectation associated with it. Then, the random field such that

$$\begin{aligned} B_t (v) = X_t (v) - \int _0^t H_s (G^{*} \Theta (v)) \textrm{d}s, \qquad \forall v \in \mathfrak {h} \quad t \in \mathbb {R}_+, \end{aligned}$$

is a Brownian motion of covariance *G* with respect to the signed expectation $\overline{\mathcal {E}}^Z$.

#### Remark 5.31

By requiring that $Z \in \mathbb {L}^2$ satisfies equation (5.19), we automatically get that *Z* is a martingale by definition of the Itô integral, see Sect. 3.

#### Proof of Theorem 5.30

The proof follows the same strategy of the commutative case (see, e.g., [35, Section 5.5]). First of all, by (5.18) for the conditional expectation $\overline{\mathcal {E}}_t^Z$, we have that the process $B_t$ is a martingale with respect to $\overline{\mathcal {E}}_t^Z$ if and only if the process $B_t Z_t$ is a martingale with respect to the conditional expectation $\omega _t$. On the other hand, by the Itô formula, the fact that *Z* commutes with *B* and (5.19), we get

$$\begin{aligned} B_t (v) Z_t= &   \int _0^t Z_s \textrm{d}B_s (v) + \int _0^t B_s (v) \textrm{d}Z_s + [B (v), Z]_t \\= &   \int _0^t Z_s \textrm{d}X_s (v) - \int _0^t Z_s H_s (G^{*} \Theta v) \textrm{d}s + \int _0^t \langle B_s (v) Z_s \cdot H_s, \textrm{d}X_s \rangle \\  &   + \int _0^t Z_s H_s (G^{*} \Theta Gv) \textrm{d}s \\= &   \int _0^t Z_s \textrm{d}X_s + \int _0^t \langle B_s (v) Z_s H_s, \textrm{d}X_s \rangle \end{aligned}$$

which is a martingale with respect to $\omega _t$, since it is an Itô integral with respect to *X*. On the other hand, by Corollary 5.29, the quadratic variation of $B_t$ with respect to $\overline{\mathcal {E}}_t^Z$ is

$$\begin{aligned} [B (v), B (v')]_t = [X (v), X (v')]_t = \langle \Theta v, Gv' \rangle . \end{aligned}$$

Thus since *B* is a martingale with respect to $\overline{\mathcal {E}}_t^Z$ with quadratic variation $\langle \Theta v, Gv' \rangle$, by Theorem 5.27, *B* is a weak Brownian motion with respect to $\overline{\mathcal {E}}_t^Z$ with covariance *G*. $\square$

Unlike the commutative case, we do not know if (5.19) has a solution because the exponential of an Itô process in $\overline{\mathcal {G}}_X$ is generally not well-defined. We will now consider some sufficient conditions on *H* such that the solution *Z* to (5.19) exists.

#### Definition 5.32

Let *X* be an analytic Brownian motion of covariance *G*. An adapted process $H_{\cdot } : \mathbb {R}_+ \times \mathfrak {h} \rightarrow \mathcal {G}^X_-$ satisfies the Novikov condition if for every $p \geqslant 2$ we have $\Vert H_{\cdot } \Vert _{\mathbb {L}^p, | G |} \in L^{\infty }_{\operatorname {loc}} (\mathbb {R}_+)$, and for every $t > 0$, we get

$$\begin{aligned} \sum _{n = 0}^{+ \infty } \frac{1}{n!} \left\| \left( \int _0^t \langle H_s, \textrm{d}X_s \rangle - \frac{1}{2} \int _0^t \operatorname {Tr}_{G^{*} \Theta } (H_s \otimes H_s) \textrm{d}s \right) ^n \right\| _{\mathbb {L}^p} < \infty . \end{aligned}$$

Let $H_{\cdot } : \mathbb {R}_+ \times \mathfrak {h} \rightarrow \mathcal {G}_{X, -}$ be an adapted process satisfying the Novikov condition. We set

$$\begin{aligned} Z^{(n)}_t :=\sum _{j = 0}^n \frac{1}{j!} \left( \int _0^t \langle H_s, \textrm{d}X_s \rangle - \frac{1}{2} \int _0^t \operatorname {Tr}_{G^{*} \Theta } (H_s \otimes H_s) \textrm{d}s \right) ^j. \end{aligned}$$

For every $p \geqslant 1$, $Z^{(n)}_t$ converges to

5.20 $$\begin{aligned} Z_t = \sum _{j = 0}^{\infty } \frac{1}{j!} \left( \int _0^t \langle H_s, \textrm{d}X_s \rangle - \frac{1}{2} \int _0^t \operatorname {Tr}_{G^{*} \Theta } (H_s \otimes H_s) \textrm{d}s \right) ^j \end{aligned}$$

in $C^0 (\mathbb {R}_+ ; \mathbb {L}^p)$, and $Z_t$ is an invertible element of $\mathcal {G}^X_+$ with inverse

$$\begin{aligned} Z^{- 1}_t = \sum _{j = 0}^{\infty } \frac{(- 1)^j}{j!} \left( \int _0^t \langle H_s, \textrm{d}B_s \rangle - \frac{1}{2} \int _0^t \operatorname {Tr}_{G^{*} \Theta } (H_s \otimes H_s) \textrm{d}s \right) ^j . \end{aligned}$$

#### Lemma 5.33

Let *X* be an analytic Brownian motion of covariance *G* and let $H_{\cdot } : \mathbb {R}_+ \times \mathfrak {h} \rightarrow \mathcal {G}^X_-$ be an adapted process satisfying the Novikov condition. Then, $Z_t$ defined in (5.20) satisfies (5.19).

#### Proof

By the Itô formula we get that

$$\begin{aligned} Z^{(n)}_t = 1 + \int _0^t \langle Z^{(n - 1)}_s \cdot H_s, \textrm{d}X_s \rangle + \frac{1}{2} \int _0^t (Z^{(n - 2)}_s - Z^{(n - 1)}_s) \cdot \operatorname {Tr}_{G^{*} \Theta } (H_s \otimes H_s) \textrm{d}s. \end{aligned}$$

Taking the limit $n \rightarrow \infty$, which is well-defined by the Novikov condition, we obtain the claim. $\square$

When *H* takes values in $\mathcal {G}^{\infty }_X$ the Novikov condition is automatically satisfied. More precisely, we have the following proposition.

#### Proposition 5.34

Let *X* be an analytic Brownian motion of covariance *G* and let $H_{\cdot } : \mathbb {R}_+ \times \mathfrak {h} \rightarrow \mathcal {G}^X_-$ be an adapted process such that $\operatorname {Tr}_{G^{*} \Theta } (H_{\cdot } \otimes H_{\cdot }) \in L^{\infty } (\mathbb {R}_+ ; \mathcal {G}^{\infty }_X)$. Then, *H* satisfies the Novikov condition.

#### Proof

We note that for any $n \in \mathbb {N}$

5.21 $$\begin{aligned} \frac{1}{n!} \left( \int _0^t \langle H_s, \textrm{d}X_s \rangle - \frac{1}{2} \int _0^t \operatorname {Tr}_{G^{*} \Theta } (H_s \otimes H_s) \textrm{d}s \right) ^n = Z^{(n)}_t - Z^{(n - 1)}_t. \end{aligned}$$

By Theorem 3.5, equation (5.3) and Hölder’s inequality for the twisted $\mathbb {L}^p$ spaces we get

5.22 $$\begin{aligned}  &   \Vert Z^{(n)}_t - Z^{(n + 1)}_t \Vert _{\mathbb {L}^p}\nonumber \\  &   \quad \leqslant C_p \int _0^t \Vert Z^{(n - 1)}_s - Z^{(n)} \Vert _{\mathbb {L}^p} \left( ^{^{}} \Vert \operatorname {Tr}_{G^{*} \Theta } (H_s \otimes H_s) \Vert _{\mathbb {L}^{\infty }} \right) ^{\frac{1}{2}} \textrm{d}s\nonumber \\  &   \qquad + \left. \frac{C_p}{2} \int _0^t \left( ^{^{}} \Vert (Z^{(n - 2)}_s - Z^{(n - 1)}) \Vert _{\mathbb {L}^p} + \Vert Z^{(n - 1)}_s - Z^{(n)} \Vert _{\mathbb {L}^p} \right) \Vert \operatorname {Tr}_{G^{*} \Theta } (H_s \otimes H_s) \Vert _{\mathbb {L}^{\infty }} \textrm{d}s \right) \nonumber \\ \end{aligned}$$

for a suitable constant $C_p > 0$ (depending on $p \geqslant 2$). By induction, it is simple to see that

$$\begin{aligned} \sup _{t \in [0, T]} \Vert Z^{(n)}_t - Z^{(n + 1)}_t \Vert _{\mathbb {L}^p} \leqslant \frac{(3 C_p (1 + \Vert \operatorname {Tr}_{G^{*} \Theta } (H_s \otimes H_s) \Vert _{\mathbb {L}^{\infty }}) (T + 1))^n}{n!}, \end{aligned}$$

hence the claim. $\square$

### Weak solutions of finite-dimensional Grassmann SDE

In this section we define the notion of weak and strong solution to a Grassmann stochastic differential equation (SDE), when the vector space $\mathfrak {h}$ is finite dimensional. First of all we introduce some notation. We note that the space $\mathcal {G}^{\infty }_X = \overline{\mathcal {G}}_X \cap \mathbb {L}^{\infty }$ is a Banach algebra, thus if an adapted process *B* belongs to $C^0 (\mathbb {R}_+; \mathcal {G}^{\infty }_X)$ we can consider the Banach algebra $\mathcal {G}^{\infty }_B$ as the natural Banach subalgebra generated by *B* in $\mathcal {G}^{\infty }_X$.

We now consider two linear functions

5.23 $$\begin{aligned} \mu : \mathfrak {h} \rightarrow \bigoplus _{n = 0}^{+ \infty } \Lambda ^{2 n + 1} \mathfrak {h}, \quad \sigma : \mathfrak {h} \times \mathfrak {h} \rightarrow \left( \bigoplus _{n = 0}^{+ \infty } \Lambda ^{2 n} \mathfrak {h} \right) . \end{aligned}$$

#### Definition 5.35

In a standard setting, let $\overline{\mathcal {E}}$ be a signed expectation on $\overline{\mathcal {G}}_X$ and consider two linear functions $\mu , \sigma$ as in (5.23), we say that the pair of adapted $\overline{\mathcal {G}}_{X, -}$-valued random processes $(\Psi _{\cdot }, B_{\cdot })$ is a weak solution to the SDE $(\mu , \sigma )$ (wrt the signed expectation $\overline{\mathcal {E}}$) on [0, *T*] and with initial condition $\Psi _0$ if *B* is a weak Brownian motion with covariance *G* wrt $\overline{\mathcal {E}}$ and, for any $v \in \mathfrak {h}$ and $t \leqslant T$, the following equation holds:

$$\begin{aligned} \Psi _t (v) - \Psi _0 (v) = \int _0^t \mu (v) (\Psi _s) \textrm{d}s + \int _0^t \langle \sigma (v, \cdot ) (\Psi _s), \textrm{d}B_s \rangle . \end{aligned}$$

Furthermore, if $B \in C^0 (\mathbb {R}_+; \mathcal {G}^{\infty }_X)$ we say that $\Psi$ is a strong solution on [0, *T*] to the SDE $(\mu , \sigma )$ driven by *B* if $(\Psi , B)$ is a weak solution to the SDE $(\mu , \sigma )$ and $\Psi _t \in \mathcal {G}^{\infty }_B$ for any $0 \leqslant t \leqslant T$.

In the special case where $\sigma (v, v') = \langle v, v' \rangle _{\mathfrak {h}}$ (i.e. *additive noise SDEs*) and $\mathfrak {h}$ is finite-dimensional, we can prove the existence of strong solutions.

#### Theorem 5.36

Suppose that $\mathfrak {h}$ is finite-dimensional and let $\sigma (v, v') = \langle v, v' \rangle _{\mathfrak {h}}$ then for any $\Psi _0 \in \mathcal {G}^{\infty }_{X, -} = \mathcal {G}^{\infty }_X \cap \overline{\mathcal {G}}_{X, -}$, $B \in C^0 (\mathbb {R}_+ ; \mathcal {G}^{\infty }_X)$ Brownian motion with respect to $\overline{\mathcal {E}}$, and $T \geqslant 0$, there is a unique strong solution $\Psi _t \in C^0 (\mathbb {R}_+; \mathcal {G}^{\infty }_B)$ to the SDE $(\mu , \sigma )$, driven by *B*.

#### Proof

This theorem, using a slightly difference language and notations, has been proved in  [1, Theorem 30 and 31], in the case where $\Vert B_t - B_s \Vert _{\mathbb {L}^{\infty }} \lesssim | t - s |^{\frac{1}{2}}$. The case where the map $t \mapsto B_t$ is continuous in $\mathbb {L}^{\infty }$ can be proved in a similar way. $\square$

Here we introduce the notation

$$\begin{aligned} \mu ^A (v) :=\mu (v) - A v, \end{aligned}$$

where $A: \mathfrak {h} \rightarrow \mathfrak {h}$ is a linear map.

#### Theorem 5.37

Suppose that $\mathfrak {h}$ is finite-dimensional, suppose that ${\bar{G}} = G^{*} \Theta$ is an invertible antilinear map, and consider $\sigma (v, v') = \langle v, v' \rangle _{\mathfrak {h}}$. Write

$$\begin{aligned} X^A_t (v) = \tilde{X}_0 (\textrm{e}^{At} v) + \int _0^t \langle \textrm{e}^{A (t - s)} (v), \textrm{d}X_s \rangle . \end{aligned}$$

and consider the processes

$$\begin{aligned} Z_t^{\mu ^A}= &   \exp \left( \int _0^t \varvec{1}_{[0, T]} (s) \langle \mu ^A ({\bar{G}}^{- 1} \cdot , (X_s^A + \textrm{e}^{A s} h_0)), \textrm{d}X_s \rangle + \right. \\  &   \left. - \frac{1}{2} \int _0^t \varvec{1}_{[0, T]} (s) \operatorname {Tr}_{{\bar{G}}} (\mu ^A ({\bar{G}}^{- 1} \cdot , (X_s^A {+} \textrm{e}^{A s} h_0)) {\otimes } \mu ^A ({\bar{G}}^{- 1} \cdot , (X_s^A {+} \textrm{e}^{A s} h_0))) \textrm{d}s \right) \\ B_t (\cdot )= &   \int _0^t \varvec{1}_{[0, T]} (s) \mu ^A (\cdot , (X_s^A + h_0 (\textrm{e}^{A s} \cdot ))) \textrm{d}s + X_t. \end{aligned}$$

Then, the pair of processes $(X^A + h_0 (\textrm{e}^{A \cdot }), B)$ is a weak solution of the SDE $(\mu , \sigma )$ on [0, *T*] with initial condition $\tilde{X}_0 + h_0$ and with respect to the expectation $\overline{\mathcal {E}}^{Z^{\mu ^A}}$. Furthermore $X^A_t + h_0 (\textrm{e}^{A t})$ coincides with the strong solution of Theorem 5.36.

#### Proof

First we note that the process $Z_t^{\mu ^A}$ is well-defined. Indeed, $\mu ^A ({\bar{G}}^{- 1} \cdot , (X_s^A + \textrm{e}^{A s} h_0)) \in L^{\infty }_{\operatorname {loc}} (\mathbb {R}_+ ; \mathcal {G}_X^{\infty })$ and by Proposition 5.34 it satisfies the Novikov condition. Furthermore, the process $X^A_t + h_0 (\textrm{e}^{A t})$ solves the SDE $X_t^A (v) + h_0 (\textrm{e}^{A t} v) - \tilde{X}_0 (v) - h_0 (v) = \int _0^t (X^A_s (A v) + h_0 (\textrm{e}^{A t} A v)) \textrm{d}s + X_t (v)$. Which means that we have the following relation, for any $t \leqslant T$

$$\begin{aligned} X_t^A (v) + h_0 (\textrm{e}^{A t} v) - \tilde{X}_0 (v) - h_0 (v) = \int _0^t \mu (v, X^A_s + h_0 (\textrm{e}^{A s} \cdot )) \textrm{d}s + B_t (v). \end{aligned}$$

Since, by Theorem 5.30, $B_t$ is a Brownian motion with respect to the expectation $\overline{\mathcal {E}}^{Z_t^{\mu ^A}}$, Equation (5.4) implies that $(X^A + h_0 (\textrm{e}^{A \cdot }), B)$ is a weak solution to the SDE $(\mu , \sigma )$. Finally, since by Theorem 5.36 the strong solution exists and is unique, we have $\mathcal {G}^{\infty }_X = \mathcal {G}^{\infty }_B$ so that any weak solution is also a strong solution. $\square$

## Applications

### Twisted $L^p$ bound of the exponential

In this section, we prove the existence of a family of weak Gibbsian quartic perturbation of $\omega$, a special case being the Grassmann $\Psi ^4_2$ on the torus. The brevity of the arguments presented should convince the reader about the usefulness of the twisted $\mathbb {L}^p$ spaces introduced in Sect. 2.3. Our strategy is based on the control of the exponential of a Wick’s polynomial in the spirit of the well-known bound by Nelson  [39, 51].

In order to consider spin-$\frac{1}{2}$ fermionic fields on $\mathbb {T}^2$, we let $h = L^2 (\mathbb {T}^2; \mathbb {C}^2)$ and let $(\mathcal {M}^{(\mu )}, \omega , (\mathcal {M}^{(\mu )}_t)_{t \geqslant 0})$ be the filtered modular space associated with $\mathfrak {h} = h \oplus h$, see Sect. 4.2. For the sake of brevity, we henceforth fix $\mu = \frac{1}{2}$ and remove it from our notation. We let $(\Psi _t)_t$ be a GBM with covariance

6.1 $$\begin{aligned} G_t = (1 - \Delta )^{\theta - 1} \chi ((- \Delta )^{1 / 2} / t) (\mathbb {1 \oplus - 1}), \end{aligned}$$

where $- \Delta$ is the Laplacian on *h*, where $\theta \geqslant 0$ and where $\chi \in C^{\infty }_c (\mathbb {R}_+)$ is such that $\chi ([0, {1 / 4}]) = 1$ and $\operatorname {supp} (\chi ) = [0, {1 / 2}]$. We let $\delta _{x, t} (y) = \sum _{k \in \mathbb {Z}^2} \textrm{e}^{\textrm{i}k (y - x)} \varvec{1}_{| k | \leqslant t}$ together with $\delta _{x, t, +} = (\delta _{x, t}, 0)$ and $\delta _{x, t, -} = (0, \delta _{x, t})$, and introduce

$$\begin{aligned} {\bar{\Psi }}_{t, \sigma } (x) :=\Psi _t (\delta _{x, t, \sigma } \oplus 0), \qquad \Psi _{t, \sigma } (x) :=\Psi _t (0 \oplus \delta _{x, t, \sigma }) \qquad \sigma \in \{ \pm \}. \end{aligned}$$

Note that

6.2 $$\begin{aligned} \omega (\Psi _{t, \sigma } (x) {\bar{\Psi }}_{t, \sigma } (y)) = \sum _{k \in \mathbb {Z}^2} \frac{\textrm{e}^{\textrm{i}k (x - y)} \chi (| k | / t)}{(1 + k^2)^{1 - \theta }}, \end{aligned}$$

and that by Proposition 4.14, if $\theta > 0$

6.3 $$\begin{aligned} \sup _{x, \sigma } \Vert \Psi _{t, \sigma } (x) \Vert ^2 \lesssim \sum _{k \in \mathbb {Z}^2} \frac{\chi (| k | / t)}{(1 + k^2)^{1 - \theta }} \sim t^{2 \theta } \end{aligned}$$

whereas the divergence is as $\sim \log t$ if $\theta = 0$. We prove the existence of weak Gibbsian quartic perturbation of $\omega$, for $\theta$ small enough.

#### Theorem 6.1

For any $\lambda \in \mathbb {C}$ and $t \geqslant 0$ define $Z_t^{(\lambda )} = \exp (\lambda V_t)$ with

$$\begin{aligned} V_t :=\int _{\mathbb {T}^2} \llbracket ({\bar{\Psi }}_t (x), \Psi _t (x))^2 \rrbracket \textrm{d}x, \end{aligned}$$

having set $({\bar{\Psi }}_t (x), \Psi _t (x)) = \sum _{\sigma = \pm } {\bar{\Psi }}_{t, \sigma } (x) \Psi _{t, \sigma } (x)$. Then, if ${8} \theta < 1$ and if $| \lambda |$ is small enough depending on $\mu$, the weak limit

6.4 $$\begin{aligned} \omega ^{(\lambda )} (\cdot ) :=\lim _{t \rightarrow \infty } \frac{\omega \left( \cdot \, Z_t^{ (\lambda )} \right) }{\omega (Z_t^{(\lambda )})}, \end{aligned}$$

is a well-defined continuous normalized linear functional on $\mathbb {L}^p$ for any $1 < p \leqslant \infty$.

#### Remark 6.2

In particular, $\omega ^{(\lambda )}$ is a signed expectation on the Fréchet algebra $\overline{\mathcal {G}}_{\Psi }$, see Definition 5.22 and Remark 5.25. The fermionic $\Psi ^4_2$ theory corresponds to the choice $\theta = 0$. Note that the analysis we present can be easily generalized to the case of a general polynomial interaction and of any dimension, provided that the covariance is suitably adjusted.

The claim in Theorem 6.1 is a straightforward consequence of Theorem 6.3 and Corollary 6.5 below. The crucial point here is to show that even though $\lim _{t \rightarrow \infty } Z_t^{(\lambda )}$ is not a bounded random variable, it belongs to the twisted $\mathbb {L}^p$ spaces for any $1 \leqslant p < \infty$.

#### Theorem 6.3

Let ${8} \theta < 1$. Then, for any $\lambda \in \mathbb {C}$ we have $Z_t^{(\lambda )} \in \bigcap _{p < \infty } \mathbb {L}^p$ uniformly in $t \geqslant 0$ and $Z_t^{(\lambda )} \rightarrow Z^{(\lambda )}$ as $t \rightarrow \infty$ in the $\mathbb {L}^p$ topology, for any $1 \leqslant p < \infty$. Furthermore, for any $x \in \mathbb {L}^p$, $1 < p \leqslant \infty$, $\omega (x \cdot Z_t^{(\lambda )})$ has a limit as $t \rightarrow \infty$.

The following technical lemma will be used in the proof of Theorem 6.3.

#### Lemma 6.4

The following bounds hold true for any $\nu < 1 - {4} \theta$ and ${1} \leqslant s \leqslant t$

$$\begin{aligned} \Vert V_t \Vert _{\mathbb {L}^{\infty }} \lesssim t^{4 \theta }, \hspace{6em} \Vert T^{(2)}_{\tau } (V_t - V_s) \Vert _2^2 {\lesssim _{\nu , \tau } s^{- 2 \nu } - t^{- 2 \nu }}, \end{aligned}$$

where the constant in ${\lesssim _{\nu , \tau }}$ is locally bounded in $\tau$.

#### Proof

To prove the first bound, we write the Wick polynomial explicitly

$$\begin{aligned} \llbracket ({\bar{\Psi }}_t (x), \Psi _t (x))^2 \rrbracket = ({\bar{\Psi }}_t (x), \Psi _t (x))^2 + 2 C_t ({\bar{\Psi }}_t (x), \Psi _t (x)) + 2 C_t^2 \end{aligned}$$

with $C_t :=\omega (\Psi _{t, \sigma } (x) {\bar{\Psi }}_{t, \sigma } (x)) \sim t^{2 \theta }$, see (6.2), and therefore, by (6.3) and by Lemma 4.16 we obtain the bound

$$\begin{aligned} \Vert V_t \Vert _{\mathbb {L}^{\infty }} \lesssim \sup _{x \in \mathbb {T}^2} \Vert \llbracket ({\bar{\Psi }}_t (x), \Psi _t (x))^2 \rrbracket \Vert \lesssim t^{4 \theta }. \end{aligned}$$

To prove the other bound, we follow the strategy of  [49], see also  [51], and switch to the Fourier components:

$$\begin{aligned} V_t = \sum _{k_1, \ldots , k_4 \in \mathbb {Z}^2} \varvec{1}_{\sum _i k_i = 0} \left( \prod _i \chi (| k_i | / t) \right) \llbracket (\widehat{{\bar{\Psi }}}_t (k_1), {\hat{\Psi }}_t (k_2)) (\widehat{{\bar{\Psi }}}_t (k_3), {\hat{\Psi }}_t (k_4)) \rrbracket , \end{aligned}$$

where now, denoting spinorial plane waves by $e_{k, \sigma }^+ :=e_{k, \sigma } \oplus 0$, $e_{k, \sigma }^- :=0 \oplus e_{k, \sigma }$, with $e_{k, +} (y) = (\textrm{e}^{\textrm{i}ky}, 0)$ and $e_{k, -} (y) = (0, \textrm{e}^{\textrm{i}ky})$, we set ${\hat{\Psi }}_{t, \sigma } (k ) :=\Psi _t (e^-_{k, \sigma })$ and $\widehat{{\bar{\Psi }}}_{t, \sigma } (k) = \Psi _t (e^+_{k, \sigma })$. With the notation introduced in Lemma 4.12, we also set $e^{\ell }_{k, \sigma , t} :=\mathcal {C}_t e^{\ell }_{k, \sigma }$ together with $\tilde{e}^{\ell }_{k, \sigma , t} :=\tilde{\mathcal {C}}_t e^{\ell }_{k, \sigma }$ and note that, for some constant *C*

6.5 $$\begin{aligned} \langle e^{\ell }_{k, \sigma , t}, e^{\ell '}_{k', \sigma ', s} \rangle _{\mathcal {H}} = \langle \tilde{e}^{\ell }_{k, \sigma , t}, \tilde{e}^{\ell '}_{k', \sigma ', s} \rangle _{\mathcal {H}} = C \delta _{\ell , \ell '} \delta _{\sigma , \sigma '} \delta _{k, k'} \frac{\chi (| k | / (t \wedge s))}{(1 + k^2)^{1 - \theta }}. \end{aligned}$$

Then, by Lemma 4.16, noting that the r.h.s. of (6.5) depends only on $r = t \wedge s$, we have

6.6 $$\begin{aligned} \begin{array}{l} \langle T^{(2)}_{\tau } (\llbracket \widehat{{\bar{\Psi }}}_{t, \sigma } (k_1) {\hat{\Psi }}_{t, \sigma } (k_2) \widehat{{\bar{\Psi }}}_{t, \rho } (k_3) {\hat{\Psi }}_{t, \rho } (k_4) \rrbracket ), \\ \qquad \qquad \qquad T^{(2)}_{\tau } (\llbracket \widehat{{\bar{\Psi }}}_{s, \sigma '} (k_1') {\hat{\Psi }}_{s, \sigma '} (k_2') \widehat{{\bar{\Psi }}}_{s, \rho '} (k_3') {\hat{\Psi }}_{s, \rho '} (k_4') \rrbracket ) \rangle _{L^2}\\ = C_{\tau } \langle (e_{k_1, \sigma , r}^+ \oplus \tilde{e}_{k_1, \sigma , r}^+) \wedge \cdots \wedge (e_{k_4, \rho , r}^- \oplus \tilde{e}_{k_4, \rho , r}^-), \\ \qquad \qquad \qquad (e_{k_1', \sigma ', r}^+ \oplus \tilde{e}_{k_1', \sigma ', r}^+) \wedge \cdots \wedge (e_{k_4', \rho ', r}^- \oplus \tilde{e}_{k_4', \rho ', r}^-) \rangle _{\Gamma _- (\mathcal {H} \oplus \mathcal {H})} \end{array} \end{aligned}$$

for some constant $C_{\tau }$ locally bounded in $\tau$. Therefore, combining (6.5) and (6.6), we have

$$\begin{aligned} \begin{array}{l} \Vert T^{(2)}_{\tau } (V_t - V_s) \Vert _2^2 = \Vert T^{(2)}_{\tau } (V_t) \Vert _2^2 - \Vert T^{(2)}_{\tau } (V_s) \Vert _2^2\\ \quad = C_{\tau } \sum _{k_1, \ldots , k_4 \in \mathbb {Z}^2} \left( \prod _i \chi (| k_i | / t) - \prod _i \chi (| k_i | / s) \right) \frac{\varvec{1}_{\sum _i k_i = 0}}{\prod _i (1 + k_i^2)^{1 - \theta }}, \end{array} \end{aligned}$$

for some other constant $C_{\tau }$ locally bounded in $\tau$. By Young’s inequality for convolutions we obtain, for any $\varepsilon > 0$

6.7 $$\begin{aligned} \Vert T^{(2)}_{\tau } (V_t - V_s) \Vert _2^2 \lesssim _{\tau } \Vert f_{s, t} \Vert _{L^p (\mathbb {R}^2)} \Vert f_{0, t} \Vert _{L^q (\mathbb {R}^2)}^3 {\lesssim _{\varepsilon } s^{- 2 \nu } - t^{- 2 \nu }} \end{aligned}$$

where $\lesssim _{\tau }$ is up to a constant locally bounded in $\tau$, where

$$\begin{aligned} q = \frac{1 + \varepsilon }{1 - \theta }, \hspace{3em} p^{- 1} = 3 \left( \frac{\varepsilon + \theta }{1 + \varepsilon } \right) , \hspace{3em} \nu = {1 - 4 \theta }, \end{aligned}$$

and where $f_{s, t} (k) = (\chi (| k_i | / t) - \chi (| k_i | / s)) (1 + k^2)^{\theta - 1}$. Note that *q* is chosen as small as possible in such a way that $\Vert f_{0, t} \Vert _{L^q (\mathbb {R}^2)} \lesssim _{\varepsilon } 1$ uniformly in $t \geqslant 0$, so that *p* is al large as possible and one gains the decaying factor $\Vert f_{s, t} \Vert _{L^p (\mathbb {R}^2)} \lesssim s^{- 2 \nu } - t^{- 2 \nu }$. This concludes the proof. $\square$

#### Proof of Theorem 6.3

By Hölder’s inequality for the twisted $\mathbb {L}^p$ spaces, we have

6.8 $$\begin{aligned} \Vert Z_t^{(\lambda )} \Vert _{\mathbb {L}^p} \leqslant \sum _{n \geqslant 0} \frac{| \lambda |^n \Vert V_t \Vert _{\mathbb {L}^{pn}}^n}{n!}. \end{aligned}$$

By Lemma 6.4 and by the hypercontractivity bounds, see Lemma 4.20, noting that $C_{\frac{p}{p - 1}, \varrho = \frac{1}{2} \mathbb {1_{\mathcal {H}}}} \lesssim p^{\frac{1}{2}}$, we have for $p \geqslant 2$

6.9 $$\begin{aligned} \Vert T^{(p)}_{\tau } (V_t - V_s) \Vert _p {\lesssim _{\nu , \tau }} p^2 s^{- \nu }. \end{aligned}$$

Therefore, by Proposition 2.19, by Lemma 6.4 and by (6.9) we obtain for any $1 \leqslant p < \infty$

$$\begin{aligned} \Vert V_t \Vert _{\mathbb {L}^p} \leqslant \Vert V_s \Vert _{\mathbb {L}^{\infty }} + \Vert V_t - V_s \Vert _{\mathbb {L}^{p \vee 2}} {\lesssim _{\nu }} s^{4 \theta } + p^2 s^{- \nu }. \end{aligned}$$

In particular, choosing $s = p^{\frac{2}{4 \theta + \nu }}$ we have $\Vert V_t \Vert _{\mathbb {L}^p} {\lesssim _{\nu }} p^{\frac{8 \theta }{4 \theta + \nu }}$. The fact that ${8} \theta < 1$, allows us to choose $\nu < 1 - 4 \theta$ such that $\frac{8 \theta }{4 \theta + \nu } < 1$, hence by (6.8) we obtain

$$\begin{aligned} \Vert Z_t^{(\lambda )} \Vert _{\mathbb {L}^p} \leqslant \sum _{n \geqslant 0} \frac{| \lambda |^n c^n (pn)^{\frac{8 \theta n}{4 \theta + \nu }}}{n!} < \infty , \end{aligned}$$

for some constant *c* and for any $1 \leqslant p < \infty$, hence $Z_t^{(\lambda )} \in \bigcap _{p < \infty } \mathbb {L}^p$ uniformly in $t \geqslant 0$. To prove that the limit exists, we use that $V_t$ are commuting random variables and by the fundamental theorem of calculus write

$$\begin{aligned} \textrm{e}^{\lambda V_t} - \textrm{e}^{\lambda V_s} = \lambda \int _0^1 \textrm{e}^{\lambda V_t (1 - r)} (V_t - V_s) \textrm{e}^{\lambda V_s r} \textrm{d}r. \end{aligned}$$

Therefore, for any $1 \leqslant p < \infty$ and any $s \leqslant t$

$$\begin{aligned} \Vert Z_t^{(\lambda )} - Z_s^{(\lambda )} \Vert _{\mathbb {L}^p} \leqslant | \lambda | (\sup _{r = s, t} \Vert Z_r^{(\lambda )} \Vert _{\mathbb {L}^{2 p}}) \Vert V_t - V_s \Vert _{\mathbb {L}^{2 p}} \lesssim _{\lambda , p} s^{- \nu }, \end{aligned}$$

proving the continuity in the $\mathbb {L}^p$ topology. The continuity of $\omega (x \cdot Z_t^{(\lambda )})$ for $x \in \mathbb {L}^p$, $1 < p \leqslant \infty$ follows by the fact that $x \cdot Z_t^{(\lambda )} \in \mathbb {L}^1$ for any *t* and that

$$\begin{aligned} | \omega (x \cdot Z_t^{(\lambda )}) - \omega (x \cdot Z_s^{(\lambda )}) | = | \omega (x \cdot (Z_t^{(\lambda )} - Z_s^{(\lambda )})) | \leqslant \Vert x \Vert _{\mathbb {L}^p} \Vert Z_t^{(\lambda )} - Z_t^{(\lambda )} \Vert _{\mathbb {L}^{p'}}, \end{aligned}$$

where $${p'}^{- 1} = 1 - p^{- 1} < \infty $$.

On the other hand, the normalization factor can be controlled for small $| \lambda |$.

#### Corollary 6.5

If $| \lambda |$ is small enough, then $| 1 - \omega (Z_t^{(\lambda )}) | \lesssim | \lambda |$ for any $t \geqslant 0$. Furthermore, the limit $\lim _{t \rightarrow \infty } \omega (Z_t^{(\lambda )})$ exists.

#### Proof

Along the lines of the proof of Theorem 6.3, by using the properties of the twisted $\mathbb {L}^p$ spaces we have

$$\begin{aligned} | 1 - \omega (Z_t^{(\lambda )}) | = | \omega (1 - Z_t^{(\lambda )}) | \leqslant \Vert 1 - Z_t^{(\lambda )} \Vert _{\mathbb {L}^1} \leqslant \sum _{n \geqslant 1} \frac{| \lambda |^n c^n n^{\frac{8 \theta n}{4 \theta + \nu }}}{n!} \lesssim _{\theta } | \lambda |. \end{aligned}$$

Furthermore, for any $s \leqslant t$

$$\begin{aligned} | \omega (Z_t^{(\lambda )}) - \omega (Z_s^{(\lambda )}) | = | \omega (Z_t^{(\lambda )} - Z_s^{(\lambda )}) | \leqslant \Vert Z_t^{(\lambda )} - Z_s^{(\lambda )} \Vert _{\mathbb {L}^1} \lesssim s^{- \nu }, \end{aligned}$$

hence the existence of the limit. $\square$

### Weak solution to $\Psi ^4_2$ stochastic quantization SPDE

In this section, we provide the solution to the stochastic quantization equation for a fermionic $\Psi ^4_2$ on the two-dimensional torus $\mathbb {T}^2$ (namely the fermionic SPDE having the signed expectation built in Sect. 6.1 as invariant solution), in the same way in which Jona-Lasinio–Mitter  [26] proved the existence of a weak solution in the bosonic case. More precisely, our strategy is as follows. First, we give a finite-dimensional approximation of the $\Psi ^4_2$ stochastic quantization equation (6.10). Thanks to this finite-dimensional approximation, see (6.13), we can apply the tools developed in Sects. 5.2 and 5.3, namely Itô’s and Girsanov’s formulas, to provide a weak solution to (6.13), compare with Sect. 5.4. Finally, we prove that the process $Z^N$, i.e. the density of the signed expectation $\overline{\mathcal {E}}^{Z^N}$ that appears in Girsanov’s formula, converges to a well-defined random variable *Z* as the $N \rightarrow \infty$. As a result, we obtain a weak solution to the original equation (6.10) under the signed expectation $\overline{\mathcal {E}}^Z$.

Let us now delve into the details of the construction. We consider $h = L^2 (\mathbb {T}^2 ; \mathbb {C}^2)$, $\mathfrak {h} = h \oplus h$ and an analytic Brownian motion *X* with covariance $U = (\mathbb {1 \oplus - 1})$. Hereafter if $\chi : \mathbb {R}_+ \times \mathfrak {h} \rightarrow \mathcal {G}^p_X$ we write $\chi _t (x) = (\chi ^1_t (x), {\bar{\chi }}^1_t (x), \chi ^2_t (x), {\bar{\chi }}^2_t (x))$ to be the limit, if it exists,

$$\begin{aligned} \chi ^j_t (x) :=\lim _{\tau \rightarrow + \infty } \chi _t (\delta _{x, \tau , j} \oplus 0), \quad {\bar{\chi }}^j_t (x) :=\lim _{\tau \rightarrow + \infty } \chi _t (0 \oplus \delta _{x, \tau , j}), \end{aligned}$$

which has to be understood in $\mathcal {S}' (\mathbb {T}^2; \mathcal {G}^p_X)$ (see Appendix A for the definitions of $\mathcal {S} (\mathbb {T}^2 ; \mathcal {G}^p_X)$ and $\mathcal {S}' (\mathbb {T}^2 ; \mathcal {G}^p_X)$). In this way, if $v (x) = (v^1 (x), {\bar{v}}^1 (x), v^2 (x), {\bar{v}}^2 (x)) \in C^{\infty } (\mathbb {T}^2 ; \mathbb {C}^4) \subset \mathfrak {h}$, we have

$$\begin{aligned} \chi _t (v) = \sum _{j = 1, 2} (\langle \chi ^j_t (\cdot ), v^j (\cdot ) \rangle _{\mathcal {S}', \mathcal {S}} + \langle {\bar{\chi }}^j_t (\cdot ), {\bar{v}}^j (\cdot ) \rangle _{\mathcal {S}', \mathcal {S}}). \end{aligned}$$

Using the described identification, when possible, of $\chi : \mathbb {R}_+ \times \mathfrak {h} \rightarrow \mathcal {G}^p_X$ with the function $\chi : \mathbb {R}_+ \rightarrow \mathcal {S}' (\mathbb {T}^2 ; \mathcal {G}^p_X)^4$ allows us to speak about the Besov regularity of a stochastic process. In particular, we say that a stochastic process $\chi : \mathbb {R}_+ \rightarrow \mathcal {S}' (\mathbb {T}^2 ; \mathcal {G}^{\ell }_X)^k$ has Besov regularity $B^s_{p, q}$ if $\chi _{\cdot } (\cdot ) \in C^0 (\mathbb {R}_+ ; B^s_{p, q} (\mathbb {T}^2, \mathcal {G}^{\ell }_X))$ - see Appendix A for the definitions of space of distributions and Besov spaces taking values in a Banach space.

#### Remark 6.6

In the particular case where $\chi _{\cdot } (\cdot ) \in C^0 (\mathbb {R}_+ ; B^s_{p, p} (\mathbb {T}^2 ; \mathcal {G}^p_X))$, we can consider the following sequence of stronger seminorms, for any $T \geqslant 0$

$$\begin{aligned} \Vert \chi \Vert _{C^0 ([0, T]; B^s_{p, p} (\mathbb {T}^2; \mathcal {G}^p_X))}^p \lesssim \sup _{0 \leqslant t \leqslant T} \sum _{j \geqslant - 1} 2^{p s j} \int _{\mathbb {T}^2} \Vert K_j *\chi (x) \Vert _{\mathbb {L}^p}^p \textrm{d}x, \end{aligned}$$

where $K_j = \mathcal {F}^{- 1}_{\mathbb {T}^2} (\varphi _j)$, $j \geqslant - 1$, and $\{ \varphi _j \}_{j \geqslant - 1}$ is a dyadic partition of unity of $\mathbb {R}^2$, see Appendix A.

Using the notation introduced above, the $\Psi ^4_2$ stochastic quantization SPDE reads, for any ${0 \leqslant \kappa < \frac{1}{2}}$, $\lambda \in \mathbb {R}_+$,

6.10 $$\begin{aligned} \chi _t (x)= &   \chi _0 (x) - \int _0^t (A^{1 - 2 \kappa } \chi _s (x) + \lambda A^{- 2 \kappa } (\chi _s | \chi _s |^2) (x)\nonumber \\  &   - \lambda \infty A^{- 2 \kappa } \chi _s) \textrm{d}s + A^{- \kappa } X_t (x), \quad x \in \mathbb {T}^2, \end{aligned}$$

where $$A = (- \Delta + m^2)$$, where

6.11 $$\begin{aligned} | \chi |^2 = \sum _{j = 1, 2} \chi ^{{j}} {\bar{\chi }}^{{j}}, \end{aligned}$$

$\chi = (\chi ^1, {\bar{\chi }}^1, \chi ^2, {\bar{\chi }}^2) \in C^0 (\mathbb {R}_+ ; \mathcal {S}' (\mathbb {T}^2 ; \mathcal {G}^{\ell }_X)^4)$ (for some $\ell \geqslant 3$) and $X_t$ is some Brownian motion of covariance *U*, and the subtraction of $- \infty A^{- 2 \kappa } \chi _s$ stands for a renormalisation procedure which will be explained below. As usual, the necessity of a renormalisation procedure is due to the low expected regularity of the solution to the SPDE (6.10).

#### Lemma 6.7

For any $0 \leqslant \kappa < \frac{1}{2}$, consider the process

6.12 $$\begin{aligned} X_t^A = \textrm{e}^{- A^{1 - 2 \kappa } t} \tilde{X}_0 + \int _0^t \textrm{e}^{- A^{1 - 2 \kappa } (t - s)} A^{- \kappa } \textrm{d}X_s, \end{aligned}$$

where $X_0$ is an analytic (odd) Gaussian with covariance $A^{- 1} U$ independent of the process $(X_t)_{t \geqslant 0}$, then for any, $\varepsilon > 0,$ and $2 \leqslant p \leqslant \infty$ we have $X^A \in C^0 (\mathbb {R}_+ ; B^{- \varepsilon }_{p, p} (\mathbb {T}^2 ; \mathcal {G}^p_X))$.

#### Proof

The proof is given in the case $p = \infty$ in  [1, Lemma 62]. The generic $p \geqslant 2$ can be proved using a similar method and hypercontractivity of Gaussian random variables of Theorem 4.17 (see also  [15] for an analogous proof in the commutative case). $\square$

#### Notation 6.8

Let $\mathfrak {Q (\chi , \psi )}$ be a (local) antisymmetric polynomial of the (regular) random fields, we denote by $\llbracket \mathfrak {Q} (\chi , \psi ) \rrbracket$ the same polynomial where every product between the components of $\chi$ and $\psi$ is replaced by the Wick product, where $\chi , \psi$ are Gaussian random field, more precisely we suppose $\chi = X^A_t$ and $\psi = A^{1 - 2 \kappa } X^A_t$.

For example if $\mathfrak {Q} (\chi , \psi ) = P_N (\chi ^1) (x) P_N ({\bar{\chi }}^1) (x) P_N (\chi ^2) (x) P_N ({\bar{\chi }}^2) (x)$, where $P_N$ is the ($L^2 (\mathbb {T}^2)$-)orthogonal projection on the Fourier modes less or equal than $N \geqslant 0$, and recalling that $\mathcal {E} (P_N (X^{A, 1}_t) (x) P_N ({\bar{X}}^{A, 1}_t) (x)) = \mathcal {E} (P_N (X^{A, 2}_t) (x) P_N ({\bar{X}}^{A, 2}_t) (x)) = C_N$ where $C_N = \sum _{| k | \leqslant N} \frac{1}{| k |^2 + m^2}$ and $\mathcal {E} (X^{A, i} (x) {\bar{X}}^{A, j} (x)) = 0$, for any $i, j = 1, 2$ and , we have

$$\begin{aligned} \llbracket \mathfrak {Q} (\chi , \psi ) \rrbracket = \chi ^1 (x) {\bar{\chi }}^1 (x) \chi ^2 (x) {\bar{\chi }}^2 (x) - C_N \chi ^1 (x) {\bar{\chi }}^1 (x) - C_N \chi ^2 (x) {\bar{\chi }}^2 (x) {- C_N^2}. \end{aligned}$$

Using the symbols introduced in Notation 6.8, we consider an approximate stochastic quantization equation for $x \in \mathbb {T}^2$

6.13 $$\begin{aligned} \left\{ \begin{array}{lll} \chi _t^{(N)} (x) &  = &  \chi _0^{(N)} (x) - \int _0^t [A^{1 - 2 \kappa } \chi _s^{(N)} (x) + \lambda P_N (A^{- 2 \kappa } \llbracket \chi ^{(N)}_s | \chi _s^{(N)} |^2 \rrbracket ) (x)] \textrm{d}s + A^{- \kappa } X_t (x), \\ \chi ^{(N)}_0 (x) &  = &  \tilde{X}_0 (x) + h_0 (x). \end{array} \right. \nonumber \\ \end{aligned}$$

#### Remark 6.9

We take an initial condition of the form Gaussian free field plus some regular random variable, since we want to cover the case where the initial condition is distributed as the non-commutative measure $\omega ^{(\lambda )}$ defined in Theorem 6.1. The fact that a non-commutative random variable distributed as $\omega ^{(\lambda )}$ can be realized as a regular shift of a Gaussian free field is proved in  [16].

Let us now consider the process

$$\begin{aligned} Z^{N, h_0}_t= &   \exp \left( \phantom {- \frac{\lambda ^2}{2}} \lambda \int _0^t \int _{\mathbb {T}^2} P_N (A^{- \kappa } \llbracket \mathfrak {P}_3 (P_N (X^A_s + \textrm{e}^{- A^{1 - 2 \kappa } s} h_0)) \rrbracket ) (x) \textrm{d}X_s (x) \right. \\  &   \left. - \frac{\lambda ^2}{2} \int _0^t \int _{\mathbb {T}^2} \langle P_N (A^{- \kappa } \llbracket \mathfrak {P}_3 (P_N (X^A_s + \textrm{e}^{- A^{1 - 2 \kappa } s} h_0)) \rrbracket ), \right. \\  &   \left. \phantom {- \frac{\lambda ^2}{2}} U A^{- \theta } \llbracket \mathfrak {P}_3 (P_N (X^A_s + \textrm{e}^{- A^{1 - 2 \kappa } s} h_0)) \rrbracket \rangle _{\mathbb {R}^4} \textrm{d}x \textrm{d}s \right) , \end{aligned}$$

where

$$\begin{aligned} \mathfrak {P}_3 (P_N (X^A_s + \textrm{e}^{- A^{1 - 2 \kappa } s} h_0)) = P_N (X^A_s + \textrm{e}^{- A^{1 - 2 \kappa } s} h_0) | P_N (X^A_s + \textrm{e}^{- A^{1 - 2 \kappa } s} h_0) |^2, \end{aligned}$$

and the Itô random field

$$\begin{aligned} B^{N, h_0}_t (x)= &   X_t (x)\\  &   + \int _0^t P_N (A^{- 2 \kappa } (P_N (X^A_s + \textrm{e}^{- A^{1 - 2 \kappa } s} h_0) | P_N (X^A_s + \textrm{e}^{- A^{1 - 2 \kappa } s} h_0) |^2)) (x) \textrm{d}s. \end{aligned}$$

The reason of the introduction of the previous processes is the following weak representation of the solution to the approximating SPDE (6.13).

#### Proposition 6.10

For any $h_0 \in C^1 (\mathbb {T}^2 ; \mathcal {G}^{\infty }_X)$ and for any $\varepsilon > 0$, there is a unique (global in time) strong solution to $\chi ^{(N)}_t \in C^0 (\mathbb {R}_+ ; C^{- \varepsilon } (\mathbb {T}^2 ; \mathcal {G}^{\infty }_X))$ to equation (6.13) driven by the Brownian motion $X_t$. Furthermore the couple of processes $(X^A + \textrm{e}^{A^{1 - 2 \kappa } \cdot } h_0, B^{N, h_0})$ is a weak solution to the SPDE (6.13) with respect to the expectation $\overline{\mathcal {E}}^{Z^{N, h_0}} (\cdot ) = \mathcal {E}_0 (\cdot Z_T^{N, h_0})$, namely we have that, for any polynomial $F \in \bigoplus _{n = 0}^k \Lambda ^n (\mathcal {S} (\mathbb {T}^2)^r)$ and for any $t_1< \ldots < t_r \in \mathbb {R}_+$,

6.14 $$\begin{aligned} \omega _0 (F (\chi _{t_1}^{(N)}, \ldots , \chi _{t_r}^{(N)}))= &   \overline{\mathcal {E}}_0^{Z^{N, h_0}} (F (X^A_{t_1} + \textrm{e}^{- A^{1 - 2 \kappa } t_1} h_0, \ldots , X^A_{t_r} + \textrm{e}^{- A^{1 - 2 \kappa } t_r} h_0)) \nonumber \\= &   \mathcal {E}_0 (F (X^A_{t_1} + \textrm{e}^{- A^{1 - 2 \kappa } t_1} h_0, \ldots , X^A_{t_r} + \textrm{e}^{- A^{1 - 2 \kappa } t_r} h_0) Z_{t_r}^{N, h_0}). \nonumber \\ \end{aligned}$$

#### Proof

The result follows from Theorems 5.36 and 5.37 by noting that it is possible to split equation (6.13) into two independent equations, by projecting the solution on the image of the projection $P_N$ and $I - P_N$. The first equation is a non-linear finite-dimensional equation, and thus Theorems 5.36 and 5.37 apply directly. The second equation is the linear equation (6.12) projected on the image of $I - P_N$. A linear equation of the form (6.12) has always global in time solution and, by the independence of the processes $P_N (\chi ^{(N)}_t)$ and $Z^{N, h_0}$, equality (6.14) can be checked directly. $\square$

In order to take the limit $N \rightarrow \infty$, in the weak solution $(X^A, B^{N, h_0})$ and obtain a weak solution to the limit equation (6.10), we first need a result on the regularity of Wick’s polynomials of $X^A_t$.

#### Lemma 6.11

Let $\mathfrak {Q} (\chi , \psi )$ be any antisymmetric local polynomial of the random fields $\chi , \psi$ of degree *n*, which is at most of first degree in $\psi$. Then, for any $\frac{2 n (n - 2) + 3}{4 n (n - 2) + 8}< \kappa < \frac{1}{2}$, $p \geqslant 2$ and $0 \leqslant s < \frac{2 n (n - 2) + 4}{n} \kappa - \frac{2 n (n - 2) + 3}{2 n}$, the sequence of random fields $\llbracket \mathfrak {Q} (P_N (X^A_t), P_N (A^{1 - 2 \kappa } X^A_t)) \rrbracket$ is a Cauchy sequence in $C^0 \left( \mathbb {R}_+ ; B^{s - \frac{1}{2}}_{p, p} (\mathbb {T}^2 ; \mathcal {G}^p_X) \right)$. We denote the limit by $\llbracket \mathfrak {Q} (X^A_t, A^{1 - 2 \kappa } X^A_t) \rrbracket$. If $\mathfrak {Q}$ does not depend on $\psi$, then $\llbracket \mathfrak {Q} (P_N (X^A_t)) \rrbracket \rightarrow \llbracket \mathfrak {Q} (X_t^A) \rrbracket$ in $C^0 (\mathbb {R}_+ ; B^{- \varepsilon }_{p, p} (\mathbb {T}^2 ; \mathcal {G}^p_X))$ for any $\varepsilon > 0.$ Finally, we have that, for any $T \geqslant 0$,

6.15 $$\begin{aligned} \sup _{t \in [0, T]} \Vert \mathfrak {Q} (X^A_t, A^{1 - 2 \kappa } X^A_t) - \mathfrak {Q} (P_N (X^A_t), P_N (A^{1 - 2 \kappa } X^A_t)) \Vert _{B^{s - \frac{1}{2}}_{p, p} (\mathbb {T}^2; \mathcal {G}^p_X)} {\lesssim p^{\nu (\kappa , s)}} \nonumber \\ \end{aligned}$$

6.16 $$\begin{aligned} \sup _{t \in [0, T]} \Vert \mathfrak {Q} (X^A_t) - \mathfrak {Q} (P_N (X^A_t)) \Vert _{B^{- \varepsilon }_{p, p} (\mathbb {T}^2; \mathcal {G}^p_X)} {\lesssim p^{{\tilde{\nu }} (\kappa , \varepsilon )}} \end{aligned}$$

for some $\nu (\kappa , s), {\tilde{\nu }} (\kappa , \varepsilon ) < 1$, not depending on *T*.

#### Proof

We give the proof for the case where $\mathfrak {Q} (\chi , \psi )$ is a monomial of the form $\mathfrak {Q} (\chi , \psi ) = \psi ^r \prod _{k_1 = 1}^{n_2} \chi ^{j_{k_1}} \prod _{k_2 = 1}^{n_2} {\bar{\chi }}^{j_{k_2}}$ or $\mathfrak {Q} (\chi , \psi ) = \prod _{k_1 = 1}^{n_2} \chi ^{j_{k_1}} \prod _{k_2 = 1}^{n_2} {\bar{\chi }}^{j_{k_2}}$, for some $n_1, n_2, j_{k_1}, j_{k_2}, r \in \mathbb {N}$. By linearity the general result follows.

Let us call

$$\begin{aligned} \begin{array}{rll} V^{j, r}_N &  :=&  \int _{\mathbb {T}^2} \llbracket P_N (A^{1 - 2 \kappa } X^{A, r}_t) \prod _{k_1 = 1}^{n_2} P_N \left( {\bar{X}}^{A, j_{k_1}}_t \right) \prod _{k_2 = 1}^{n_2} P_N \left( {\bar{X}}^{A, j_{k_2}}_t \right) \rrbracket (x) K_j (x) \textrm{d}x,\\ V^j_N &  :=&  \int _{\mathbb {T}^2} \llbracket \prod _{k_1 = 1}^{n_2} P_N \left( {\bar{X}}^{A, j_{k_1}}_t \right) \prod _{k_2 = 1}^{n_2} P_N \left( {\bar{X}}^{A, j_{k_2}}_t \right) \rrbracket (x) K_j (x) \textrm{d}x, \end{array} \end{aligned}$$

where $K_j = \mathcal {F}^{- 1} (\varphi _j)$ is the function corresponding to the *j*-th Littlewood–Paley block.

By the invariance of the law, and of the norm of $P_N (X^A_t)$ and $P_N (A^{1 - \kappa } {\bar{X}}^A_t)$ with respect to spatial and temporal translation, we have that, for any $N, N' \in \mathbb {N}$,

6.17 $$\begin{aligned}  &   \sup _{t \in [0, T]} \Vert \llbracket \mathfrak {Q} (P_N (X^A_t), P_N (A^{1 - 2 \kappa } X^A_t)) \rrbracket - \llbracket \mathfrak {Q} (P_{N'} (X^A_t), P_{N'} (A^{1 - 2 \kappa } X^A_t)) \rrbracket \Vert _{B^{s - \frac{1}{2}}_{p, p}}^p \nonumber \\  &   \quad \lesssim \sum _{r = 1}^4 \sum _{j \geqslant - 1} 2^{\left( s - \frac{1}{2} \right) j p} \Vert V^{j, r}_N - V_{N'}^{j, r} \Vert ^p_{\mathbb {L}^p} \sup _{t \in [0, T]} \Vert \llbracket \mathfrak {Q} (P_N (X^A_t), P_N (A^{1 - 2 \kappa } X^A_t)) \rrbracket \Vert _{B^{s - \frac{1}{2}}_{p, p}}^p \end{aligned}$$

6.18 $$\begin{aligned}  &   \quad \lesssim \sum _{r = 1}^4 \sum _{j \geqslant - 1} 2^{- \left( s - \frac{1}{2} \right) j p} \Vert V^{j, r}_N \Vert ^p_{\mathbb {L}^p} \textrm{d}x, \end{aligned}$$

and similarly for $V^j_N$. Since $\llbracket \mathfrak {Q} (P_N (X^A_t), P_N (A^{1 - 2 \kappa } X^A_t)) \rrbracket$, is a polynomial of $P_N (X^A_t)$ and $P_N (A^{1 - \kappa } {\bar{X}}^A_t)$ with coefficient bounded by $\log (N)$ and $N^{2 - 4 \kappa }$, by following the proof of Lemma 6.4 we get

$$\begin{aligned} \sup _{t \in [0, T], x \in \mathbb {T}^2} \Vert \llbracket \mathfrak {Q} (P_N (X^A_t), P_N (A^{1 - 2 \kappa } X^A_t)) \rrbracket (x) \Vert _{\mathbb {L}^{\infty }}^2 \lesssim (\log N)^{n_1 + n_2} N^{4 - 8 \kappa }, \end{aligned}$$

and thus, since $\Vert K_j \Vert _{L^1 (\mathbb {T}^2)} = 1$, $\Vert V^{j, r}_N \Vert _{\mathbb {L}^{\infty }} \lesssim \Vert K_j \Vert _{L^1 (\mathbb {T}^2)} (\log N)^{n_1 + n_2} N^{4 - 8 \kappa } \lesssim (\log N)^{n_1 + n_2} N^{4 - 8 \kappa }$ (when $V^{j, r}_N$ is replaced by $V^j_N$ we obtain a bound proportional to $(\log (N))^{n_1 + n_2}$).

Following again the proof of Lemma 6.4, we obtain that

6.19 $$\begin{aligned}  &   \Vert T^{(2)}_{\tau } (V^{j, r}_{N'}) - T^{(2)}_{\tau } (V^{j, r}_N) \Vert _{L^2}^2 \nonumber \\  &   \quad = C_{\tau } \sum _{h, \ell _1, \ldots , \ell _{n_1 + n_2} \in \mathbb {Z}^2} \varphi _j^2 \left( h + \sum _{i = 1}^{n_1 + n_2} \ell _i \right) \cdot \nonumber \\  &   \qquad \cdot \left( \varvec{1}_{B_1} \left( \frac{| h |}{N'} \right) \prod _{i = 1}^{n_1 + n_2} \varvec{1}_{B_1} \left( \frac{| \ell _i |}{N'} \right) - \varvec{1}_{B_1} \left( \frac{| g |}{N} \right) \prod _{i = 1}^{n_1 + n_2} \mathbb {I}_{B_1} \left( \frac{| \ell _i |}{N} \right) \right) \cdot \nonumber \\  &   \qquad \cdot \frac{1}{{(| h |^2 + m^2)^{4 \kappa - 1}} } \prod _{i = 1}^{n_1 + n_2} \frac{1}{(| \ell _i |^2 + m^2)} \nonumber \\  &   \quad = C_{\tau } \sum _{h, \ell _1, \ldots , \ell _{n_1 + n_2} \in \mathbb {Z}^2} \varphi _j^2 \left( h + \sum _{i = 1}^{n_1 + n_2} \ell _i \right) \cdot \nonumber \\  &   \qquad \cdot \left( \varvec{1}_{B_{N'} \backslash B_N} (| h |) \prod _{i = 1}^{n_1 + n_2} \varvec{1}_{B_1} \left( \frac{| \ell _i |}{N'} \right) \right) \frac{1}{{(| h |^2 + m^2)^{4 \kappa - 1}} } \prod _{i = 1}^{n_1 + n_2} \frac{1}{(| \ell _i |^2 + m^2)} \nonumber \\  &   \qquad + \sum _{h, \ell _1, \ldots , \ell _{n_1 + n_2} \in \mathbb {Z}^2} \sum _{k = 1}^{n_1 + n_2} \varphi _j^2 \left( h + \sum _{i = 1}^{n_1 + n_2} \ell _i \right) \cdot \nonumber \\  &   \qquad \cdot \left( \varvec{1}_{B_{N'} \backslash B_N} (| \ell _k |) \varvec{1}_{B_1} \left( \frac{| h |}{N} \right) \prod _{i = k + 1}^{n_1 + n_2} \varvec{1}_{B_1} \left( \frac{| \ell _i |}{N} \right) \prod _{i = 1}^{k - 1} \varvec{1}_{B_1} \left( \frac{| \ell _i |}{N'} \right) \right) \cdot \nonumber \\  &   \qquad \cdot \frac{1}{{(| h |^2 + m^2)^{4 \kappa - 1}} } \prod _{i = 1}^{n_1 + n_2} \frac{1}{(| \ell _i |^2 + m^2)}. \end{aligned}$$

Following the proof of  [15, Lemma 3.2], we get

$$\begin{aligned} (6.19) \lesssim 2^{- j \tilde{s}} \left( \Vert {\tilde{\gamma }}_{N, N'} \gamma _{N'}^{n_1 + n_2} \Vert _{H^{1 + \tilde{s}}} + \sum _{k = 1}^{n_1 + n_2} \Vert {\tilde{\gamma }}_N \gamma _{N'}^{k - 1} \gamma ^{n_1 + n_2 - k}_N \gamma _{N, N'} \Vert _{H^{1 + \tilde{s}}} \right) \end{aligned}$$

where

$$\begin{aligned} \begin{array}{rllllll} \gamma _L (x) &  = &  \sum _{| k | \leqslant L, k \in \mathbb {Z}^2} \frac{\textrm{e}^{i k \cdot x}}{(m^2 + | k |^2)}, &  \hspace{3em} &  \gamma _{L,' L} (x) &  = &  \sum _{L< | k | \leqslant L', k \in \mathbb {Z}^2} \frac{\textrm{e}^{i k \cdot x}}{(m^2 + | k |^2)},\\ {\tilde{\gamma }}_L (x) &  = &  \sum _{| k | \leqslant L, k \in \mathbb {Z}^2} \frac{\textrm{e}^{i k \cdot x}}{(m^2 + | k |^2)^{2 \theta - 1}}, &  &  {\tilde{\gamma }}_{L,' L} (x) &  = &  \sum _{L < | k | \leqslant L', k \in \mathbb {Z}^2} \frac{\textrm{e}^{i k \cdot x}}{(m^2 + | k |^2)^{2 \theta - 1}}. \end{array} \end{aligned}$$

If we choose, $\tilde{s} < 0$, $\delta , \delta ' > 0$ and $p', q' \geqslant 1$ such that

$$\begin{aligned} \tilde{s}> 2 s - 1, \quad \delta , \delta '> (n - 2) (2 - 4 \theta ), \quad \delta > (\tilde{s} + 1) - \frac{1}{q' (n - 1)}, \end{aligned}$$

$$\begin{aligned} 8 \kappa - 4 - \delta ' > (\tilde{s} + 1) - \frac{1}{p'} \quad \frac{1}{p'} + \frac{1}{q'} = 1 \end{aligned}$$

(the existence of the previous constant is ensured by the conditions $\frac{2 n (n - 2) + 3}{4 n (n - 2) + 8}< \kappa < \frac{1}{2}$, and $0 \leqslant s < \frac{2 n (n - 2) + 4}{n} \kappa - \frac{2 n (n - 2) + 3}{2 n}$), using the Besov embedding Theorem A.3 for the computation of $\Vert {\tilde{\gamma }}_{N, N'} \Vert _{B^{1 + \tilde{s}}_{p, p}}$, $\Vert \gamma _{N, N'} \Vert _{B^{1 + \tilde{s}}_{p, p}}$, and so on we get that

6.20 $$\begin{aligned} \Vert V^{j, r}_{N'} - V^{j, r}_N \Vert _{\mathbb {L}^2}^2 = \sup _{| \tau | \leqslant \frac{3}{4}} \Vert T^{(2)}_{\tau } (V^{j, r}_{N'}) - T^{(2)}_{\tau } (V^{j, r}_N) \Vert _{L^2}^2 \lesssim 2^{- j \tilde{s}} (N^{- \delta '} + N^{- \delta }).\nonumber \\ \end{aligned}$$

Since $\llbracket \mathfrak {Q} (P_N (X^A_t), P_N (A^{1 - 2 \kappa } X^A_t)) \rrbracket$ is a polynomial of degree $n = n_1 + n_2 + 1$, by the hypercontractivity inequalities (6.17) and (6.20) we have that $\llbracket \mathfrak {Q} (P_N (X^A_t), P_N (A^{1 - 2 \kappa } X^A_t)) \rrbracket$ is a Cauchy sequence in $C^0 \left( \mathbb {R}_+ ; B^{s - \frac{1}{2}}_{p, p} (\mathbb {T}^2 ; \mathcal {G}_X^p) \right)$. In order to get inequality (6.15), we proceed as in the proof of Theorem 6.3. Indeed, for any $p \geqslant 2$, we have, for any $N' > 0$,

6.21 $$\begin{aligned} \Vert V^{j, r}_{N'} \Vert _{\mathbb {L}^p}\leqslant &   \Vert V^{j, r}_N \Vert _{\mathbb {L}^{\infty }} + \Vert V^{j, r}_{N'} - V^{j, r}_N \Vert _{\mathbb {L}^p}\nonumber \\\lesssim &   (\log N)^{n_1 + n_2} N^{2 - 4 \kappa } + p^{\frac{n}{2}} 2^{- j \tilde{s}} (N^{- \delta } + N^{- \delta '}). \nonumber \\\lesssim &   2^{j \tilde{s}} \left( (\log N)^{n_1 + n_2} N^{2 - 4 \kappa } + p^{\frac{n}{2}} (N^{- \delta } + N^{- \delta '}) \right) \end{aligned}$$

By our assumptions on $\delta , \delta '$ there is $\alpha < \frac{1}{2 - 4 \kappa }$ such that, $(2 - 4 \kappa ) \alpha < \nu (\kappa , s)$, $\frac{n}{2} - \alpha \delta ' \leqslant \nu (\kappa , s)$ and $\frac{n}{2} - \alpha \delta \leqslant \nu (\kappa , s)$, for a suitable $0< \nu (\kappa , s) < 1$. This mean that by choosing $N = p^{\alpha }$ we get

$$\begin{aligned} \Vert V^{j, r}_{N'} \Vert _{\mathbb {L}^p} \lesssim 2^{j \tilde{s}} \left( (\log p)^{n_1 + n_2} p^{\alpha (2 - 4 \theta )} + p^{\frac{n}{2} - \alpha \delta } + p^{\frac{n}{2} - \alpha \delta '} \right) \lesssim 2^{j \tilde{s}} p^{\nu (\theta , s)}. \end{aligned}$$

By using inequality (6.18) the claim (6.15) follows.

In order to prove the convergence $\llbracket \mathfrak {Q} (P_N (X^A_t)) \rrbracket \rightarrow \llbracket \mathfrak {Q} (X_t^A) \rrbracket$ and inequality (6.16), we can repeat the previous reasoning by replacing $V^{j, r}_N$ by $V^j$. The reason for the arbitrary (negative) Besov regularity $- \varepsilon$ in this second case is due to the fact that $\Vert V_N^j \Vert _{\mathbb {L}^{\infty }} \lesssim (\log (N))^{n_1 + n_2}$. $\square$

We observe now that, by the Itô formula,

6.22 $$\begin{aligned} Z^{N, h_0}_t= &   \exp \left( \lambda \phantom {- \frac{\lambda ^2}{2}} \int _{\mathbb {T}^2} \llbracket P_N (| P_N (X^A_t + \textrm{e}^{- A^{1 - 2 \kappa } s} h_0) |^4) \rrbracket \textrm{d}x\right. \nonumber \\  &   - \lambda \int _{\mathbb {T}^2} \llbracket P_N (| P_N (\tilde{X}_0 + h_0) |^4) \rrbracket \textrm{d}x \nonumber \\  &   + \lambda \int _0^t \int _{\mathbb {T}^2} \llbracket [P_N (X^A_s + \textrm{e}^{- A^{1 - 2 \kappa } s} h_0)\nonumber \\  &   \cdot A^{1 - 2 \kappa } (P_N (X^A_s + \textrm{e}^{- A^{1 - 2 \kappa } s} h_0))] | P_N (X^A_t + \textrm{e}^{- A^{1 - 2 \kappa } s} h_0) |^2 \rrbracket \textrm{d}x \textrm{d}s \nonumber \\  &   \left. - \frac{\lambda ^2}{2} \int _0^t \int _{\mathbb {T}^2} \langle P_N (A^{- \kappa } \llbracket \mathfrak {P}_3 (P_N (X^A_s + \textrm{e}^{- A^{1 - 2 \kappa } s} h_0)) \rrbracket ), \right. \nonumber \\  &   \left. \phantom {- \frac{\lambda ^2}{2}} \left. \qquad U A^{- \kappa } \llbracket \mathfrak {P}_3 (P_N (X^A_s + \textrm{e}^{- A^{1 - 2 \kappa } s} h_0)) \rrbracket \right\rangle _{\mathbb {R}^4} \textrm{d}x \textrm{d}s \right) , \end{aligned}$$

where we use the notation

$$\begin{aligned} \chi \cdot \psi = \sum _{j = 1, 2} (\chi ^i {\bar{\psi }}^i + {\bar{\chi }}^i \psi ^i). \end{aligned}$$

#### Lemma 6.12

Consider $\frac{19}{40}< \kappa < \frac{1}{2}$. Under the hypotheses of Proposition 6.10, the terms in the sum defining the exponential (6.22) (namely $\llbracket | (X^A_t + \textrm{e}^{- A^{1 - 2 \kappa } t} h_0) |^4 \rrbracket$, $\llbracket P_N (| P_N (\tilde{X}_0 + h_0) |^4) \rrbracket$, etc.) converge, as $N \rightarrow \infty$, to some well defined random processes which we denote by

6.23 $$\begin{aligned}  &   \llbracket | (X^A_t + \textrm{e}^{- A^{1 - 2 \kappa } t} h_0) |^4 \rrbracket , \quad \llbracket [(X^A_s + \textrm{e}^{- A^{1 - 2 \kappa } s} h_0)\nonumber \\  &   \qquad \cdot A^{1 - 2 \kappa } (X^A_s + \textrm{e}^{- A^{1 - 2 \kappa } s} h_0)] | X^A_t + \textrm{e}^{- A^{1 - 2 \kappa } s} h_0 |^2 \rrbracket ,\nonumber \\ \end{aligned}$$

6.24 $$\begin{aligned}  &   \langle A^{- \kappa } \llbracket (X^A_s + \textrm{e}^{- A^{1 - 2 \kappa } s} h_0) | (X^A_s + \textrm{e}^{- A^{1 - 2 \kappa } s} h_0) |^2 \rrbracket ,\nonumber \\  &   \quad \left. UA^{- \kappa } \llbracket (X^A_s + \textrm{e}^{- A^{1 - 2 \kappa } s} h_0) | (X^A_s + \textrm{e}^{- A^{1 - 2 \kappa } s} h_0) |^2 \rrbracket \right\rangle _{\mathbb {R}^4}. \end{aligned}$$

#### Proof

Thanks to Lemma 6.11 and Theorem A.4 (on the multiplication of distributions in Besov spaces), the expressions in Eqs. (6.23) and (6.24) can be defined in the following way: taking for example $\llbracket | (X^A_t + \textrm{e}^{- A^{1 - 2 \kappa } t} h_0) |^4 \rrbracket$, if we (formally) expand the fourth power and using the properties of Wick products with respect to addition we can define $\llbracket | (X^A_t + \textrm{e}^{- A^{1 - 2 \kappa } t} h_0) |^4 \rrbracket$

6.25 $$\begin{aligned} \begin{array}{l} \llbracket | (X^A_t + \textrm{e}^{- A^{1 - 2 \kappa } t} h_0) |^4 \rrbracket \\ \qquad :=\llbracket | X^A_t |^4 \rrbracket + 2 \llbracket | X^A_t |^2 X^A_t \rrbracket \cdot \textrm{e}^{- A^{1 - 2 \kappa } t} h_0 + 2 \llbracket | X^A_t |^2 \rrbracket | \textrm{e}^{- A^{1 - 2 \kappa } t} h_0 |^2\\ \hspace{3em} + 2 \llbracket X^{A, 1}_t X^{A, 2}_t \rrbracket (\textrm{e}^{- A^{1 - 2 \kappa } t} {\bar{h}}_0^1 \textrm{e}^{- A^{1 - 2 \kappa } t} {\bar{h}}_0^2) + \llbracket {\bar{X}}^{A, 1}_t {\bar{X}} ^{A, 2}_t \rrbracket (\textrm{e}^{- A^{1 - 2 \kappa } t} h_0^1 \textrm{e}^{- A^{1 - 2 \kappa } t} h_0^2)\\ \hspace{3em} + 2 (X^A_t \cdot \textrm{e}^{- A^{1 - 2 \kappa } t} h_0) | \textrm{e}^{- A^{1 - 2 \kappa } t} h_0 |^2 + | \textrm{e}^{- A^{1 - 2 \kappa } t} h_0 |^4. \end{array} \nonumber \\ \end{aligned}$$

The right hand side of the expression (6.25) is well-defined since $\llbracket | X^A_t |^4 \rrbracket$, $\llbracket | X^A_t |^2 X^A_t \rrbracket$, etc. are, by Lemma 6.11, random distributions in $B^{- \varepsilon }_{p, p} (\mathbb {T}^2 ; \mathcal {G}^p_X)$, meanwhile $\textrm{e}^{- A^{1 - 2 \kappa } t} h_0$, $| \textrm{e}^{- A^{1 - 2 \kappa } t} h_0 |^2$, etc. are random fields in $C^1 (\mathbb {T}^2 ; \mathcal {G}^{\infty }_X)$. Since $- \varepsilon + 1 > 0$ (for $\varepsilon$ small enough) and $\frac{1}{p} + \frac{1}{\infty } \leqslant 1$ (for any $p \geqslant 1$), by Theorem A.4, the products $\llbracket | X^A_t |^2 X^A_t \rrbracket \cdot \textrm{e}^{- A^{1 - 2 \kappa } t} h_0$, $\llbracket | X^A_t |^2 \rrbracket | \textrm{e}^{- A^{1 - 2 \kappa } t} h_0 |^2$, etc. are well-defined as random distribution in $B^{- \varepsilon }_{p, p} (\mathbb {T}^2 ; \mathcal {G}^{\infty }_X)$. In a similar way we can define all the other random distribution in expression (6.23) and (6.24).

By observing that

6.26 $$\begin{aligned}  &   \llbracket | P_N (X^A_t + \textrm{e}^{- A^{1 - 2 \kappa } t} h_0) |^4 \rrbracket \nonumber \\  &   \quad = \llbracket | P_N X^A_t |^4 \rrbracket + 2 \llbracket | P_N X^A_t |^2 P_N X^A_t \rrbracket \cdot \textrm{e}^{- A^{1 - 2 \kappa } t} P_N h_0 + 2 \llbracket | P_N X^A_t |^2 \rrbracket | \textrm{e}^{- A^{1 - 2 \kappa } t} P_N h_0 |^2\nonumber \\  &   \qquad + \llbracket P_N X^A_t \otimes P_N X^A_t \rrbracket \cdot (\textrm{e}^{- A^{1 - 2 \kappa } t} P_N h_0 \otimes \textrm{e}^{- A^{1 - 2 \kappa } t} P_N h_0) + | \textrm{e}^{- A^{1 - 2 \kappa } t} P_N (h_0) |^4.\nonumber \\ \end{aligned}$$

it is easy to see that, by Lemma 6.11, Theorem A.4 and the convergence of $\textrm{e}^{- A^{1 - 2 \kappa } t} P_N (h_0)$ to $\textrm{e}^{- A^{1 - 2 \kappa }} h_0$ in $C^{1 - \varepsilon } (\mathbb {T}^2 ; \mathcal {G}^{\infty }_X)$, as $N \rightarrow + \infty$, the expression at the right hand side of equation (6.26) converges to the sum (6.12), as $N \rightarrow + \infty$. The convergence of the other terms to the random processes in equations (6.23) and (6.24) can be done in a similar way. $\square$

We can now, prove that $Z^{N, h_0}_t$ has a limit as $N \rightarrow \infty$.

#### Lemma 6.13

Suppose that $\frac{19}{40}< \kappa < \frac{1}{2}$. Then, for any $p \geqslant 2$ we have that $Z^{N, h_0}_t \rightarrow Z_t^{h_0}$ in $C^0 (\mathbb {R}_+ ; \mathcal {G}^p_X)$ where $Z_t^{h_0}$ is as follows

6.27 $$\begin{aligned} Z_t^{h_0}= &   \exp \left( \lambda \int _{\mathbb {T}^2} \llbracket | (X^A_t + \textrm{e}^{- A^{1 - 2 \kappa } t} h_0) |^4 \rrbracket \textrm{d}x - \lambda \int _{\mathbb {T}^2} \llbracket | (X^A_0 + h_0) |^4 \rrbracket \textrm{d}x \right. \nonumber \\  &   + \lambda \int _0^t \int _{\mathbb {T}^2} \llbracket [(X^A_s + \textrm{e}^{- A^{1 - 2 \kappa } s} h_0) \cdot A^{1 - 2 \kappa } (X^A_s + \textrm{e}^{- A^{1 - 2 \kappa } s} h_0)] | X^A_t\nonumber \\  &   + \textrm{e}^{- A^{1 - 2 \kappa } s} h_0 |^2 \rrbracket \textrm{d}x \textrm{d}s \nonumber \\  &   - \frac{\lambda ^2}{2} \int _0^t \int _{\mathbb {T}^2} \langle A^{- \kappa } \llbracket (X^A_s + \textrm{e}^{- A^{1 - 2 \kappa } s} h_0) | (X^A_s + \textrm{e}^{- A^{1 - 2 \kappa } s} h_0) |^2 \rrbracket , \nonumber \\  &   \left. \phantom {\frac{\lambda ^2}{2}} UA^{- \kappa } \llbracket (X^A_s + \textrm{e}^{- A^{1 - 2 \kappa } s} h_0) | (X^A_s + \textrm{e}^{- A^{1 - 2 \kappa } s} h_0) |^2 \rrbracket \rangle _{\mathbb {R}^4} \textrm{d}x \textrm{d}t \right) ,\qquad \end{aligned}$$

where the expressions $\llbracket | (X^A_t + \textrm{e}^{- A^{1 - 2 \kappa } t} h_0) |^4 \rrbracket$, $\llbracket | (X^A_0 + h_0) |^4 \rrbracket$, etc. in formula (6.27) are defined as explained in Lemma 6.12.

#### Proof

We prove only that $\exp \left( \lambda \int _{\mathbb {T}^2} \llbracket P_N (| P_N (X^A_t + \textrm{e}^{- A^{1 - 2 \kappa } t} h_0) |^4) \rrbracket \textrm{d}x \right)$ converges to

6.28 $$\begin{aligned} \begin{array}{l} \exp \left( \lambda \int _{\mathbb {T}^2} (\llbracket | X^A_t |^4 \rrbracket + 2 \llbracket | X^A_t |^2 X^A_t \rrbracket \cdot \textrm{e}^{- A^{1 - 2 \kappa } t} h_0 + 2 \llbracket | X^A_t |^2 \rrbracket | \textrm{e}^{- A^{1 - 2 \kappa } t} h_0 |^2 \right. \\ \qquad \left. \phantom {\int _{\mathbb {T}^2}} + \llbracket X^A_t \otimes X^A_t \rrbracket \cdot (\textrm{e}^{- A^{1 - 2 \kappa } t} h_0 \otimes e^{- A^{1 - 2 \kappa } t} h_0) + | \textrm{e}^{- A^{1 - 2 \kappa } t} h_0 |^4) \textrm{d}x \right) \end{array} \nonumber \\ \end{aligned}$$

where

$$\begin{aligned} \begin{array}{l} \llbracket X^A_t \otimes X^A_t \rrbracket \cdot (\textrm{e}^{- A^{1 - 2 \kappa } t} h_0 \otimes \textrm{e}^{- A^{1 - 2 \kappa } t} h_0)\\ \quad = 2 \llbracket X^{A, 1}_t X^{A, 2}_t \rrbracket (\textrm{e}^{- A^{1 - 2 \kappa } t} {\bar{h}}_0^1 \textrm{e}^{- A^{1 - 2 \kappa } t} {\bar{h}}_0^2) + \llbracket {\bar{X}}^{A, 1}_t {\bar{X}} ^{A, 2}_t \rrbracket (\textrm{e}^{- A^{1 - 2 \kappa } t} h_0^1 \textrm{e}^{- A^{1 - 2 \kappa } t} h_0^2), \end{array} \end{aligned}$$

in $C^0 (\mathbb {R}_+; \mathcal {G}^p_X)$, since the convergence of all other terms can be proved in a similar way. By equation (6.26), in order to prove that $\exp \big ( \lambda \int _{\mathbb {T}^2} \llbracket P_N (| P_N (X^A_t + \textrm{e}^{- A^{1 - 2 \kappa } t} h_0) |^4) \rrbracket \textrm{d}x \big )$ converges to the exponential in (6.28), it is enough to prove that $\exp \big ( \lambda \int _{\mathbb {T}^2} \llbracket | P_N X^A_t |^4 \rrbracket \textrm{d}x \big )$ converges to $\exp \left( \lambda \int _{\mathbb {T}^2} \llbracket | X^A_t |^4 \rrbracket \textrm{d}x \right)$, that $\exp \left( 2 \lambda \int _{\mathbb {T}^2} \llbracket | P_N X^A_t |^2 P_N X^A_t \rrbracket \right. \left. \cdot \textrm{e}^{- A^{1 - 2 \kappa } t} P_N h_0 \textrm{d}x \right)$ converges to the quantity $\exp \left( \lambda \int _{\mathbb {T}^2} 2 \llbracket | X^A_t |^2 X^A_t \rrbracket \cdot \textrm{e}^{- A^{1 - 2 \kappa } t} h_0 \textrm{d}x \right)$, etc.

The convergence of $\exp \left( \lambda \int \llbracket | P_N X^A_t |^4 \rrbracket \textrm{d}x \right)$ to $\exp \left( \lambda \int \llbracket | X^A_t |^4 \rrbracket \textrm{d}x \right)$, has been proved in Theorem 6.3.

If we consider the term $\llbracket | P_N X^A_t |^2 P_N X^A_t \rrbracket \cdot \textrm{e}^{- A^{1 - 2 \kappa } t} P_N h_0$, by Lemma 6.11 and Theorem A.4, for any $k \in \mathbb {N}$ and $p \geqslant 2$, we have that

6.29 $$\begin{aligned}  &   \left\| \int \llbracket | P_N X^A_t |^2 P_N X^A_t \rrbracket \cdot \textrm{e}^{- A^{1 - 2 \kappa } t} P_N h_0 \textrm{d}x - \int \llbracket | X^A_t |^2 X^{A^{1 - 2 \kappa }}_t \rrbracket \cdot \textrm{e}^{- A^{1 - 2 \kappa } t} h_0 \textrm{d}x \right\| _{\mathbb {L}^{k p}}\nonumber \\  &   \quad \leqslant \Vert \llbracket | P_N X^A_t |^2 P_N X^A_t \rrbracket - \llbracket | X^A_t |^2 X^A_t \rrbracket \Vert _{B^{- \varepsilon }_{k p, k p}} \Vert h_0 \Vert _{C^1} \nonumber \\  &   \qquad + \Vert \llbracket | X^A_t |^2 X^A_t \rrbracket \Vert _{B^{- \varepsilon }_{k p, k p}} N^{(2 \varepsilon - 1)} \Vert h_0 \Vert _{C^1} \rightarrow 0 \end{aligned}$$

as $N \rightarrow \infty$. Furthermore, by inequality (6.15) and Theorem A.4, we get

6.30 $$\begin{aligned}  &   \left\| \sum _{k = 0}^n \frac{\lambda ^k}{k!} \left( \int \llbracket | P_N X^A_t |^2 P_N X^A_t \rrbracket \cdot \textrm{e}^{- A^{1 - 2 \kappa } t} P_N h_0 \textrm{d}x - \int \llbracket | X^A_t |^2 X^A_t \rrbracket \cdot \textrm{e}^{- A^{1 - 2 \kappa } t} h_0 \textrm{d}x \right) ^k \right\| _{\mathbb {L}^p} \nonumber \\  &   \quad \lesssim \sum _{k = 0}^n \frac{\lambda ^{\lambda }}{k!} \left\| \int \llbracket | P_N X^A_t |^2 P_N X^A_t \rrbracket \cdot \textrm{e}^{- A^{1 - 2 \kappa } t} P_N h_0 \textrm{d}x - \int \llbracket | X^A_t |^2 X^A_t \rrbracket \cdot \textrm{e}^{- A^{1 - 2 \kappa } t} h_0 \textrm{d}x \right\| _{\mathbb {L}^{k p}} \nonumber \\  &   \quad \lesssim \sum _{k = 0}^n \frac{\lambda ^k \Vert h_0 \Vert _{C^1}^k}{k!} (\Vert \llbracket | P_N X^A_t |^2 P_N X^A_t \rrbracket - \llbracket | X^A_t |^2 X^A_t \rrbracket \Vert _{B^{- \varepsilon }_{k p, k p}}^k + N^{k (2 \varepsilon - 1)} \Vert \llbracket | X^A_t |^2 X^A_t \rrbracket \Vert _{B^{- \varepsilon }_{k p, k p}}^k) \nonumber \\  &   \quad \lesssim \sum _{k = 0}^n C_p^k k^{(\nu (\kappa , \varepsilon ) - 1)}, \end{aligned}$$

where $C_p > 1$ is a suitable constant depending on *p*. Since $\sum _{k = 0}^n C_p^k k^{(\nu (\kappa , \varepsilon ) - 1)}$ is a convergent sequence, inequality (6.30) proves that $\exp \left( \lambda \int \llbracket | X^A_t |^2 X^A_t \rrbracket \cdot \textrm{e}^{- A^{1 - 2 \kappa } t} h_0 \textrm{d}x \right)$ is well-defined in $\mathcal {G}_X^p$. Furthermore, by the convergence of (6.29) and Lebesgue dominated convergence theorem, inequality (6.30) implies that $\exp \big ( \lambda \int \llbracket | P_N X^A_t |^2 P_N X^A_t \rrbracket \cdot \textrm{e}^{- A^{1 - 2 \kappa } t} P_N h_0 \textrm{d}x \big )$ converges to $\exp \left( \lambda \int \llbracket | X^A_t |^2 X^A_t \rrbracket \cdot \textrm{e}^{- A^{1 - 2 \kappa } t} h_0 \textrm{d}x \right)$ in $C^0 (\mathbb {R}_+ ; \mathcal {G}^p_X)$. In the same way, it is possible to prove that the exponential of every term in the sum (6.26) converges to the exponential of the corresponding term of equation (6.28). Since every term in the exponential is even, and thus the standard properties of the products of exponentials hold, and since we proved the convergence of each single exponential in $C^0 (\mathbb {R}_+ ; \mathcal {G}^p_X)$, for any arbitrary $p \geqslant 2$, the statement follows from Hölder’s inequality for the twisted spaces. $\square$

We have now all the tools for proving the convergence of weak solutions to equation (6.13) to the weak solution to equation (6.10).

#### Theorem 6.14

Let $\frac{19}{40}< \kappa < \frac{1}{2}$ and $h_0 \in C^1 (\mathbb {T}^2 ; \mathcal {G}^{\infty }_X)$. Then, for any $F \in \bigoplus _{n = 0}^k \Lambda ^n (\mathcal {S} (\mathbb {T}^2)^r)$ and any $t_1< \cdots < t_r \in \mathbb {R}_+$ we have

6.31 $$\begin{aligned} \lim _{N \rightarrow \infty } \omega _0 (F (\chi _{t_1}^{(N)}, \ldots , \chi _{t_r}^{(N)}))= &   \overline{\mathcal {E}}_0^Z (F (X^A_{t_1} + \textrm{e}^{- A^{1 - 2 \kappa } t_1} h_0, \ldots , X^A_{t_r} + \textrm{e}^{- A^{1 - 2 \kappa } t_1} h_0)) \nonumber \\= &   \mathcal {E}_0 (F (X^A_{t_1} + \textrm{e}^{- A^{1 - 2 \kappa } t_1} h_0, \ldots , X^A_{t_r} + \textrm{e}^{- A^{1 - 2 \kappa } t_1} h_0) Z_{t_r}), \nonumber \\ \end{aligned}$$

where $\chi ^{(N)}_t$ is the solution to equation (6.13).

#### Proof

The proof is a consequence of the representation of solutions to equation (6.13) given in Proposition 6.10 and of the convergence of the process $Z^{N, h_0}$ to $Z^{h_0}$ provided by Lemma 6.13. $\square$

## Besov spaces of distributions taking values in Banach spaces

In this appendix, we want to recall some results about Besov spaces of functions (or distributions) on $\mathbb {T}^d$ taking values in a Banach space $\mathcal {A}$. The results of this section can be found in  [3, 4], where the theory of Besov spaces taking values in a Banach space has been developed.

We denote by $\mathcal {S} (\mathbb {T}^d)$ the space of smooth functions defined on $\mathbb {T}^d$ and equipped with the set of seminorms

$$\begin{aligned} \Vert f \Vert _{\alpha } :=\Vert | D^{\alpha } f | \Vert _{L^{\infty } (\mathbb {T}^d)} < \infty , \end{aligned}$$

where $\alpha \in \mathbb {N}^d$. We denote by $\mathcal {S}' (\mathbb {T}^d)$ the strong dual of $\mathcal {S} (\mathbb {T}^d)$ with respect to the topology induced by the seminorms $\Vert \cdot \Vert _{\alpha }$. If *A* is a Banach space and *B* a nuclear space, we denote by $B {\hat{\otimes }} A$ the completion of $B \otimes A$ with respect to the natural metric of the algebraic tensor product on $B \otimes A$. Such a completion is unique up to an isomorphism. Using this notation we define

$$\begin{aligned} \mathcal {S} (\mathbb {T}^d; \mathcal {A}): =\mathcal {S} (\mathbb {T}^d) {\hat{\otimes }} \mathcal {A}, \quad \mathcal {S}' (\mathbb {T}^d; \mathcal {A}): =\mathcal {S}' (\mathbb {T}^d) {\hat{\otimes }} \mathcal {A}, \end{aligned}$$

where $\mathcal {A}$ is a Banach space. It is important to note that

$$\begin{aligned} \mathcal {S} (\mathbb {T}^d; \mathcal {A}) \overset{d}{\hookrightarrow } \mathcal {S}' (\mathbb {T}^d; \mathcal {A}) \end{aligned}$$

where the arrow means that a space is continuously embedded and dense in the following one.

### [Style3 Style3]Remark A.1

Let $A_1$, $A_2$ and $A_3$ be three Banach spaces, for which a product operation $\cdot : A_1 \times A_2 \rightarrow A_3$ (which is a continuous bilinear function) is defined. Then, for any $i = 1, 2, 3$, it is possible to define uniquely $\langle \cdot , \cdot \rangle : \mathcal {S} (\mathbb {T}^d ; \mathcal {A}_1) \times \mathcal {S}' (\mathbb {T}^d ; \mathcal {A}_2) \rightarrow \mathcal {A}_3$, $\cdot : \mathcal {S} (\mathbb {T}^d ; \mathcal {A}_2) \times \mathcal {S}' (\mathbb {T}^d ; \mathcal {A}_2) \rightarrow \mathcal {S}' (\mathbb {T}^d ; \mathcal {A}_3)$, $\cdot : \mathcal {S} (\mathbb {T}^d) \times \mathcal {S}' (\mathbb {T}^d ; \mathcal {A}_i) \rightarrow \mathcal {S}' (\mathbb {T}^d ; \mathcal {A}_i)$, $\mathord {*}: \mathcal {S} (\mathbb {T}^d) \times \mathcal {S}' (\mathbb {T}^d ; \mathcal {A}_i) \rightarrow \mathcal {S} (\mathbb {T}^d ; \mathcal {A}_i)$ and $D^{\alpha } : \mathcal {S}' (\mathbb {T}^d ; \mathcal {A}) \rightarrow \mathcal {S}' (\mathbb {T}^d ; \mathcal {A})$ (where $\alpha \in \mathbb {N}^d$) which extend in a continuous way the following operations: any $f \in \mathcal {S} (\mathbb {T}^d)$, $u \in \mathcal {S}' (\mathbb {T}^d)$ and $a \in A_i$, $a_1 \in A_1$, $a_2 \in A_2$ we have

$$\begin{aligned} \begin{array}{rll} \langle f \otimes a_1, u \otimes a_2 \rangle &  = &  \langle f, u \rangle a_1 a_2\\ (f \otimes a_1) \cdot (u \otimes a_2) &  = &  (f u) \otimes (a_1 a_2)\\ f \cdot (u \otimes a) &  = &  (f u) \otimes a\\ f \mathord {*}(u \otimes a) &  = &  (f \mathord {*}u) \otimes a\\ D^{\alpha } (u \otimes a) &  = &  (D^{\alpha } u) \otimes a \end{array} \end{aligned}$$

where $\langle f, u \rangle$ is the normal pairing in $\mathcal {S} (\mathbb {T}^d) \times \mathcal {S}' (\mathbb {T}^d)$, (*fu*) is the product in $\mathcal {S} (\mathbb {T}^d) \times \mathcal {S}' (\mathbb {T}^d)$, $(f \mathord {*}u)$ is the convolution in $\mathcal {S} (\mathbb {T}^d) \times \mathcal {S}' (\mathbb {T}^d)$ and $D^{\alpha }$ is the $\alpha$ derivative in $\mathcal {S}' (\mathbb {T}^d)$ (see [4] Appendix 1).

We recall the definition of Littlewood–Paley decomposition on the torus $\mathbb {T}^d$. Let $\chi , \varphi$ be smooth non-negative functions from $\mathbb {R}^d$ to $\mathbb {R}$ such that

- $$\operatorname {supp} (\chi ) \subset B_{\frac{4}{3}} (0)$$ and $$\operatorname {supp} (\varphi ) \subset B_{\frac{8}{3}} (0) {\setminus } B_{\frac{3}{4}} (0)$$,
- $\chi , \varphi \leqslant 1$ and $\chi (y) + \sum _{j \ge 0} \varphi (2^{- j} y) = 1$ for any $y \in \mathbb {R}^n$,
- $$\operatorname {supp} (\chi ) \cap \operatorname {supp} (\varphi (2^{- i} \cdot )) = \emptyset $$ for $$i \geqslant 1$$,
- $$\operatorname {supp} (\varphi (2^{- j} \cdot )) \cap \operatorname {supp} (\varphi (2^{- i} \cdot )) = \varnothing $$ if $$| i - j | > 1$$,

where by $B_r (x)$ we denote the ball centered at $x \in \mathbb {R}^d$ and of radius $r > 0$. We introduce the following notation: $\varphi _{- 1} = \chi$, $\varphi _j (\cdot ) = \varphi (2^{- j} \cdot )$, $K_j = \mathcal {F}^{- 1} (\varphi _j |_{\mathbb {Z}^d}) \in \mathcal {S} (\mathbb {T}^d)$.

If $v \in \mathcal {S}' (\mathbb {T}^d ; \mathcal {A})$ and if $i \in \mathbb {Z}$, $i \geqslant - 1$ we define the *i*th Littlewood–Paley block as follows

$$\begin{aligned} \Delta _i v = K_i \mathord {*}v \in \mathcal {S} (\mathbb {T}^d; \mathcal {A}). \end{aligned}$$

Then, if $s \in \mathbb {R}$, $p, q \in [1, + \infty ]$, we define the function

$$\begin{aligned} \Vert v \Vert _{B^s_{p, q} (\mathbb {T}^d; \mathcal {A})} = \left( \sum _{j = - 1}^{+ \infty } 2^{j s q} \Vert \Delta _j v \Vert _{L^p (\mathbb {T}^d; \mathcal {A})}^q \right) ^{1 / q}, \end{aligned}$$

when $q \in [1, + \infty )$ and $\Vert v \Vert _{B^s_{p, + \infty } (\mathbb {T}^d ; \mathcal {A})} = \sup _j (2^{j s} \Vert \Delta _j v \Vert _{L^p (\mathbb {T}^d, \mathcal {A})})$, where $\Vert \cdot \Vert _{L^p (\mathbb {T}^d ; \mathcal {A})}$ is the norm in the space $L^p (\mathbb {T}^d; \mathcal {A})$ that is,

$$\begin{aligned} \Vert f \Vert _{L^p (\mathbb {T}^d; \mathcal {A})} = \left( \int _{\mathbb {T}^d} \Vert f (y) \Vert _{\mathcal {A}}^p \textrm{d}y \right) ^{1 / p} \end{aligned}$$

for $$p \in [1, + \infty )$$, and

$$\begin{aligned} \Vert f \Vert _{L^{\infty } (\mathbb {T}^d; \mathcal {A})} = \sup _{y \in \mathbb {T}^d} \Vert f (y) \Vert _{\mathcal {A}}, \end{aligned}$$

for $p = + \infty$. For any $v \in \mathcal {S} (\mathbb {T}^d ; \mathcal {A})$ the norm $\Vert v \Vert _{B^s_{p, q} (\mathbb {T}^d; \mathcal {A})} < + \infty$ is finite. Then we look at $B^s_{p, q} (\mathbb {T}^d; \mathcal {A})$ as the closure of $\mathcal {S} (\mathbb {T}^d ; \mathcal {A})$ in $\mathcal {S}' (\mathbb {T}^d ; \mathcal {A})$ with respect to the norm $\Vert \cdot \Vert _{B^s_{p, q} (\mathbb {T}^d ; \mathcal {A})}$. Hereafter, if $s \in \mathbb {R}$, $p, q \in [1, + \infty ]$, we use the following notation $\mathcal {C}^s (\mathbb {T}^d ; \mathcal {A}) :=B^s_{\infty , \infty } (\mathbb {T}^d ; \mathcal {A})$, $B^s_{p, q} :=B^s_{p, q} (\mathbb {T}^d; \mathbb {R})$ etc.

In this paper we need some results.

### Theorem A.2

Consider $m > 0$, $\alpha , s \in \mathbb {R}$, $p, q \in [1, + \infty ]$, such that  then we have that $(- \Delta + m)^{- \alpha }$, where $\Delta$ is the standard Laplacian on $\mathbb {T}^d$, is a continuous linear map from $B^s_{p, q} (\mathbb {T}^d ; \mathcal {A})$ into $B^{s + \alpha }_{p, q} (\mathbb {T}^d ; \mathcal {A})$.

### Proof

This is exactly  [4, Theorem 5.3.2] for the case $\mathbb {R}^d$. The theorem on the torus can be proved in a similar way. $\square$

### Theorem A.3

Consider $s_1 \geqslant s_2 \in \mathbb {R}$, $p_1, p_2 \in [1, + \infty ]$, and let $A_1 \subset A_2$ be two Banach spaces (where the inclusion is continuous). Suppose further that $s_1 - \frac{d}{p_1} > s_2 - \frac{d}{p_2}$, then we have the following continuous inclusion

$$\begin{aligned} B^{s_1}_{p_1, p_1} (\mathbb {T}^d; A_1) \subset B^{s_2}_{p_2, p_2} (\mathbb {T}^d; A_2). \end{aligned}$$

### Proof

The proof can be found in  [4]. $\square$

### Theorem A.4

Let $A_1$, $A_2$ and $A_3$ be three Banach spaces, for which a product operation $\cdot : A_1 \times A_2 \rightarrow A_3$ (which is a continuous bilinear function) is defined. Consider $s_1, s_2 \in \mathbb {R}$, such that $s_1 > 0$, $s_2 \leqslant 0$, and $s_1 + s_2 > 0$. Consider $p_1, p_2, p_3 \in [1, + \infty ]$ such that $\frac{1}{p_1} + \frac{1}{p_2} = \frac{1}{p_3}$, then we have that the product $\cdot : \mathcal {S} (\mathbb {T}^d ; \mathcal {A}_1) \times \mathcal {S} (\mathbb {T}^d ; \mathcal {A}_2) \rightarrow \mathcal {S} (\mathbb {T}^d ; \mathcal {A}_3)$ can be (uniquely) extended in a continuous way to a product from $\mathcal {B}^{s_1}_{p_1, p_1} (\mathbb {T}^d ; \mathcal {A}_1) \times B^{s_2}_{p_2, p_2} (\mathbb {T}^d ; \mathcal {A}_2)$ into $B^{s_2}_{p_3, p_3} (\mathbb {T}^d ; \mathcal {A}_3)$, furthermore for any $v_1 \in \mathcal {B}^{s_1}_{p_1, p_1} (\mathbb {T}^d ; \mathcal {A}_1)$ and $v_2 \in B^{s_2}_{p_2, p_2} (\mathbb {T}^d ; \mathcal {A}_2)$ we have

$$\begin{aligned} \Vert v_1 \cdot v_2 \Vert _{B^{s_2}_{p_3, p_3} (\mathbb {T}^d; \mathcal {A}_3)} \lesssim \Vert v_1 \Vert _{\mathcal {B}^{s_1}_{p_1, p_1} (\mathbb {T}^d; \mathcal {A}_1)} \Vert v_2 \Vert _{B^{s_2}_{p_2, p_2} (\mathbb {T}^d; \mathcal {A}_2)}. \end{aligned}$$

### Proof

The proof can be found in  [4]. $\square$

## Twisted $L^2$ spaces and unbounded operators

In this section, we investigate the link between unbounded operators and non-commutative $L^p$ spaces. Recall that $\mathcal {H} :=L^2 (\mathcal {M})$ equipped with the inner product defined by Haagerup’s trace $\langle x, y \rangle _{\mathcal {H}} :=\operatorname {tr}_{\textrm{H}} (x^{*} y)$ is a Hilbert space, see Proposition 2.11. It is convenient to represent the vNa $\mathcal {M}$ as acting on $\mathcal {H}$ by left multiplication, that is, $\pi _l (x) \xi : = x \xi$, for any $x \in \mathcal {M}$ and $\xi \in \mathcal {H}$, giving rise to the so-called standard form $\{ \pi _l (\mathcal {M}), \mathcal {H}, J, \mathcal {H}_+ \}$ with conjugation $J \xi = \xi ^{*}$, see  [20]. For the sake of simplicity, we will henceforth write $\mathcal {M} \equiv \pi _l (\mathcal {M})$. The element $D^{\frac{1}{2}} \in \mathcal {H}_+$ is the unit cyclic separating vector for $\mathcal {M}$ associated with the state $\omega$, so that $\mathcal {M}$ is in the GNS representation. Recall that $\mathcal {D} :=D^{\frac{1}{2}} \mathcal {M}$ is dense in $\mathcal {H}$, see Lemma 2.2

Whereas $\mathcal {M}$ acts as bounded multiplication operators on $\mathcal {H}$, we would like to extend this identification to elements of $L^p (\mathcal {M})$ for any $1 \leqslant p < \infty$ as follows.

### Definition B.1

If $x \in L^p (\mathcal {M})$ we define $\operatorname {Op} (x) : \mathcal {D} \rightarrow \mathcal {H}$ by

$$\begin{aligned} \operatorname {Op}_p (x) D^{\frac{1}{2}} y :=xD^{\frac{1}{2} - \frac{1}{p}} y. \end{aligned}$$

This is an extension of the multiplication by elements in $\mathcal {M}$ since in fact $\operatorname {Op}_p \left( \mathcal {M}D^{\frac{1}{p}} \right) \equiv \mathcal {M}$, that is,

$$\begin{aligned} \operatorname {Op}_p \left( xD^{\frac{1}{p}} \right) D^{\frac{1}{2}} y = xD^{\frac{1}{2}} y. \qquad \forall x, y \in \mathcal {M}. \end{aligned}$$

Unfortunately, the operators so defined lack some important properties, e.g., they might not be closable and this is due to the fact that if $x_n D^{\frac{1}{p}} \rightarrow x \in L^p (\mathcal {M})$, with $x_n \in \mathcal {M}_a$, it is not clear whether also $x_n^{*} D^{\frac{1}{p}}$ has a limit, or equivalently if $[x_n]_{\frac{1}{p}} D^{\frac{1}{p}}$ converges.

### Lemma B.2

Let $x \in L^p (\mathcal {M})$ be such that there exists a sequence $x_n D^{\frac{1}{p}} \rightarrow x$ for which $x_n^{*} D^{\frac{1}{p}}$ is convergent in $L^p (\mathcal {M})$. Then, $\operatorname {Op}_p (x)$ is a closable operator.

The proof is the same as for Lemma B.4 proposed below. The difficulty discussed above motivates us consider spaces of unbounded operators where the twisted sequence converges as well, that is, the $\mathbb {L}^p$ space introduced in Sect. 2.3.

### Definition B.3

Let $1 \leqslant p \leqslant \infty$. For any $x \in \mathbb {L}^p (\mathcal {M})$ define the operator $\mathbb {O}\textrm{p}_p (x) : \mathcal {D} \rightarrow \mathcal {H}$ by

$$\begin{aligned} \mathbb {O}\textrm{p}_p (x) D^{\frac{1}{2}} y :=L^2 - \lim _{n \rightarrow \infty } x_n D^{\frac{1}{2}} y, \end{aligned}$$

where $(x_n) \subset \mathcal {M}_a$, $x_n \rightarrow x$ in the $\mathbb {L}^p$ topology.

This definition is meaningful because $x_n \rightarrow x$ in the $\mathbb {L}^p$ topology implies convergence of the sequence $x_n D^{\frac{1}{p}}$. Thus, by Hölder’s inequality the $L^2 -$limit exists and does not depend on the sequence $(x_n)$. We can prove that such operators are closable.

### Lemma B.4

Let $1 \leqslant p < \infty$. For any $x \in \mathbb {L}^p (\mathcal {M})$ the operator $\mathbb {O}\textrm{p}_p (x)$ is closable.

### Proof

For any $a, b \in \mathcal {M}_a$ by continuity we have

$$\begin{aligned} \left\langle D^{\frac{1}{2}} a, \mathbb {O}\textrm{p}_p (x) D^{\frac{1}{2}} b \right\rangle _{\mathcal {H}}= &   \lim _{n \rightarrow \infty } \left\langle D^{\frac{1}{2}} a, x_n D^{\frac{1}{2}} b \right\rangle _{\mathcal {H}} = \lim _{n \rightarrow \infty } \left\langle x_n^{*} D^{\frac{1}{2}} a, D^{\frac{1}{2}} b \right\rangle _{\mathcal {H}}\\= &   \left\langle \mathbb {O}\textrm{p}_p (x^{*}) D^{\frac{1}{2}} a, D^{\frac{1}{2}} b \right\rangle _{\mathcal {H}} \end{aligned}$$

where we crucially used that $x^{*}_n$ converges in $\mathbb {L}^p$ to $x^{*}$. In other words, we proved that $\mathbb {O}\textrm{p}_p (x)^{*} \supset \mathbb {O}\textrm{p}_p (x^{*})$ the latter being densely defined on $\mathcal {D}$, hence the claim. $\square$

We denote by $\overline{\mathbb {O}\textrm{p}_p} (x)$ the closure of $\mathbb {O}\textrm{p}_p (x)$ and its domain by $\mathcal {D} (x) \supset \mathcal {D}$.

### Remark B.5

A priori we do not know if $\mathcal {D}$ is a *core* for $\operatorname {Op} (x)$, that is, if *b* is another closed operator which coincide with $a :=\overline{\operatorname {Op}} (x)$ on $\mathcal {D}$ then $a = b$. In principle there could be a closed operator with a larger domain. We will see below that this does not happen under some additional assumptions.

Let us now investigate sufficient conditions to have self-adjoint operators associated with elements in twisted $\mathbb {L}^p$ spaces. Recall that if $x \in \mathcal {M}_a$, $[x]_t^{*} = [x^{*}]_{- t}$ and this will pose a problem in our computations. This will require some strong condition for proving that a sequence $(x_n)_n \subset \mathcal {M}_a$ self-adjoint converges to a self-adjoint operator on $\mathcal {H}$.

### Theorem B.6

Let $2 \leqslant p \leqslant \infty$. Let $x \in L^p (\mathcal {M})$ and assume that there exists $(x_n)_n \subset \mathcal {M}_a$ self-adjoint such that $x_n D^{\frac{1}{p}}$ converges to *x* in $L^p$ and moreover that $U_n (t) :=\exp (\textrm{i}x_n t) \in \mathcal {M}_a$ satisfies

B.1 $$\begin{aligned} \sup _n \left\| [U_n (s)]_{- \frac{1}{p}} D^{\frac{1}{2} - \frac{1}{p}} \right\| _{\frac{2 p}{p - 2}} < \infty . \end{aligned}$$

Then, $\operatorname {Op}_p (x)$ is essentially self-adjoint, affiliated with $\mathcal {M}$ and if we denote by $\mu$ the law of $\operatorname {Op}_p (x)$ under $\omega$, i.e. the unique Radon measure $\mu$ for which

$$\begin{aligned} \omega (g (\operatorname {Op}_p (x))) = \int g (y) \mu (\textrm{d}y) \end{aligned}$$

for $g \in C (\mathbb {R})$, then we have

B.2 $$\begin{aligned} \int y^2 \mu (\textrm{d}y) = \left\| xD^{\frac{1}{2} - \frac{1}{p}} \right\| _{\mathcal {H}}^2 < \infty . \end{aligned}$$

### Proof

We want to construct an unbounded self-adjoint operator $X \supseteq \operatorname {Op}_p (x)$ acting on $\mathcal {H}$ which represent the limit of the sequence $(x_n)_n \subset \mathcal {M}_a$. To do so, we observe that $\frac{\textrm{d}}{\textrm{d}t} [U_n (t) U_m (t)^{*}] = \textrm{i}U_n (t) (x_n - x_m) U_m (t)^{*}$ and thus, by the fundamental theorem of calculus

$$\begin{aligned} U_n (t) - U_m (t) = \textrm{i}\int _0^t U_n (s) (x_n - x_m) U_m (t - s) \textrm{d}s, \end{aligned}$$

the integral being intended in the Bochner sense. Since $x_m$ is analytic, so is $U_m (t)$, $[U_m (s)]_{- \frac{1}{p}}$ is well-defined and by assumption $\sup _n \left\| [U_n (s)]_{- \frac{1}{p}} D^{\frac{1}{2} - \frac{1}{p}} \right\| _{\frac{2 p}{p - 2}} \lesssim 1$. Thus, we write

$$\begin{aligned} U_n (s) (x_n - x_m) U_m (t - s) D^{\frac{1}{2}} a = U_n (s) (x_n - x_m) D^{\frac{1}{p}} [U_m (t - s)]_{- \frac{1}{p}} D^{\frac{1}{2} - \frac{1}{p}} a, \end{aligned}$$

and by Hölder’s inequality

B.3 $$\begin{aligned} \left\| [U_n (t) - U_m (t)] D^{\frac{1}{2}} a \right\| _{\mathcal {H}} \lesssim t \Vert a \Vert \left\| (x_n - x_m) D^{\frac{1}{p}} \right\| _p \end{aligned}$$

which shows that $\left( U_n (t) D^{\frac{1}{2}} a \right) _n$ is a Cauchy sequence in $\mathcal {H}$, so that we can define $U (t) : \mathcal {D} \rightarrow \mathcal {H}$ by

$$\begin{aligned} U (t) D^{\frac{1}{2}} a :=\lim _{n \rightarrow \infty } U_n (t) D^{\frac{1}{2}} a. \end{aligned}$$

By continuity, we have

$$\begin{aligned} \left\| U (t) D^{\frac{1}{2}} a \right\| _{\mathcal {H}} = \lim _{n \rightarrow \infty } \left\| U_n (t) D^{\frac{1}{2}} a \right\| _{\mathcal {H}} \leqslant \left\| D^{\frac{1}{2}} a \right\| _{\mathcal {H}}, \end{aligned}$$

so that *U*(*t*) can be extended by continuity to a bounded operator on $\mathcal {H}$ with $\Vert U (t) \Vert _{\mathcal {H} \rightarrow \mathcal {H}} = 1$. One can also prove that $U (t)^{*} = U (- t)$ and that $U (t) U (s) = U (t + s)$, the latter as a consequence of the identity

$$\begin{aligned} U (t) U (s) - U_n (t + s) = (U (t) - U_n (t)) U (s) + U_n (t) (U (s) - U_n (s)) \end{aligned}$$

where $$ U_n (t + s) = U_n (t) U_n (s)$$ was used. Thus $$(U (t))_t$$ is a one-parameter unitary group. We can prove strong continuity: in fact, by continuity and by the same argument that lead us to (B.3), we have

$$\begin{aligned} \Vert [1 - U (t)] \varphi \Vert _{\mathcal {H}}  &   \leqslant \left\| [1 - U (t)] D^{\frac{1}{2}} a \right\| _{\mathcal {H}} + {\Vert [1 - U (t)] \Vert _{\mathcal {H} \rightarrow \mathcal {H}}} \left\| \varphi - D^{\frac{1}{2}} a \right\| _{\mathcal {H}}\\  &   \lesssim t \Vert a \Vert + \left\| \varphi - D^{\frac{1}{2}} a \right\| _{\mathcal {H}}, \end{aligned}$$

and the r.h.s. can be made arbitrarily small by choosing *a* such that $\left\| \varphi - D^{\frac{1}{2}} a \right\| _{\mathcal {H}}$ is small and then *t* small depending on *a*. By Stone’s theorem, there exists a self-adjoint operator $X: \mathcal {D} (X) \rightarrow \mathcal {H}$, $\mathcal {D} (X)$ being the domain of *X*, such that $U (t) = \textrm{e}^{\textrm{i}Xt}$. It is easy to see that *X* is affiliated with $\mathcal {M}$ since this is the case for $(U (t))_t$. For any $D^{\frac{1}{2}} a \in \mathcal {D}$, we have

$$\begin{aligned} (U (t) - 1) D^{\frac{1}{2}} a= &   \lim _{n \rightarrow \infty } \textrm{i}\int _0^t U_n (r) x_n D^{\frac{1}{2}} a \textrm{d}r = \textrm{i}\int _0^t U (r) xD^{\frac{1}{2} - \frac{1}{p}} a \textrm{d}r\\= &   \textrm{i}\int _0^t U (r) \operatorname {Op}_p (x) \textrm{D}^{\frac{1}{2}} a \textrm{d}r, \end{aligned}$$

so that $\mathcal {D} \subseteq \mathcal {D} (X)$ and $X \supseteq \operatorname {Op}_p (x)$. Furthermore, by Hölder’s inequality

$$\begin{aligned} \left\| x_n D^{\frac{1}{2}} a - X D^{\frac{1}{2}} a \right\| _{\mathcal {H}} \leqslant \left\| x_n D^{\frac{1}{p}} - x \right\| _p \Vert a \Vert \rightarrow 0 \qquad \forall D^{\frac{1}{2}} a \in \mathcal {D}. \end{aligned}$$

We shall now show that $\mathcal {D}$ is a core for *X*. Assuming by contradiction that the closure $Y :=\overline{X|_{\mathcal {D}}}$ is different from *X*, we show that the one-parameter groups they generate are equal on $\mathcal {D}$. By the above considerations, for any $a \in \mathcal {M}$

B.4 $$\begin{aligned} \textrm{e}^{\textrm{i}Xs} \textrm{D}^{\frac{1}{2}} {a = \lim _{n \rightarrow \infty }} \textrm{e}^{\textrm{i}x_n s} \textrm{D}^{\frac{1}{2}} {a = \lim _{n \rightarrow \infty }} \textrm{D}^{\frac{1}{2}} [\textrm{e}^{\textrm{i}x_n s}]_{1 / 2} a, \end{aligned}$$

Furthermore, since $Y - X$ is identically null on $\mathcal {D}$, we have that ${\lim _{n \rightarrow \infty }} (Y - X) \textrm{D}^{\frac{1}{2}} [\textrm{e}^{\textrm{i}x_n s}]_{1 / 2} a = 0$. Accordingly, since $Y - X$ is a closed operator, by (B.4) we obtain

$$\begin{aligned} (Y - X) \textrm{e}^{\textrm{i}Xs} \textrm{D}^{\frac{1}{2}} {a = \lim _{n \rightarrow \infty }} (Y - X) \textrm{D}^{\frac{1}{2}} [\textrm{e}^{\textrm{i}x_n s}]_{1 / 2} a = 0, \end{aligned}$$

and therefore, by the fundamental theorem of calculus

$$\begin{aligned} (\textrm{e}^{\textrm{i}Yt} - \textrm{e}^{\textrm{i}Xt}) \textrm{D}^{\frac{1}{2}} a = \textrm{i}\int \textrm{e}^{\textrm{i}Y (t - s)} (Y - X) \textrm{e}^{\textrm{i}Xs} \textrm{D}^{\frac{1}{2}} a \textrm{d}s = 0. \end{aligned}$$

Since $\mathcal {D}$ is dense, by boundedness the two groups coincide on the whole $\mathcal {H}$, that is, $Y = X$.

By the functional calculus we can define $f (X) \in \mathcal {M}$ for all $f \in C (\mathbb {R})$ and then

$$\begin{aligned} \left\| f (X) D^{\frac{1}{2}} \right\| _{\mathcal {H}}^2 = \omega (| f (X) |^2) = \int | f (y) |^2 \mu (\textrm{d}y) \end{aligned}$$

where $\mu$ is the law of *x* under $\omega$, i.e. the random measure such that

$$\begin{aligned} \int g (y) \mu (\textrm{d}y) = \omega (g (X)) = \left\langle D^{\frac{1}{2}}, g (X) D^{\frac{1}{2}} \right\rangle _{\mathcal {H}} \end{aligned}$$

for all $g \in C (\mathbb {R})$. Taking $f \rightarrow \operatorname {Id}$ we obtain that

$$\begin{aligned} \left\| xD^{\frac{1}{2} - \frac{1}{p}} \right\| _{\mathcal {H}} = \left\| XD^{\frac{1}{2}} \right\| _{\mathcal {H}} = \left[ \int y^2 \mu (\textrm{d}y) \right] ^{\frac{1}{2}}. \end{aligned}$$

$$\square $$

### Remark B.7

Any self-adjoint operator *X* for which $\int y^2 \mu (\textrm{d}y) < \infty$ gives rise to an element of $\mathcal {H}$. To prove this, note that $f (X) D^{\frac{1}{2}} \in \mathcal {H}$ and

$$\begin{aligned} \left\| (f (X) - g (X)) D^{\frac{1}{2}} \right\| _{\mathcal {H}}^2 = \int | f (y) - g (y) |^2 \mu (\textrm{d}y) \end{aligned}$$

so if $f_n (y) \rightarrow y$ in $L^2 (\mu )$ we have also that $f_n (X) D^{\frac{1}{2}} \rightarrow x$ in $\mathcal {H}$. Finally, to see that $x = X D^{\frac{1}{2}}$, if $\varphi \in \operatorname {Dom} (X)$ we have $\left\langle \varphi , f_n (X) D^{\frac{1}{2}} \right\rangle _{\mathcal {H}} = \left\langle f_n (X) \varphi , D^{\frac{1}{2}} \right\rangle _{\mathcal {H}}$ and therefore $\langle \varphi , x \rangle _{\mathcal {H}} = \left\langle X \varphi , D^{\frac{1}{2}} \right\rangle _{\mathcal {H}}$ which shows that $D^{\frac{1}{2}} \in \mathcal {D} (X^{*}) =\mathcal {D} (X)$ and that $x = X D^{\frac{1}{2}}$.

We note that (B.2) is particularly interesting in the case $p = 2$ since it establishes the link between the $L^2$ norm of the operator and the expectation of $y \mapsto y^2$. To obtain a link with the $L^p$ norm, we need to intertwine *D* with *x* since in fact

$$\begin{aligned} \int | y |^p \mu (\textrm{d}y) = \operatorname {tr}_{\textrm{H}} \left( \left| xD^{- \frac{1}{p}} \right| ^p D \right) . \end{aligned}$$

On the other hand, this can be achieved in the twisted setting.

### Corollary B.8

Let $p \in [2, \infty ]$ and let $x \in \mathbb {L}^p$. Assume that there exists $(x_n) \subset \mathcal {M}_a$ self-adjoint such that $x_n \rightarrow x$ in the $\mathbb {L}^p$ topology and assume that $U_n (t) :=\exp (\textrm{i}x_n t)$ satisfies (B.1). Then, $\mathbb {O}\textrm{p}_p (x)$ is essentially self-adjoint, affiliated with $\mathcal {M}$ and if we denote by $\mu$ its law under $\omega$, compare with Theorem B.6, we have

$$\begin{aligned} \int y^2 \mu (\textrm{d}y) = \left\| xD^{\frac{1}{2}} \right\| _{\mathcal {H}}^2 < \infty . \end{aligned}$$

Furthermore, if $p = 2 n \in 2\mathbb {N}$, we also have

$$\begin{aligned} \int | y |^{2 n} \mu (\textrm{d}y) \leqslant \Vert x \Vert _{\mathbb {L}^{2 n}}^{2 n} < \infty . \end{aligned}$$

### Proof

We only need to prove the last claim. For $p = 2 n \in 2\mathbb {N}$ we have

$$\begin{aligned} \int | y |^{2 n} \mu (\textrm{d}y) = \operatorname {tr}_{\textrm{H}} (| x |^{2 n} D) = \operatorname {tr}_{\textrm{H}} (T^{(1 / 2 n)}_{\tau _1} (x^{*}) T^{(1 / 2 n)}_{\tau _2} (x) \cdots T^{(1 / 2 n)}_{\tau _{2 n}} (x)) \leqslant \Vert x \Vert _{\mathbb {L}^{2 n}}^{2 n}. \end{aligned}$$

$$\square $$

## Footnotes

1 In the GNS construction $(\mathcal {H}_{\omega }, \pi _{\omega }, \Omega _{\omega })$ $\omega (\cdot ) = \langle \Omega _{\omega } \cdot \Omega _{\omega } \rangle$ this means that $\Omega _{\omega }$ is cyclic and separating for $\pi _{\omega } (\mathcal {M})$.

2 Recall that the polar decomposition of $\varphi \in \mathcal {M}_{*}$ is obtained via duality, see  [56].

3 In general, the expression should be intended as a suitable regularization of ${\tilde{\omega }} (x) = \tau \left( D^{\frac{1}{2}} xD^{\frac{1}{2}} \right)$, for any $x \in \widetilde{\mathcal {M}}^+$, see  [45], unless $\omega$ is finite, that is, $\omega (\mathbb {1}) < \infty$.

4 In Sect. 4 we will introduce Grassmann random variables within the Araki–Wyss factors.
